# Expression Profiles and Functional Analysis of Plasma Exosomal Circular RNAs in Acute Myocardial Infarction

**DOI:** 10.1155/2022/3458227

**Published:** 2022-10-01

**Authors:** Guo-dong He, Jie Li, Zhi-qiang Nie, Shuo Sun, Ying-qing Feng, Yu-qing Huang

**Affiliations:** ^1^Department of Cardiology, Guangdong Provincial People's Hospital, Guangdong Academy of Medical Sciences, Guangzhou 510080, China; ^2^Research Department of Medical Sciences, Guangdong Provincial People's Hospital, Guangdong Academy of Medical Sciences, Guangzhou 510080, China

## Abstract

Acute myocardial infarction (AMI) is a common cardiovascular disease with high rates of morbidity and mortality globally. The dysregulation of circular RNAs (circRNAs) has been shown to be closely related to various pathological aspects of AMI. However, the function of exosomal circRNAs in AMI has yet to be investigated. The purpose of this study was to investigate the expression profiles of plasma exosomal circRNAs in AMI and explore their potential functionality. The expression profiles of plasma exosomal circRNAs in patients with AMI, stable coronary heart atherosclerotic disease (CAD), and healthy controls were obtained from a GEO expression dataset (GSE159657). We also analyzed bioinformatics functionality, potential pathways, and interaction networks related to the microRNAs associated with the differentially expressed circRNAs. A total of 253 exosomal circRNAs (184 up- and 69 down-regulated) and 182 exosomal circRNAs (94 up- and 88 down-regulated) were identified as being differentially expressed between the control group and the AMI and CAD patients, respectively. Compared with the CAD group, 231 different exosomal circRNAs (177 up- and 54 down-regulated) were identified in the AMI group. Functional analysis suggested that the parental genes of exosomal has_circ_0061776 were significantly enriched in the biological process of lysine degradation. Pathway interaction network analysis further indicated that exosomal has_circ_0061776 was associated with has-miR-133a, has-miR-214, has-miR-423, and has-miR-217 and may play a role in the pathogenesis of AMI through the MAPK signaling pathway. This study identified the differential expression and functionality of exosomal circRNAs in AMI and provided further understanding of the potential pathogenesis of an exosomal circRNA-related competing endogenous RNA (ceRNA) network in AMI.

## 1. Introduction

Acute myocardial infarction (AMI) is a common cardiovascular disease with high rates of morbidity and mortality worldwide [1]. Typically, patients with known cardiovascular disease who do not have an acute course are often referred to as having stable coronary artery disease (CAD) [2]. AMI is a serious type of coronary artery disease (CAD) and remains a major public health problem worldwide [3, 4]. The prevention of AMI in CAD patients remains an unmet clinical challenge due to the high prevalence of CAD and associated mortality [5]. Although substantial improvements have been made in terms of prognosis over the past decade via early reperfusion strategies, this has reduced in-hospital mortality to approximately 5%; however, mortality at 1-year post-AMI still remains at 9% [6]. Therefore, there is an urgent need to better clarify the pathogenesis of AMI so that we can prevent and treat this disease earlier. Although the pathogenesis and risk factors for AMI are still not fully understood, it is generally accepted that AMI is closely related to genetic and environment factors [7].

Over recent years, many studies have shown that non-coding RNAs, such as circular RNAs (circRNAs) and mRNAs, play an important role in the pathogenesis of AMI [8–10]. Since circRNAs play a significant role in cellular metabolism and pathological disease states, the characterization and quantification of circRNAs from high-throughput RNA-seq data have become an emerging research area for AMI [11]. With the application of next-generation sequencing and bioinformatics, new information relating to the role of circRNAs in AMI has gradually emerged [12, 13]. For example, Devaux et al. found circulating circRNAs could improve risk classification after AMI, thus supporting the added value of this novel biomarker in future prognostication strategies [14]. Several studies have suggested that circulating circRNAs may play a role in the development of AMI through different pathways [15–17]. CircRNAs are a special class of covalently closed non-coding RNA molecule in eukaryotes with tissue-specific and cell-specific expression patterns; these RNAs exist in a variety of tissues and also in the circulating blood [18, 19]. A recent study demonstrated the presence of circRNAs, miRNAs, and long non-coding RNAs in small extracellular vesicles called exosomes [20].

Exosomes, a group of nanosized extracellular vesicles with a typical size of 40-100 nm, contain a variety of bioactive nucleic acids, lipids, and proteins and represent a largely unknown “cell-to-cell” communication system [21]. The biologically active cargo carried by exosomes could alter the phenotype of recipient cells [22, 23]. Therefore, exosomes are increasingly becoming recognized as playing an important role in the progression and treatment of cardiac disease states, including AMI [24, 25].

In a previous study, we investigated plasma exosomal long RNAs (including circRNA, long non-coding RNAs (lncRNA), and messenger RNA (mRNA)) profiles of individuals with AMI and CAD by sequencing technology [26]. These results demonstrated that the profiles and functional analysis of the exosomal mRNAs of patients with AMI differed significantly with healthy controls and were associated with an acute inflammatory response to immune cell types. However, the circRNA profiles of plasma exosomes in AMI patients, and their functional roles, remain poorly understood. Therefore, in the present study, we investigated differential circRNA profiles of plasma exosomes between AMI and CAD patients and with healthy controls by analyzing the Gene Expression Omnibus (GEO) Dataset (GSE159657). Our aim was to lay down a foundation for further research relating to the role of exosomal circRNAs in AMI development.

## 2. Material and Methods

### 2.1. GEO Expression Dataset Retrieval

To construct a comparative profile of exosomal circRNAs in the plasma from patients with AMI and CAD, along with controls, we download the raw sequencing data from our previous reported GEO expression dataset (GSE159657) [26]. This dataset features ten AMI patients, eight CAD patients, and ten healthy individuals. In brief, plasma was separated by centrifugation and stored at -80°C. Then, we used an exoRNeasy Serum/Plasma kit (Qiagen) to extract exosomal RNA in accordance with the manufacturer's instructions. An Epi mini lncRNA-seq kit (Epibiotek) was used for library preparation in accordance with the manufacturer's instructions. Double-stranded cDNA from the first step PCR was purified with Epi DNA Clean Beads (Epibiotek). The quality of the RNA for sequencing was determined using a Bioptic Qsep100 Bioanalyzer. First strand cDNA was synthesized from mRNA using SMART technology as part of the SMART cDNA Library Construction kit (Clontech). Libraries were barcoded, pooled, and then sequenced on an Illumina Nova-Seq 6000 sequencing system. Cutadapt (v2.5) was used to trim adapters and filter for sequences; then, the remaining reads were aligned to the human Ensemble genome GRCh38 using Hisat2 aligner (v2.1.0) using the “--rna-strandness RF” parameter. The reads that mapped to Mthe genome were then calculated using Feature Counts (v1.6.3).

### 2.2. CircRNA Identification and Quantification

The pipeline “Accurate CircRNA Finder Suite (ACFS)” (https://github.com/arthuryxt/acfs) can be used to examine and pinpoint the back-splice site from RNA-seq alignments using a maximal entropy model. The expression of circRNAs can then be estimated from a second round of alignments to the inferred pseudo circular sequences [27]. ACFS is commonly used to identify each sample of circRNA via a series of key steps [27]: For the collection of unmapped reads, we used BOWTIE2 version 2.2.5 [28] as a mapping method to the respective reference genome utilizing the parameter “bowtie2.” To identify circRNAs, we used BWA mem and collated unmapped reads. Partial alignments of segments within a single read that mapped to (i) regions on the same chromosome and no more than 1 Mb away from each other, (ii) on the same strand, and (iii) in reverse order were retained as candidates supporting the head-to-tail junction. The strength of potential splicing sites supported by these candidate head-to-tail junction reads was then estimated using MaxEntScan33. The exact junction site was determined by selecting the donor and acceptor sites with the highest splicing strength score. Candidate circRNAs were reported if the head-to-tail junction was supported by at least two reads and the splicing score was greater than or equal to 10. For most of the circRNAs, there was no direct evidence for their exact sequence; in these cases, we filled in the sequence using existing exon annotation. The sequence at the 5′-end was concatenated to the 3′-end to form circular junctions. Reads that mapped to the junction (with an overhang of at least 6 nt) were counted for each candidate. All circRNAs identified from the sequencing data were annotated in the circBase database.

### 2.3. Differential Expression Analysis

We applied DESeq2 algorithm to filter the differentially expressed genes, after the significant analysis and FDR analysis under the following criteria: log2 fold change ≥ |1.0| at statistical significance of *P* < 0.05. Volcano plots and expression heatmaps were drawn using the “pheatmap” package on R.

### 2.4. Function of the Exosomal circRNAs Parental Genes

Gene ontology (GO) enrichment analysis for cellular and molecular functions regulated by the differently expressed circRNAs was performed using ClusterProfiler package [29]. Pathways regulated by the differently expressed exoLRs were then identified using Kyoto Encyclopedia of Genes and Genomes (KEGG) pathway enrichment analysis, as previously described [30] with a significance of *P* < 0.05.

### 2.5. Annotation of circRNA/miRNA Interactions

Based on previous literature [31, 32], we selected 88 cardiovascular diseases related to microRNAs (Supplementary Table S1) to predict circRNA/microRNA interactions. To identify potential microRNA binding sites, we used RNAhybrid [33] and miRanda [34] programs software. Then, the algorithms TargetScan [35], miRDB [36], and PicTar [37] to predict the biological targets of microRNAs.

### 2.6. Statistical Analysis

Significant differences between groups were identified by Student's *t*-test. *P* < 0.05 was considered statistically significant. Data were analyzed using R software (R software, version 4.0.1).

## 3. Results

### 3.1. CircRNA Expression Profiling in Plasma Exosomes among AMI, CAD Patients, and Controls

**Figure 1:**
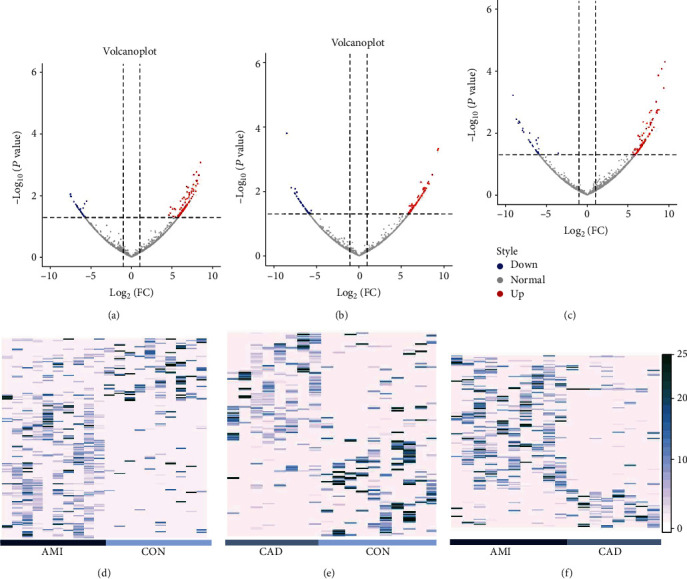
The expression patterns of exosomal circRNAs expression in circulating plasma among the AMI, the CAD, and the control group. Volcano plots of exosomal circRNAs in the comparisons of AMI versus control (a), CAD versus control (b), and AMI versus CAD (c). The heatmaps of significantly differentially expressed circRNAs in the comparisons of AMI versus control (d), CAD versus control (e), and AMI versus CAD (f).

**Figure 2:**
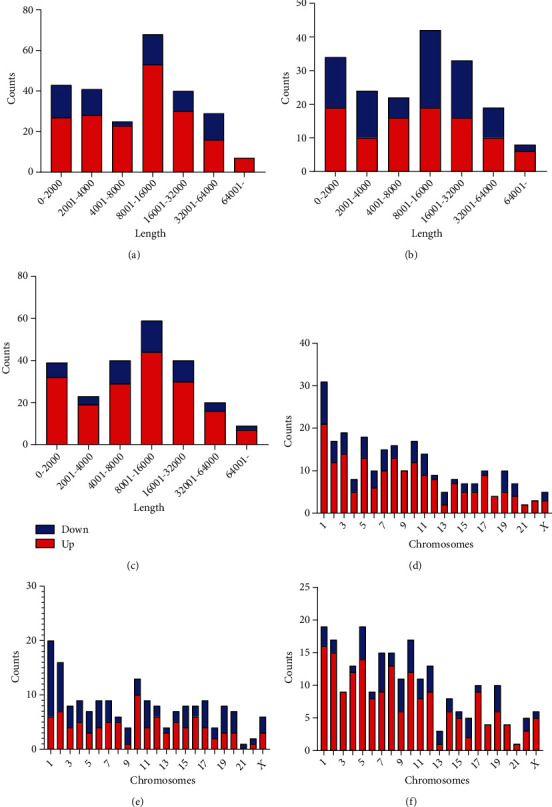
The basic characteristics of differential exosomal circRNAs in circulating plasma. The length distribution of differentially expressed circRNAs in the comparisons of AMI versus control (a), CAD versus control (b), and AMI versus CAD (c). The chromosomal distribution of differentially expressed circRNAs in the comparisons of AMI versus control (d), CAD versus control (e), and AMI versus CAD (f).

The basic characteristics of the circRNAs profiles in plasma exosomes were analyzed through GEO dataset (GSE159657). We identified a total of 14698 circRNAs. Volcano plot showed that circRNAs significantly differentially expressed from each pairwise comparison (Figures 1(a)–1(c)). In the AMI patients, based on the screening criteria of log2(fold change) ≥1 and *P* < 0.05, a total of 253 exosomal circRNAs consisting of 184 up- and 69 down-regulated (Supplementary Table S2) were screened as differentially expressed exosomal circRNAs compared with control group (Figure 1(a)) and the top 20 of these circRNAs are shown in Table 1. In the CAD patients, based on the same screening criteria, a total of 182 exosomal circRNAs consisting of 94 up- and 88 down-regulated (Supplementary Table S3) were screened as differentially expressed exosomal circRNAs compared with control group (Figure 1(b)) and the top 20 of these circRNAs are shown in Table 2. When compared with the CAD group, 231 different exosomal circRNAs (Supplementary Table S4), consisting of 177 up- and 54 down-regulated circRNAs, were identified for the AMI group (Figure 1(c)) and the top 20 differentially expressed circRNAs are shown in Table 3. The results of hierarchical cluster analysis indicated that the expression patterns of exosomal circRNA expression in circulating plasma were distinguishable among the AMI, the CAD, and the control group (Figures 1(d)–1(f)). Figures 2(a)–2(c) and 2(d)–2(f) show the lengths and chromosomal distribution of the differential circRNAs in the comparison of AMI versus control, CAD versus control, and AMI versus CAD, respectively. Most of these circRNAs were dominated by up-regulated circRNAs and were 8001-10000 bp lengths. The results suggested that the expression profiles of exosomal circRNAs in circulating plasma were different from each pairwise comparison.

### 3.2. GO Analysis of the circRNA Parental Genes

**Figure 3:**
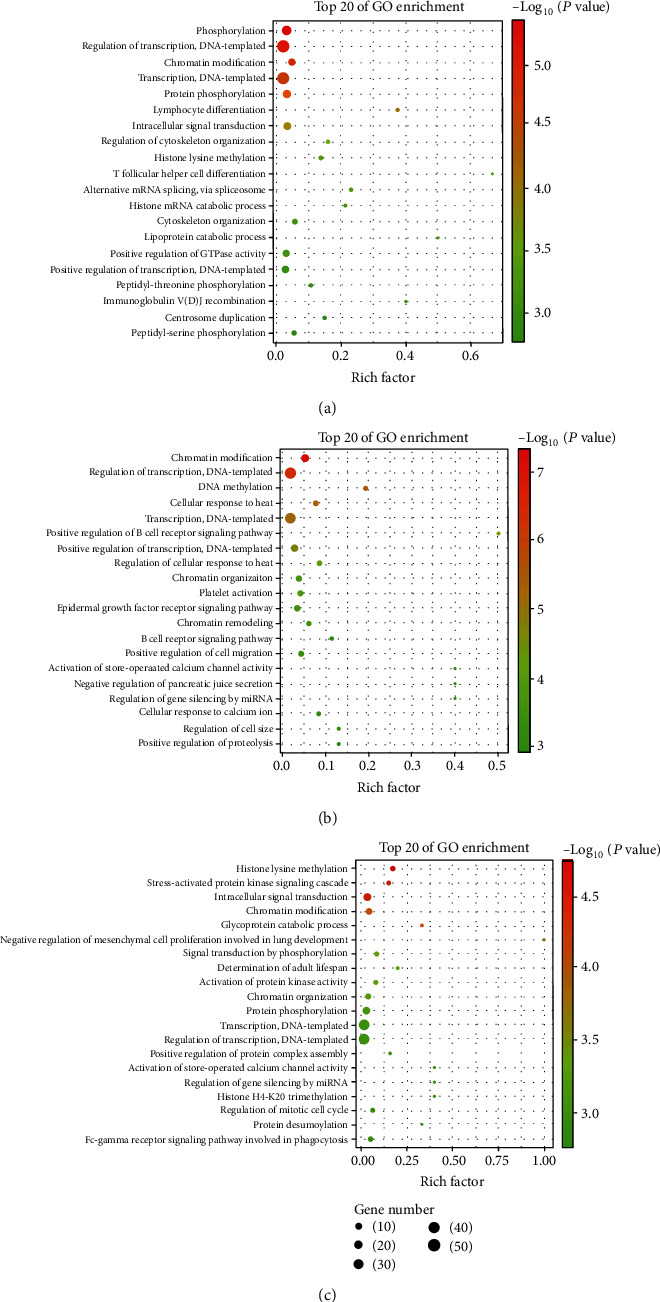
The biological process enrichment of differential circRNAs parental genes in the comparisons of AMI versus control (a), CAD versus control (b), and AMI versus CAD (c).

To investigate the functional characteristics of the differentially expressed circRNA parental genes for each pairwise comparison, we performed a GO analysis. The GO terms were measured by the rich factor, *q* value, and number of genes enriched. At the *P*-value cut-off of 0.05, the enrichment of differential circRNAs parental genes in the GO biological process was obtained. By comparison of AMI and control exosomal circRNA functional profile, biological process mainly consisting of phosphorylation associated progresses including protein, peptidyl-threonine, and peptidyl-serine phosphorylation was significantly enriched (Figure 3(a)). When compared with CAD and control, the parental genes were mainly involved in cellular response to heat, B cell receptor signaling pathway, and platelet activation (Figure 3(b)). In addition, histone lysine methylation was the most significantly enriched Go term in comparison of AMI and CAD (Figure 3(c)). Besides, stress-activated protein kinase signaling cascade and intracellular signal transduction were also significantly enriched. We found that the three pairwise comparison of functional profile analysis consistently suggested that most genes were enriched in regulation of transcript, DNA-templated, and chromatin modification (Figure 3). All parental genes involved in GO terms were listed in Supplementary Tables S5-S7 for each pairwise comparison in detail.

### 3.3. KEGG Pathway of the circRNA Parental Genes

**Figure 4:**
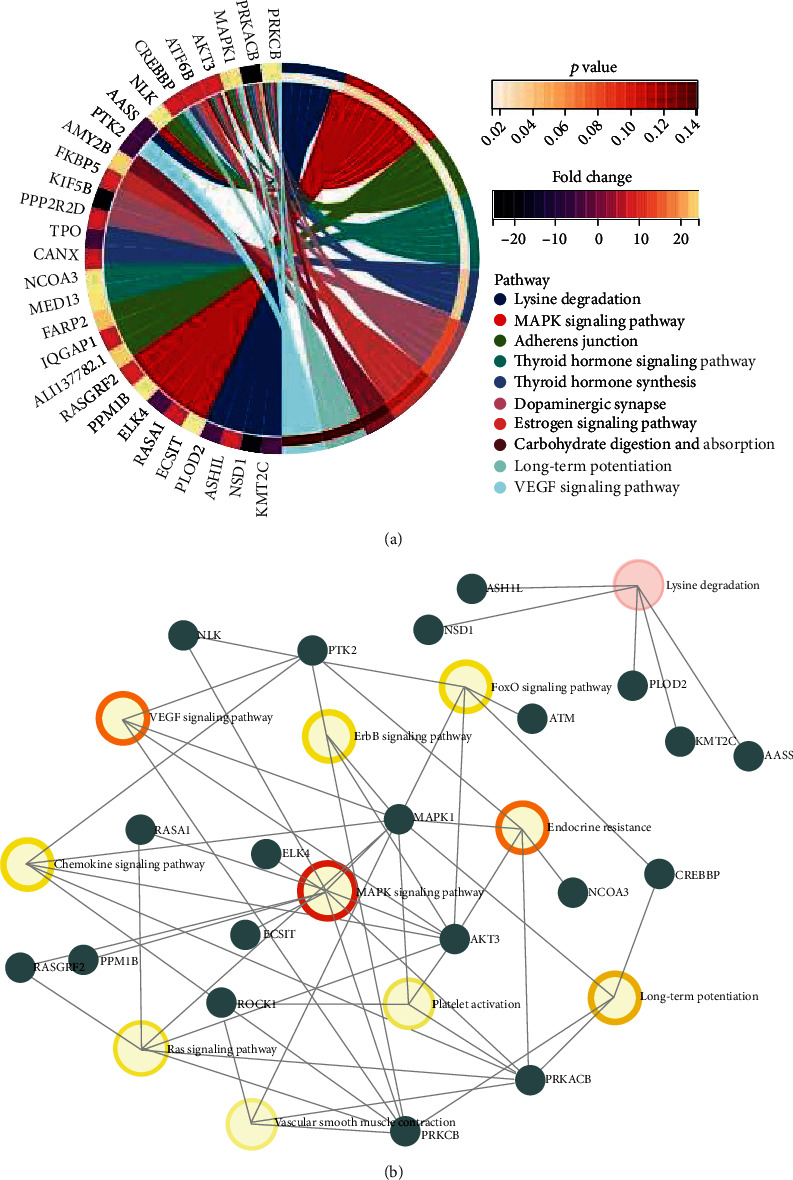
The KEGG pathway enrichment of differential circRNAs parental genes in the comparisons of AMI versus control (a) and the parental gene-pathway networks in the comparison of AMI versus control (b).

**Figure 5:**
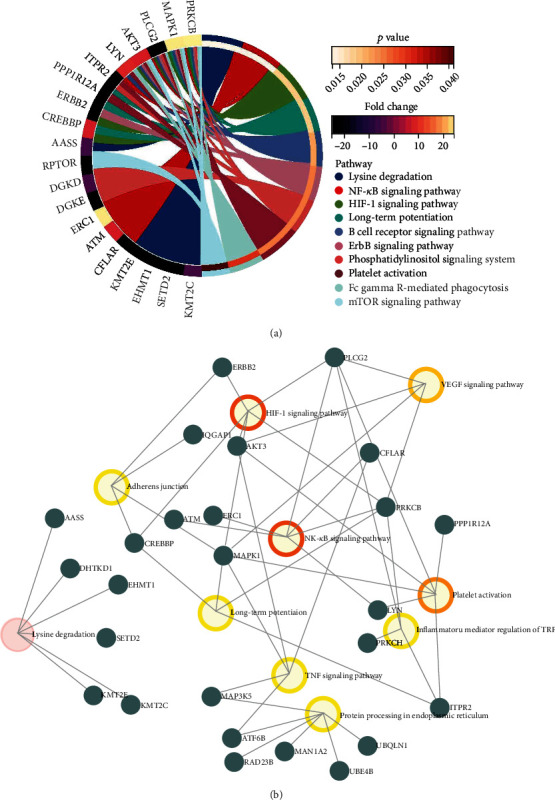
The KEGG pathway enrichment of differential circRNAs parental genes in the comparisons of CAD versus control (a) and the parental gene-pathway networks in the comparison of CAD versus control (b).

**Figure 6:**
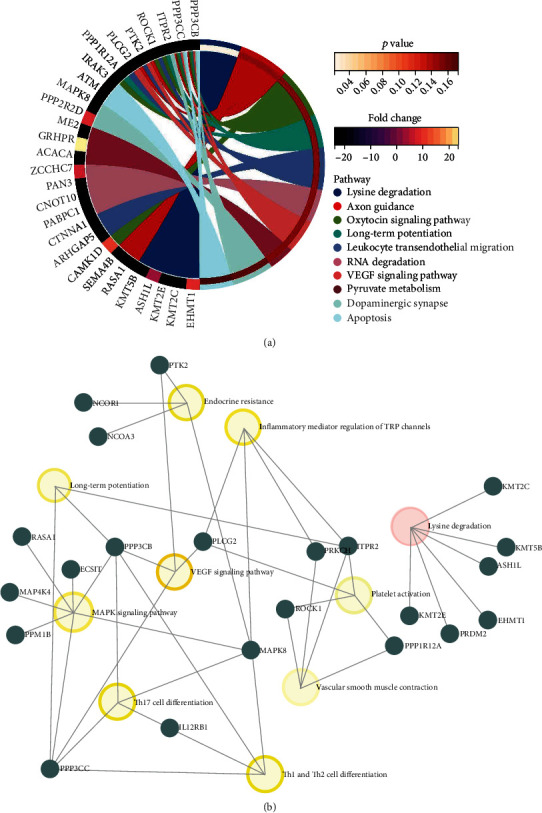
The KEGG pathway enrichment of differential circRNAs parental genes in the comparisons of AMI versus CAD (a) and the parental gene-pathway networks in the comparison of AMI versus CAD (b).

To investigate the differentially expressed circRNA parental gene-related pathways, we firstly performed KEGG pathway enrichment analysis for each pairwise comparison. By comparison of AMI and control, the parental genes were mainly involved in MAPK signaling pathway, adheres junction and thyroid hormone associated pathway (Figure 4(a)). When compared with CAD and control, NF-kappa B signaling pathway and HIF-1 signaling pathway were significantly enriched. In addition, axon guidance, oxytocin signaling pathway, long-term potentiation, leukocyte trans endothelial migration, RNA degradation, and VEGF signaling pathway were the mainly significantly enriched Go term in comparison of AMI and CAD (Figure 3(c)). The three pairwise comparison of KEGG pathway enrichment analysis consistently suggested that the most significantly and the highest rich factor pathway was lysine degradation (Figures 4(a), 5(a), and 6(a)). All parental genes involved in KEGG pathway terms were listed in Supplementary Tables S8-S10 for each pairwise comparison in detail. Furthermore, to investigate the critical pathways in the three pairwise comparisons, parental gene-pathway networks were constructed according to the overlap of common differentially expressed circRNA parental genes. In Figures 4(b), 5(b), and 6(b), the more intensive color indicated the higher betweenness centrality of hubs. The most exchange pathway in the network was considered hub pathway which might be a key pathway. The results showed that the networks majorly depended on the existence of MAPK signaling pathway, NF-kappa B signaling pathway, and VEGF signaling pathway in comparison of AMI versus control (Figure 4(b)), CAD versus control (Figure 5(b)), and AMI versus CAD (Figure 6(b)), respectively.

### 3.4. CircRNA-miRNA Interactions Predictions

**Figure 7:**
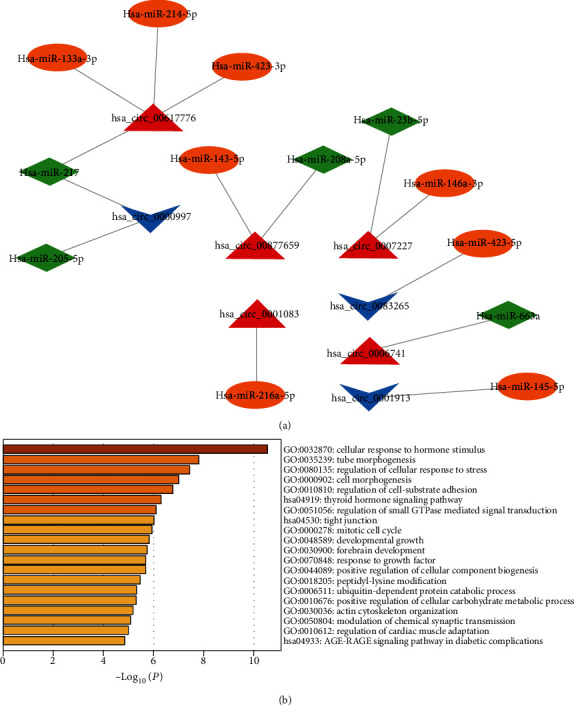
The interactions networks of cardiovascular diseases related microRNAs involving the differentially expressed circRNAs in AMI group compared with the control group (a) and the functional enrichment of the targets in the interactions networks in the comparison of AMI versus control (b).

To investigate the association between differentially expressed circRNA and atherosclerosis-associated microRNAs, we chose the top 20 differentially expressed circRNAs to predicted circRNA-miRNA interactions. In the comparison of AMI versus control, five up-regulated and three down-regulated circRNAs were found interaction with 13 cardiovascular diseases related microRNAs, including 8 cardiovascular protective microRNA and 5 cardiovascular pathogenic microRNA (Figure 7(a)). Among them, has_circ_0061776 was predicted to act as a sponge for 3 cardiovascular protective microRNA (has-miR-133a, has-miR-214, and has-miR-423) and 1 cardiovascular pathogenic microRNA (has-miR-217). Thus, has_circ_0061776 might be involved in AMI. Through the algorithms TargetScan, miRDB, and PicTar, we obtained 247 potential targets of these microRNAs (Supplementary Table S11). The functional enrichment of the potential targets of these thirteen microRNAs shows that cellar response to hormone stimulus was the most significant biological progress and the most significant pathway was thyroid hormone signaling pathway (Figure 7(b)). The result was also consistent with KEGG pathway enrichment of circRNA parental genes (Figure 4). Therefore, it indicated that the circRNAs might regulate hormone-associated biological progress through microRNA which would play a role in AMI.

**Figure 8:**
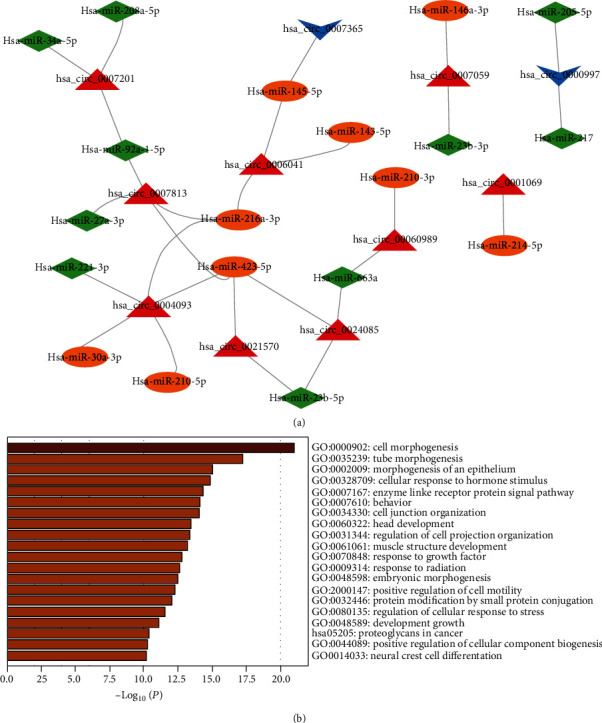
The interactions networks of cardiovascular diseases related microRNAs involving the differentially expressed circRNAs in CAD group compared with the control group (a) and the functional enrichment of the targets in the interactions networks in the comparison of CAD versus control (b).

In the comparison of CAD versus control, hsa-miR-216a and hsa-miR-423 could be regulated by 3 differentially expressed circRNAs at the same time (Figure 8(a)). Among them, has_circ_0006041 was predicted to act as a sponge for 3 cardiovascular protective microRNA (has-miR-145, has-miR-143, and has-miR-216), indicating that has_circ_0006041 might be a cardiovascular pathogenic circRNA (Figure 8(a)). However, has_circ_0007201 was predicted to act as a sponge for 3 cardiovascular pathogenic microRNA (has-miR-34a, has-miR-208a, and has-miR-92a) which might be beneficial for cardiovascular. Then, we predicted 666 potential targets (Supplementary Table S12) of the microRNAs in Figure 8(a) and performed functional enrichment analysis of these targets. The results showed that cell morphogenesis was the most significant biological progress and the most significant pathway was proteoglycans in cancer (Figure 8(b)). In addition, tube morphogenesis, morphogenesis of an epithelium, cellular response to hormone stimulus, and enzyme-linked receptor protein signaling pathway were also involved (Figure 8(b)).

**Figure 9:**
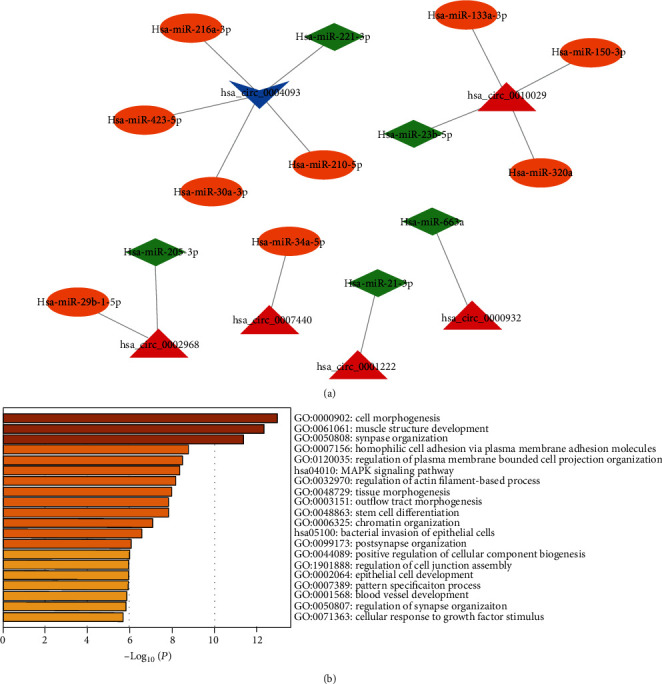
The interactions networks of cardiovascular diseases related microRNAs involving the differentially expressed circRNAs in AMI group compared with the CAD group (a) and the functional enrichment of the targets in the interactions networks in the comparison of AMI versus CAD (b).

While in the comparison of AMI versus CAD, the network showed that the down-regulated has_circ_0004093 and the up-regulated has_circ_0007201 involved with 3 cardiovascular protective microRNA and 1 cardiovascular pathogenic microRNA might play opposite effects on coronary artery disease, respectively (Figure 9(a)). 260 potential targets (Supplementary Table S13) of the microRNAs in the network were predicted to investigate the function of these circRNAs. The results showed that cell morphogenesis was still the most significant biological progress (Figure 9(b)). Besides, the targets of these circRNAs were also involved in muscle structure development, synapse organization, homophilic cell adhesion via plasma membrane adhesion molecules, and regulation of plasma membrane bounded cell projection organization (Figure 9(b)). For KEGG pathway analysis, MAPK signaling pathway and bacterial invasion of epithelial cells were significantly enriched (Figure 9(b)).

## 4. Discussion

Based on our raw sequencing data, we identified a total of 14698 circRNAs. In addition, 253 exosomal circRNAs (184 up- and 69 down-regulated) and 182 exosomal circRNAs (94 up- and 88 down-regulated) were identified as differentially expressed exosomal circRNAs compared between the control group and the AMI and CAD patients, respectively. The expression patterns of exosomal circRNAs in AMI and CAD patients were different from that in controls. In addition, 231 different exosomal circRNAs (177 up- and 54 down-regulated) were identified when comparing between the AMI and CAD groups. Exosomal has_circ_0061776, has_circ_0004093, has_circ_0007201, has_circ_0006041, and has_circ_0007201 may be involved in the initiation and progression of AMI and CAD and appear to be related to the MAPK signaling pathway. According to our analysis, up-regulated circRNAs were most dominant. This indicated that an impairment in the coronary circulation might increase the production of circulating exosomal circRNAs.

In addition, our study indicated that the expression patterns of exosomal circRNAs were significantly different when compared between the AMI, CAD, and control populations. Exosomal circRNAs have become a key hotspot in the field of RNA research, especially with the rapid development and extensive application of exosome extraction and RNA sequencing technology. Accumulating evidence now indicates that exosomal microRNAs and long non-coding RNAs play a pivotal role in the pathology of atherosclerotic cardiovascular disease including CAD and AMI [38–40]. Li et al. and Wang et al. both reported that the expression patterns of exosomal circRNAs were significantly different in AMI when compared to healthy controls and play an important role in the pathological process of AMI [41, 42].

Although the biogenesis of circRNAs has been investigated extensively, the expression of circRNAs in cardiovascular diseases is still poorly understood [43]. Since exosomes are considered key components of intercellular communication [44], unravelling the profiles of exosomal circRNAs might be useful in understanding the occurrence and development of cardiovascular diseases. Therefore, this study focused on the expression profiles of circulating exosomal circRNAs of AMI and CAD patients in order to provide a basis for further research on the function of exosomal circRNAs during the development of AMI.

Based on hierarchical cluster analysis, although circRNA expression in circulating exosomes was distinguishable between each group, exosomal circRNAs were low in abundance and individual differences could have affected the findings of the present study. In the current circRNA research, the reliability of circRNA characterization and quantification can vary considerably depending on multiple factors such as detection tools, sequencing technology, and sample type [45, 46]. We found that compared to controls, exosomal has_circ_0061776, has_circ_0004093, and has_circ_0007201 were significantly and differentially expressed in AMI patients, along with exosomal has_circ_0006041 and has_circ_0007201 in CAD patients, respectively. These findings suggest that exosomal circRNAs might contribute to the pathogenesis of AMI or CAD [18, 47–49]. However, larger cohort studies were still required to validate the reliability of exosomal circRNAs as clinical biomarkers. Further studies are also required to understand the mechanisms underlying the secretion of exosomal circRNAs.

In addition, we investigated the potential biological functions and signaling pathways of the parental genes for these differential circRNAs. According to GO and KEGG pathway analyses, two biological processes, chromatin modification and the regulation of transcription, DNA-templated, and one KEGG pathway, lysine degradation, were found to be significantly enriched in each pairwise comparison. Recent studies have broadened our understanding of the development of the cardiovascular system at the chromatin level, including the modification of chromatin [50–52]. In addition, chromatin modification induced by the histone methyltransferase was associated with pathological cardiac hypertrophy and the clinical treatment of heart failure [53]. In terms of metabolic process, KEGG pathway analyses showed that lysine degradation was associated with the development of coronary artery lesions and is therefore an important process in AMI. Lysine plays crucial functions in the promotion of human physiologic development and fatty acids oxidation. An early study of the relationship between dietary lysine and arterial calcification showed that lysine supplementation was able to ameliorate arterial calcification by inhibiting apoptosis in the vascular smooth muscle cells [54]. This metabolic alteration was also reported to contribute to the inflammatory process [55, 56]. Both inflammation and apoptosis are closely related to the process of AMI. However, the progression of AMI under the influence of lysine requires further study. Furthermore, we further extended our pathway analysis to a constructed network in the present study. For AMI versus control and AMI versus CAD, the exchange between these pathways mainly depended on the MAPK signaling pathway which is well known to be associated with cell proliferation, differentiation, migration, senescence, and apoptosis [57, 58]. In addition, the MAPK signaling pathway and its related signaling networks could contribute to the promotion of cardiovascular diseases involving oxidative stress and inflammation [59, 60]. In addition, circRNAs are also considered to be a novel type of non-coding RNAs in cardiovascular metabolic inflammation [61]. These results suggested that exosomal circRNAs may play a role in the occurrence of AMI by regulating the MAPK signaling pathway.

To investigate the association between differentially expressed circRNAs and atherosclerosis-associated microRNAs, the top 20 differentially expressed circRNAs were used to predict circRNA-miRNA interactions using the circular RNA interactome. We found that hsa-miR-663a, hsa-miR-423, and hsa-miR-23b were able to pair with most of the top 20 differentially expressed circRNAs in the three pairwise comparisons. Currently, linkage evidence suggests that circRNAs are able to regulate parental gene expression through diverse mechanisms, such as miRNA sponges, mRNA traps, cis-or trans-acting modulation, and for complexes with proteins to perform biological functions [62–64]. Since circRNAs have more miRNA binding sites as compared to linear miRNA sponges [65, 66], the predominant function of miRNA sponges could be to reduce the activity of target miRNAs and increase the complexity of the competing endogenous RNA (ceRNA) network [67, 68]. Hsa-miR-423 was previously reported to inhibit cardiomyocyte apoptosis [69] while hsa-miR-663a has been shown to increase monocyte adhesion and endothelial cell inflammation [70, 71]. In addition, hsa-miR-23b can suppress endothelial cell proliferation [72]. These two microRNAs, hsa-miR-663a and hsa-miR-23b, have also been considered proatherogenic vascular mechano-microRNAs [32]. Therefore, the pathogenic mechanisms of circulating exosomal circRNAs in the development of atherosclerosis might be associated with the effects of miRNA-mediated processes. However, further investigation on the mechanisms of circRNA-miRNA-target gene interactions in coronary heart atherosclerotic diseases (such as CAD and AMI) is critical in the future.

Our future research will target the role of the MAPK signaling pathway in the initiation and progression of AMI and CAD. In addition, the mechanisms associated with differentially expressed circRNA functions as miRNA sponges related to atherosclerosis will also be addressed in our future research.

Although we discovered a network of potential roles for exosomal circular RNAs in the occurrence of AMI or CAD by multiple bioinformatics approaches, several limitations of this study should be acknowledged. First, the differentially expressed exosomal circRNAs need to be further validated in a larger cohort due to our small sample size. Second, this study dataset could have resulted in some variability due to individual differences in clinical disease, such as age. Third, our results were mainly derived from bioinformatics analyses and predictions; thus, the proposed mechanisms of circRNAs need to be confirmed by further research involving both cell and animal models.

## 5. Conclusion

Our analyses demonstrated that differentially expressed circRNAs in AMI and CAD patients might be associated with the development of atherosclerosis and suggested that exosomal circRNAs (has_circ_0061776, has_circ_0004093, has_circ_0007201, has_circ_0006041, and has_circ_0007201) may play differential roles during AMI and CAD by adsorption to has-miR-133a, has-miR-214, and has-miR-423, has-miR-217, has-miR-145, has-miR-143, has-miR-216, has-miR-34a, has-miR-208a, and has-miR-92a. The expression profiles and potential functions of the aberrantly expressed exosomal circRNAs involved in the pathogenesis of AMI and CAD may be related to the MAPK pathway. Our study provides a basis for future research relating to the pathogenic mechanisms of circulating exosomal circRNAs in AMI and CAD.

## Figures and Tables

**Table 1:** The top 20 differentially expressed exosomal circRNAs in AMI group compared with the control group.

circRNA ID	circBase name	log2(fold change)	*P* value	Regulation	Parental genes
chr1_21111383_21002713_-108670-EIF4G3	hsa_circ_0007227	23.86	7.02E-16	Up	EIF4G3
chr1_31007099_30992390_-14709-PUM1	hsa_circ_0006636	-23.58	1.57E-15	Down	PUM1
chr13_100273346_100257595_+15751-PCCA	hsa_circ_0000500	23.63	1.34E-15	Up	PCCA
chr19_5654456_5653115_+1341-SAFB	hsa_circ_0000881	24.15	3.19E-16	Up	SAFB
chr21_41257326_41237513_+19813-BACE2	hsa_circ_0061776	23.94	5.75E-16	Up	BACE2
chr2_189818180_189791790_+26390-PMS1	hsa_circ_0001083	23.79	8.53E-16	Up	PMS1
chr2_44218537_44209210_+9327-PPM1B	hsa_circ_0009062	23.54	1.72E-15	Up	PPM1B
chr2_45562756_45546732_-16024-SRBD1	hsa_circ_0000997	-24.97	3.06E-17	Down	SRBD1
chr7_27800090_27785163_+14927-TAX1BP1	NA	23.92	6.08E-16	Up	TAX1BP1
chr7_22984045_22976210_-7835-FAM126A	hsa_circ_0001971	23.74	9.93E-16	Up	FAM126A
chr7_50327757_50319048_+8709-IKZF1	hsa_circ_0001708	-23.96	5.31E-16	Down	IKZF1
chr12_111418877_111418119_+758-SH2B3	hsa_circ_0006741	23.57	1.60E-15	Up	SH2B3
chr12_28391411_28255581_+135830-CCDC91	NA	23.57	1.60E-15	Up	CCDC91
chr9_97350555_97347314_+3241-CCDC180	hsa_circ_0087659	24.32	1.98E-16	Up	CCDC180
chrX_19695741_19683823_-11918-SH3KBP1	hsa_circ_0001913	-24.10	3.60E-16	Down	SH3KBP1
chr8_1909470_1876571_+32899-ARHGEF10	hsa_circ_0083265	-24.30	2.10E-16	Down	ARHGEF10
chr19_11514221_11513056_-1165-ECSIT	hsa_circ_0006471	24.06	4.12E-16	Up	ECSIT
chr5_132893118_132892164_-954-AFF4	hsa_circ_0001529	23.57	1.58E-15	Up	AFF4
chr5_177050936_177041126_+9810-ZNF346	hsa_circ_0001559	23.48	2.04E-15	Up	ZNF346
chr17_28172618_28163543_+9075-NLK	hsa_circ_0003638	23.69	1.13E-15	Up	NLK

NA: novel circRNA not annoted by circBase.

**Table 2:** The top 20 differentially expressed exosomal circRNAs in CAD group compared with the control group.

circRNA ID	circBase name	log2(fold change)	*P* value	Regulation	Parental genes
chr1_36173478_36170971_+2507-MAP7D1	hsa_circ_0004093	24.42	2.23E-16	Up	MAP7D1
chr20_35732135_35716740_-15395-RBM39	NA	24.38	2.51E-16	Up	RBM39
chr11_32935435_32927157_+8278-QSER1	hsa_circ_0021570	24.15	4.77E-16	Up	QSER1
chr10_12120267_12081472_+38795-DHTKD1	hsa_circ_0007813	23.98	7.56E-16	Up	DHTKD1
chr7_66286709_66240325_+46384-TPST1	hsa_circ_0006041	23.79	1.29E-15	Up	TPST1
chr11_96093517_96091892_-1625-MAML2	hsa_circ_0024085	23.63	2.00E-15	Up	MAML2
chr16_31723353_31722626_+727-ZNF720	hsa_circ_0007059	23.44	3.35E-15	Up	ZNF720
chr20_58673711_58667490_+6221-STX16	hsa_circ_0060989	23.25	5.48E-15	Up	STX16
chr15_90492711_90486954_+5757-IQGAP1	hsa_circ_0007201	22.82	1.76E-14	Up	IQGAP1
chr2_135639122_135631718_+7404-R3HDM1	hsa_circ_0001069	22.79	1.85E-14	Up	R3HDM1
chr17_56862251_56848696_+13555-DGKE	NA	22.75	2.07E-14	Up	DGKE
chr11_77693611_77683710_-9901-RSF1	hsa_circ_0000344	-22.89	2.03E-14	Down	RSF1
chr21_39212707_39206108_-6599-BRWD1	NA	-23.11	1.14E-14	Down	BRWD1
chr6_75647801_75621532_+26269-SENP6	hsa_circ_0001612	-23.11	1.12E-14	Down	SENP6
chr1_31007099_30992390_-14709-PUM1	hsa_circ_0006636	-23.15	1.04E-14	Down	PUM1
chr20_38066256_38057532_+8724-RPRD1B	hsa_circ_0007365	-23.16	9.94E-15	Down	RPRD1B
chr15_92967513_92945821_+21692-CHD2	NA	-23.21	8.78E-15	Down	CHD2
chr19_10177367_10163326_-14041-DNMT1	NA	-23.69	2.39E-15	Down	DNMT1
chr2_1436384_1413264_+23120-TPO	NA	-23.83	1.67E-15	Down	TPO
chr2_45562756_45546732_-16024-SRBD1	hsa_circ_0000997	-24.55	2.31E-16	Down	SRBD1

NA: novel circRNA not annoted by circBase.

**Table 3:** The top 20 differentially expressed exosomal circRNAs in AMI group compared with the CAD group.

circRNA ID	circBase name	log2(fold change)	*P* value	Regulation	Parental genes
chr1_36173478_36170971_+2507-MAP7D1	hsa_circ_0004093	24.42	2.23E-16	Up	MAP7D1
chr20_35732135_35716740_-15395-RBM39	NA	24.38	2.51E-16	Up	RBM39
chr11_32935435_32927157_+8278-QSER1	hsa_circ_0021570	24.15	4.77E-16	Up	QSER1
chr10_12120267_12081472_+38795-DHTKD1	hsa_circ_0007813	23.98	7.56E-16	Up	DHTKD1
chr7_66286709_66240325_+46384-TPST1	hsa_circ_0006041	23.79	1.29E-15	Up	TPST1
chr11_96093517_96091892_-1625-MAML2	hsa_circ_0024085	23.63	2.00E-15	Up	MAML2
chr16_31723353_31722626_+727-ZNF720	hsa_circ_0007059	23.44	3.35E-15	Up	ZNF720
chr20_58673711_58667490_+6221-STX16	hsa_circ_0060989	23.25	5.48E-15	Up	STX16
chr15_90492711_90486954_+5757-IQGAP1	hsa_circ_0007201	22.82	1.76E-14	Up	IQGAP1
chr2_135639122_135631718_+7404-R3HDM1	hsa_circ_0001069	22.79	1.85E-14	Up	R3HDM1
chr17_56862251_56848696_+13555-DGKE	NA	22.75	2.07E-14	Up	DGKE
chr11_77693611_77683710_-9901-RSF1	hsa_circ_0000344	-22.89	2.03E-14	Down	RSF1
chr21_39212707_39206108_-6599-BRWD1	NA	-23.11	1.14E-14	Down	BRWD1
chr6_75647801_75621532_+26269-SENP6	hsa_circ_0001612	-23.11	1.12E-14	Down	SENP6
chr1_31007099_30992390_-14709-PUM1	hsa_circ_0006636	-23.15	1.04E-14	Down	PUM1
chr20_38066256_38057532_+8724-RPRD1B	hsa_circ_0007365	-23.16	9.94E-15	Down	RPRD1B
chr15_92967513_92945821_+21692-CHD2	NA	-23.21	8.78E-15	Down	CHD2
chr19_10177367_10163326_-14041-DNMT1	NA	-23.69	2.39E-15	Down	DNMT1
chr2_1436384_1413264_+23120-TPO	NA	-23.83	1.67E-15	Down	TPO
chr2_45562756_45546732_-16024-SRBD1	hsa_circ_0000997	-24.55	2.31E-16	Down	SRBD1

NA: novel circRNA not annoted by circBase.

## Data Availability

The raw data supporting the conclusions of this article are available in the Gene Expression Omnibus (GSE159657) (https://www.ncbi.nlm.nih.gov/geo/query/acc.cgi?acc= GSE159657).

## Abbreviations

circRNAs: Circular RNAs
AMI: Acute myocardial infarction
CAD: Coronary artery disease
GEO: Gene expression omnibus
GO: Gene ontology
KEGG: Kyoto encyclopedia of genes and genomes
MREs: miRNA response elements
FC: Fold change
ceRNA: Competing endogenous RNA.

## Supplementary Materials

Supplementary 1 Supplementary Table S1: List of cardiovascular diseases related microRNAs.

Supplementary 2 Supplementary Table S2: Differentially expressed exosomal circRNAs based on the screening criteria of fold change ≥1 and *P* < 0.05 in comparison of AMI and control.

Supplementary 3 Supplementary Table S3: Differentially expressed exosomal circRNAs based on the screening criteria of fold change ≥1 and *P* < 0.05 in comparison of CAD and control.

Supplementary 4 Supplementary Table S4: Differentially expressed exosomal circRNAs based on the screening criteria of fold change ≥1 and *P* < 0.05 in comparison of AMI and CAD.

Supplementary 5 Supplementary Table S5: Biological process enrichment analyses of the differentially expressed exosomal circRNAs in comparison of AMI and control.

Supplementary 6 Supplementary Table S6: Biological process enrichment analyses of the differentially expressed exosomal circRNAs in comparison of CAD and control.

Supplementary 7 Supplementary Table S7: Biological process enrichment analyses of the differentially expressed exosomal circRNAs in comparison of AMI and CAD.

Supplementary 8 Supplementary Table S8: Pathway enrichment analyses of the differentially expressed exosomal circRNAs in comparison of AMI and control.

Supplementary 9 Supplementary Table S9: Pathway enrichment analyses of the differentially expressed exosomal circRNAs in comparison of CAD and control.

Supplementary 10 Supplementary Table S10: Pathway enrichment analyses of the differentially expressed exosomal circRNAs in comparison of AMI and CAD.

Supplementary 11 Supplementary Table S11: Cardiovascular disease-associated microRNAs which interacted with differentially expressed exosomal circRNAs and their potential targets in comparison of AMI and control.

Supplementary 12 Supplementary Table S12: Cardiovascular disease-associated microRNAs which interacted with differentially expressed exosomal circRNAs and their potential targets in comparison of CAD and control.

Supplementary 13 Supplementary Table S13: Cardiovascular disease-associated microRNAs which interacted with differentially expressed exosomal circRNAs and their potential targets in comparison of AMI and CAD.
