# Supplementary material for: Expression Profiles and Functional Analysis of Plasma Exosomal Circular RNAs in Acute Myocardial Infarction
Source: Biomed Res Int. 2022 Oct 1;2022:3458227. doi: 10.1155/2022/3458227 (PMC9547997; doi:10.1155/2022/3458227)
Supplement: Supplementary 1 — Supplementary Table S1: List of cardiovascular diseases related microRNAs. [file 3458227.f1.docx]

Supplementary Table S1 List of cardiovascular diseases related microRNAs.

| MicroRNA | Cardiovascular Function | effect |
| --- | --- | --- |
| miR-499 | Sarcomeric genes regulation | protective |
| miR-455 | Alleviates cardiomyocyte fibrosis | protective |
| miR-423 | Alleviates cardiomyocyte apoptosis | protective |
| miR-320 | Alleviates angiogenesis in diabetes | protective |
| miR-30c | Alleviates lipid synthesis | protective |
| miR-30a | Autophagy regulation | protective |
| miR-29b | Alleviates cardiomyocyte fibrosis | protective |
| miR-223 | Alleviates macrophage activation | protective |
| miR-214 | Enhances endothelial cell function | protective |
| miR-210/216 | Inhibits macrophage infiltration | protective |
| miR-19a | Inhibits endothelial cell proliferation | protective |
| miR-195 | Alleviates inflammation | protective |
| miR-181 | Alleviates inflammation | protective |
| miR-150 | Vascular wall homeostasis | protective |
| miR-146a | Alleviates endothelial cellangiogenesis | protective |
| miR-146 | Alleviates inflammation | protective |
| miR-143/145 | Alleviates plaque formation | protective |
| miR-133 | Alleviates cardiomyocyte hypertrophy | protective |
| miR-126a | Alleviates inflammation | protective |
| miR-10a | Alleviates endothelial cell inflammation | protective |
| miR-101 | Inhibits endothelial cell proliferation | protective |
| miR-1 | Alleviates cardiomyocyte hypertrophy and oxidative stress | protective |
| miR- 467b | Alleviates lipid synthesis and inflammation | protective |
| miR-92a | Proatherogenic and exacerbates inflammation | pathogenic |
| miR-712 | Exacerbates endothelial cell inflammation | pathogenic |
| miR-663 | Exacerbates endothelial cell inflammation | pathogenic |
| miR-34a | Exacerbates cardiomyocyte oxidative stress | pathogenic |
| miR-342-5 | Exacerbates endothelial cell inflammation | pathogenic |
| miR-33 | High-density lipoprotein regulation | pathogenic |
| miR-28-3p | Exacerbates cardiomyocyte oxidative stress | pathogenic |
| miR-27a | Exacerbates cardiomyocyte oxidative stress | pathogenic |
| miR-23b | Inhibits endothelial cell proliferation | pathogenic |
| miR-221/222 | Exacerbates vascular smooth muscle calcification | pathogenic |
| miR-217 | Exacerbates cardiomyocyte hypertrophy | pathogenic |
| miR-21 | Exacerbates cardiomyocyte hypertrophy | pathogenic |
| miR-208a/b | Exacerbates cardiomyocyte fibrosis | pathogenic |
| miR-205 | Exacerbates endothelial cell inflammation | pathogenic |
| miR-155 | Alleviates nitric oxide synthase | pathogenic |
| miR-130 | Stimulates endothelial cellangiogenesis | pathogenic |
| miR-126 | Stimulates endothelial cellangiogenesis | pathogenic |
