# Supplementary material for: Expression Profiles and Functional Analysis of Plasma Exosomal Circular RNAs in Acute Myocardial Infarction
Source: Biomed Res Int. 2022 Oct 1;2022:3458227. doi: 10.1155/2022/3458227 (PMC9547997; doi:10.1155/2022/3458227)
Supplement: Supplementary 2 — Supplementary Table S2: Differentially expressed exosomal circRNAs based on the screening criteria of fold change ≥1 and P < 0.05 in comparison of AMI and control. [file 3458227.f2.docx]

Supplementary Table S2 Differentially expressed exosomal circRNAs based on the screening criteria of fold change ≥ 1 and P < 0.05 in comparison of AMI and control.

| circRNA ID | log2FC | P value | FDR | Style | CHROM | GeneName |
| --- | --- | --- | --- | --- | --- | --- |
| chr1_10105744_10095461_+10283-UBE4B | -5.91174 | 0.045929 | 0.210891 | down | chr1 | UBE4B |
| chr1_103575540_103565435_+10105-AMY2B | 21.96657 | 1.13E-13 | 1.89E-12 | up | chr1 | AMY2B |
| chr1_108161293_108148279_-13014-SLC25A24 | 5.17633 | 0.047575 | 0.213788 | up | chr1 | SLC25A24 |
| chr1_108185954_108154983_-30971-SLC25A24 | -7.58887 | 0.010307 | 0.095449 | down | chr1 | SLC25A24 |
| chr1_151639119_151638888_+231-SNX27 | -21.7668 | 1.91E-13 | 2.88E-12 | down | chr1 | SNX27 |
| chr1_155459898_155415744_-44154-ASH1L | 6.347545 | 0.0319 | 0.183191 | up | chr1 | ASH1L |
| chr1_155521618_155521100_-518-ASH1L | 6.343943 | 0.031998 | 0.183191 | up | chr1 | ASH1L |
| chr1_186419077_186406083_+12994-C1orf27 | 22.15276 | 7.00E-14 | 1.27E-12 | up | chr1 | C1orf27 |
| chr1_204114605_204112915_+1690-SOX13 | 6.496739 | 0.027557 | 0.17344 | up | chr1 | SOX13 |
| chr1_205620838_205618957_-1881-ELK4 | -6.08122 | 0.040009 | 0.195238 | down | chr1 | ELK4 |
| chr1_20942330_20904883_-37447-EIF4G3 | -6.17996 | 0.036869 | 0.192364 | down | chr1 | EIF4G3 |
| chr1_21111383_21002713_-108670-EIF4G3 | 23.86475 | 7.02E-16 | 7.73E-14 | up | chr1 | EIF4G3 |
| chr1_211793190_211778918_-14272-LPGAT1 | 7.14852 | 0.009326 | 0.087099 | up | chr1 | LPGAT1 |
| chr1_213129889_213117317_+12572-RPS6KC1 | 6.666567 | 0.024212 | 0.167803 | up | chr1 | RPS6KC1 |
| chr1_224974153_224952670_+21483-DNAH14 | 6.837598 | 0.004963 | 0.053099 | up | chr1 | DNAH14 |
| chr1_235833667_235830226_-3441-LYST | 5.84326 | 0.0483 | 0.214512 | up | chr1 | LYST |
| chr1_243344331_243304713_+39618-na | -6.35155 | 0.026746 | 0.17344 | down | chr1 | na |
| chr1_243843282_243545510_-297772-AKT3 | 6.63985 | 0.016349 | 0.133382 | up | chr1 | AKT3 |
| chr1_245929937_245915530_-14407-SMYD3 | 22.91127 | 9.46E-15 | 2.48E-13 | up | chr1 | SMYD3 |
| chr1_246591941_246591512_+429-CNST | -7.62177 | 0.008873 | 0.084837 | down | chr1 | CNST |
| chr1_28697084_28683583_+13501-GMEB1 | 6.222286 | 0.035448 | 0.189692 | up | chr1 | GMEB1 |
| chr1_31007099_30992390_-14709-PUM1 | -23.5755 | 1.57E-15 | 9.82E-14 | down | chr1 | PUM1 |
| chr1_31007102_30992390_-14712-PUM1 | 22.68052 | 1.75E-14 | 3.77E-13 | up | chr1 | PUM1 |
| chr1_33295305_33294937_+368-ZNF362 | 8.215801 | 0.004011 | 0.044204 | up | chr1 | ZNF362 |
| chr1_47282459_47280241_-2218-STIL | 7.94733 | 0.0072 | 0.072126 | up | chr1 | STIL |
| chr1_58536741_58527261_-9480-OMA1 | 6.431292 | 0.029702 | 0.178316 | up | chr1 | OMA1 |
| chr1_77714783_77711747_-3036-USP33 | 5.860467 | 0.047641 | 0.213788 | up | chr1 | USP33 |
| chr1_77815231_77801331_+13900-MIGA1 | -6.78538 | 0.021859 | 0.161651 | down | chr1 | MIGA1 |
| chr1_84197824_84179177_+18647-PRKACB | -22.8207 | 1.21E-14 | 2.88E-13 | down | chr1 | PRKACB |
| chr1_88786213_88740988_+45225-PKN2 | 22.62963 | 2.00E-14 | 4.09E-13 | up | chr1 | PKN2 |
| chr1_93216746_93210802_+5944-CCDC18 | 23.23131 | 3.99E-15 | 1.52E-13 | up | chr1 | CCDC18 |
| chr10_101810314_101792839_-17475-MGEA5 | 5.767865 | 0.044813 | 0.207387 | up | chr10 | MGEA5 |
| chr10_110132400_110124017_+8383-ADD3 | 7.537617 | 0.005834 | 0.061225 | up | chr10 | ADD3 |
| chr10_1105267_1072116_+33151-WDR37 | 21.68332 | 2.35E-13 | 3.32E-12 | up | chr10 | WDR37 |
| chr10_110598290_110596398_+1892-SMC3 | 7.09184 | 0.016493 | 0.133382 | up | chr10 | SMC3 |
| chr10_113884380_113876521_+7859-NHLRC2 | 5.968694 | 0.038091 | 0.19255 | up | chr10 | NHLRC2 |
| chr10_124046724_124038515_-8209-CHST15 | 5.930933 | 0.043059 | 0.202782 | up | chr10 | CHST15 |
| chr10_124943307_124942457_+850-AL731577.2 | 5.135559 | 0.027063 | 0.17344 | up | chr10 | AL731577.2 |
| chr10_127000307_126970702_+29605-DOCK1 | 6.062628 | 0.014721 | 0.125754 | up | chr10 | DOCK1 |
| chr10_13136897_13127745_+9152-OPTN | 7.737095 | 0.002048 | 0.023757 | up | chr10 | OPTN |
| chr10_131947791_131934458_+13333-PPP2R2D | 6.157541 | 0.024475 | 0.167803 | up | chr10 | PPP2R2D |
| chr10_15847943_15821656_-26287-MINDY3 | 5.825656 | 0.048982 | 0.214512 | up | chr10 | MINDY3 |
| chr10_16782084_16752539_-29545-RSU1 | 7.712253 | 0.006204 | 0.064501 | up | chr10 | RSU1 |
| chr10_26770345_26759062_-11283-ABI1 | 22.80567 | 1.25E-14 | 2.88E-13 | up | chr10 | ABI1 |
| chr10_28131691_28119651_-12040-MPP7 | 7.096284 | 0.016396 | 0.133382 | up | chr10 | MPP7 |
| chr10_32037607_32034690_-2917-KIF5B | -21.8253 | 1.64E-13 | 2.63E-12 | down | chr10 | KIF5B |
| chr10_32584304_32543300_+41004-CCDC7 | 7.216458 | 0.014687 | 0.125754 | up | chr10 | CCDC7 |
| chr10_50590247_50433476_-156771-SGMS1 | 8.560678 | 0.000809 | 0.009589 | up | chr10 | SGMS1 |
| chr10_5800705_5773842_-26863-GDI2 | 4.871289 | 0.023435 | 0.165811 | up | chr10 | GDI2 |
| chr10_5800705_5785142_-15563-GDI2 | 8.356787 | 0.002122 | 0.024354 | up | chr10 | GDI2 |
| chr10_96952196_96944113_+8083-LCOR | -7.46783 | 0.010657 | 0.097437 | down | chr10 | LCOR |
| chr11_107392895_107390074_-2821-CWF19L2 | 6.032357 | 0.041467 | 0.198682 | up | chr11 | CWF19L2 |
| chr11_108304852_108301648_+3204-ATM | 6.344509 | 0.019971 | 0.154987 | up | chr11 | ATM |
| chr11_108332037_108329021_+3016-ATM | 6.767017 | 0.02215 | 0.161651 | up | chr11 | ATM |
| chr11_118584644_118581246_+3398-ARCN1 | 6.300329 | 0.0332 | 0.184899 | up | chr11 | ARCN1 |
| chr11_128768272_128758115_+10157-FLI1 | -21.5351 | 3.46E-13 | 4.59E-12 | down | chr11 | FLI1 |
| chr11_18292976_18291442_-1534-HPS5 | 5.452772 | 0.027997 | 0.17344 | up | chr11 | HPS5 |
| chr11_32935435_32927157_+8278-QSER1 | 6.68513 | 0.022043 | 0.161651 | up | chr11 | QSER1 |
| chr11_33328633_33286413_+42220-HIPK3 | -6.30411 | 0.033221 | 0.184899 | down | chr11 | HIPK3 |
| chr11_34092056_34090179_+1877-CAPRIN1 | -6.11163 | 0.039018 | 0.192924 | down | chr11 | CAPRIN1 |
| chr11_45979966_45965315_-14651-PHF21A | 6.49353 | 0.028172 | 0.17344 | up | chr11 | PHF21A |
| chr11_47360040_47358844_-1196-SPI1 | 6.271994 | 0.030832 | 0.182669 | up | chr11 | SPI1 |
| chr11_66669291_66668615_-676-RBM4B | -22.3095 | 4.68E-14 | 8.89E-13 | down | chr11 | RBM4B |
| chr11_68564432_68551121_+13311-PPP6R3 | 8.080472 | 0.003318 | 0.036931 | up | chr11 | PPP6R3 |
| chr11_77693611_77683710_-9901-RSF1 | -6.01513 | 0.025045 | 0.170366 | down | chr11 | RSF1 |
| chr12_111418877_111418119_+758-SH2B3 | 23.56649 | 1.60E-15 | 9.82E-14 | up | chr12 | SH2B3 |
| chr12_128815070_128808788_-6282-SLC15A4 | 5.965285 | 0.04379 | 0.204479 | up | chr12 | SLC15A4 |
| chr12_1444750_1371833_+72917-ERC1 | 22.85427 | 1.10E-14 | 2.70E-13 | up | chr12 | ERC1 |
| chr12_28307749_28305649_+2100-CCDC91 | 6.89557 | 0.007503 | 0.074485 | up | chr12 | CCDC91 |
| chr12_28391411_28255581_+135830-CCDC91 | 23.56569 | 1.60E-15 | 9.82E-14 | up | chr12 | CCDC91 |
| chr12_42210680_42210355_-325-YAF2 | -6.30726 | 0.033133 | 0.184899 | down | chr12 | YAF2 |
| chr12_42398994_42374863_+24131-PPHLN1 | 6.598746 | 0.025696 | 0.172664 | up | chr12 | PPHLN1 |
| chr12_66217235_66203711_+13524-IRAK3 | 4.814951 | 0.036869 | 0.192364 | up | chr12 | IRAK3 |
| chr12_69801721_69800209_+1512-RAB3IP | 6.168286 | 0.037078 | 0.192364 | up | chr12 | RAB3IP |
| chr13_100273346_100257595_+15751-PCCA | 23.63083 | 1.34E-15 | 9.82E-14 | up | chr13 | PCCA |
| chr13_30241086_30227412_-13674-KATNAL1 | -6.11114 | 0.039034 | 0.192924 | down | chr13 | KATNAL1 |
| chr13_42970670_42917541_-53129-EPSTI1 | -6.84303 | 0.020757 | 0.157789 | down | chr13 | EPSTI1 |
| chr13_75569507_75560753_+8754-AL137782.1 | 21.67463 | 2.33E-13 | 3.32E-12 | up | chr13 | AL137782.1 |
| chr13_77243951_77205256_-38695-MYCBP2 | -7.57626 | 0.008831 | 0.084837 | down | chr13 | MYCBP2 |
| chr14_102970261_102968253_-2008-CDC42BPB | 6.075159 | 0.04004 | 0.195238 | up | chr14 | CDC42BPB |
| chr14_34802988_34800224_-2764-BAZ1A | 6.258123 | 0.0344 | 0.189543 | up | chr14 | BAZ1A |
| chr14_39327022_39313340_+13682-CTAGE5 | 22.11964 | 7.44E-14 | 1.32E-12 | up | chr14 | CTAGE5 |
| chr14_49831361_49795791_-35570-NEMF | 6.033597 | 0.040744 | 0.196072 | up | chr14 | NEMF |
| chr14_49831361_49825867_-5494-NEMF | 8.026914 | 0.003158 | 0.035881 | up | chr14 | NEMF |
| chr14_57247712_57229734_-17978-EXOC5 | 6.78765 | 0.021353 | 0.161169 | up | chr14 | EXOC5 |
| chr14_69122360_69116366_-5994-DCAF5 | 21.07857 | 1.08E-12 | 1.34E-11 | up | chr14 | DCAF5 |
| chr14_88578140_88574693_+3447-ZC3H14 | -21.5538 | 3.30E-13 | 4.47E-12 | down | chr14 | ZC3H14 |
| chr15_40019200_40009614_+9586-EIF2AK4 | 6.618566 | 0.023733 | 0.166587 | up | chr15 | EIF2AK4 |
| chr15_72046634_72045724_-910-MYO9A | -6.77719 | 0.022019 | 0.161651 | down | chr15 | MYO9A |
| chr15_72208891_72206728_-2163-PKM | -21.5524 | 3.31E-13 | 4.47E-12 | down | chr15 | PKM |
| chr15_82833584_82826410_+7174-WHAMM | 21.75386 | 1.96E-13 | 2.92E-12 | up | chr15 | WHAMM |
| chr15_90492711_90486954_+5757-IQGAP1 | 6.33567 | 0.029351 | 0.178284 | up | chr15 | IQGAP1 |
| chr15_92939718_92924321_+15397-CHD2 | 22.66709 | 1.81E-14 | 3.77E-13 | up | chr15 | CHD2 |
| chr15_94458246_94440176_+18070-MCTP2 | 7.186124 | 0.00893 | 0.084837 | up | chr15 | MCTP2 |
| chr16_24035547_23988508_+47039-PRKCB | 23.418 | 2.40E-15 | 1.12E-13 | up | chr16 | PRKCB |
| chr16_24035547_24032136_+3411-PRKCB | -6.25328 | 0.034675 | 0.189692 | down | chr16 | PRKCB |
| chr16_31723353_31722626_+727-ZNF720 | 22.67418 | 1.78E-14 | 3.77E-13 | up | chr16 | ZNF720 |
| chr16_3770986_3749627_-21359-CREBBP | 6.355662 | 0.031681 | 0.183191 | up | chr16 | CREBBP |
| chr16_53274302_53254438_+19864-CHD9 | 21.71177 | 2.19E-13 | 3.17E-12 | up | chr16 | CHD9 |
| chr16_75414695_75411825_-2870-AC009163.4 | 23.08535 | 5.92E-15 | 1.98E-13 | up | chr16 | AC009163.4 |
| chr16_87984259_87975048_+9211-BANP | -5.89379 | 0.046597 | 0.212191 | down | chr16 | BANP |
| chr17_1100735_1050050_-50685-ABR | -6.44568 | 0.029444 | 0.178284 | down | chr17 | ABR |
| chr17_28172618_28163543_+9075-NLK | 23.69241 | 1.13E-15 | 9.59E-14 | up | chr17 | NLK |
| chr17_50741731_50736960_+4771-LUC7L3 | 6.15008 | 0.037642 | 0.19255 | up | chr17 | LUC7L3 |
| chr17_61985090_61984171_-919-MED13 | 23.13851 | 5.13E-15 | 1.89E-13 | up | chr17 | MED13 |
| chr17_62035608_62029541_-6067-MED13 | 22.71789 | 1.57E-14 | 3.53E-13 | up | chr17 | MED13 |
| chr17_7499491_7499039_+452-POLR2A | -6.1262 | 0.038551 | 0.192924 | down | chr17 | POLR2A |
| chr17_75938232_75937566_-666-FBF1 | 6.340791 | 0.032083 | 0.183191 | up | chr17 | FBF1 |
| chr17_76304941_76287192_-17749-QRICH2 | -7.15888 | 0.01554 | 0.130725 | down | chr17 | QRICH2 |
| chr18_21008194_21006351_-1843-ROCK1 | 21.44669 | 4.29E-13 | 5.63E-12 | up | chr18 | ROCK1 |
| chr18_21044186_21039472_-4714-ROCK1 | 6.252533 | 0.030321 | 0.180613 | up | chr18 | ROCK1 |
| chr18_46946923_46946057_+866-KATNAL2 | 5.3513 | 0.025942 | 0.173261 | up | chr18 | KATNAL2 |
| chr18_50940386_50918110_+22276-ME2 | 23.06328 | 6.29E-15 | 1.98E-13 | up | chr18 | ME2 |
| chr19_11120522_11120092_+430-LDLR | -22.9305 | 9.02E-15 | 2.42E-13 | down | chr19 | LDLR |
| chr19_11514221_11513056_-1165-ECSIT | 24.05541 | 4.12E-16 | 7.45E-14 | up | chr19 | ECSIT |
| chr19_18147624_18147443_+181-MAST3 | -5.81473 | 0.018094 | 0.144489 | down | chr19 | MAST3 |
| chr19_18175697_18175040_+657-IFI30 | 6.057692 | 0.040617 | 0.196072 | up | chr19 | IFI30 |
| chr19_19492712_19492306_+406-GATAD2A | -21.211 | 7.71E-13 | 9.88E-12 | down | chr19 | GATAD2A |
| chr19_40036626_40034950_-1676-na | -6.30726 | 0.033133 | 0.184899 | down | chr19 | na |
| chr19_5082504_5047476_+35028-KDM4B | 22.07782 | 8.40E-14 | 1.47E-12 | up | chr19 | KDM4B |
| chr19_51880877_51877282_-3595-ZNF577 | -6.49919 | 0.028055 | 0.17344 | down | chr19 | ZNF577 |
| chr19_5654456_5653115_+1341-SAFB | 24.14647 | 3.19E-16 | 7.45E-14 | up | chr19 | SAFB |
| chr19_8463686_8455405_+8281-HNRNPM | 6.713897 | 0.013061 | 0.113336 | up | chr19 | HNRNPM |
| chr2_106166083_106122970_-43113-UXS1 | 7.450939 | 0.008034 | 0.078352 | up | chr2 | UXS1 |
| chr2_106166083_106158058_-8025-UXS1 | 6.428504 | 0.029773 | 0.178316 | up | chr2 | UXS1 |
| chr2_135772547_135769769_+2778-UBXN4 | 6.912323 | 0.018858 | 0.149509 | up | chr2 | UBXN4 |
| chr2_1436384_1413264_+23120-TPO | -6.72378 | 0.021643 | 0.161651 | down | chr2 | TPO |
| chr2_189818180_189791790_+26390-PMS1 | 23.79454 | 8.53E-16 | 8.54E-14 | up | chr2 | PMS1 |
| chr2_214792445_214752447_-39998-BARD1 | 23.06314 | 6.29E-15 | 1.98E-13 | up | chr2 | BARD1 |
| chr2_214797117_214767482_-29635-BARD1 | 5.858301 | 0.047724 | 0.213788 | up | chr2 | BARD1 |
| chr2_230361697_230357805_+3892-SP140L | -6.74318 | 0.022697 | 0.163529 | down | chr2 | SP140L |
| chr2_230450255_230440476_+9779-SP100 | 23.30499 | 3.27E-15 | 1.29E-13 | up | chr2 | SP100 |
| chr2_233398449_233388257_+10192-DGKD | -6.16982 | 0.037181 | 0.192364 | down | chr2 | DGKD |
| chr2_238277955_238275743_-2212-PER2 | 6.526773 | 0.027357 | 0.17344 | up | chr2 | PER2 |
| chr2_241434321_241407537_+26784-FARP2 | 21.0516 | 1.16E-12 | 1.42E-11 | up | chr2 | FARP2 |
| chr2_24147086_24135119_+11967-AC008073.3 | 6.555484 | 0.024482 | 0.167803 | up | chr2 | AC008073.3 |
| chr2_29135666_29131258_+4408-CLIP4 | -22.0665 | 8.81E-14 | 1.50E-12 | down | chr2 | CLIP4 |
| chr2_44218537_44209210_+9327-PPM1B | 23.54056 | 1.72E-15 | 9.96E-14 | up | chr2 | PPM1B |
| chr2_45562756_45546732_-16024-SRBD1 | -24.9686 | 3.06E-17 | 3.37E-14 | down | chr2 | SRBD1 |
| chr2_85371562_85368686_+2876-ELMOD3 | 7.70577 | 0.007051 | 0.072126 | up | chr2 | ELMOD3 |
| chr20_35732135_35721740_-10395-RBM39 | 23.01668 | 7.13E-15 | 2.03E-13 | up | chr20 | RBM39 |
| chr20_36839441_36829054_-10387-SOGA1 | -5.89379 | 0.046597 | 0.212191 | down | chr20 | SOGA1 |
| chr20_3912634_3907926_+4708-PANK2 | 22.23691 | 5.51E-14 | 1.01E-12 | up | chr20 | PANK2 |
| chr20_41514952_41512846_-2106-CHD6 | 22.05619 | 8.86E-14 | 1.50E-12 | up | chr20 | CHD6 |
| chr20_47633636_47623911_+9725-NCOA3 | 23.39699 | 2.54E-15 | 1.12E-13 | up | chr20 | NCOA3 |
| chr20_53256571_53253499_+3072-TSHZ2 | -6.15414 | 0.037669 | 0.19255 | down | chr20 | TSHZ2 |
| chr20_62859526_62857395_-2131-TCFL5 | -22.7076 | 1.64E-14 | 3.60E-13 | down | chr20 | TCFL5 |
| chr21_37430400_37420299_+10101-DYRK1A | 23.39376 | 2.57E-15 | 1.12E-13 | up | chr21 | DYRK1A |
| chr21_41257326_41237513_+19813-BACE2 | 23.93625 | 5.75E-16 | 7.45E-14 | up | chr21 | BACE2 |
| chr22_21807846_21805850_-1996-MAPK1 | 22.86914 | 1.06E-14 | 2.65E-13 | up | chr22 | MAPK1 |
| chr22_38248019_38245935_-2084-TMEM184B | 23.38198 | 2.65E-15 | 1.12E-13 | up | chr22 | TMEM184B |
| chr22_38501280_38498085_-3195-DDX17 | 6.920445 | 0.012312 | 0.108289 | up | chr22 | DDX17 |
| chr3_101672213_101671130_-1083-ZBTB11 | 6.738903 | 0.020762 | 0.157789 | up | chr3 | ZBTB11 |
| chr3_109069757_109054728_-15029-MORC1 | 22.32535 | 4.36E-14 | 8.43E-13 | up | chr3 | MORC1 |
| chr3_119504021_119500695_+3326-TIMMDC1 | 23.05293 | 6.47E-15 | 1.98E-13 | up | chr3 | TIMMDC1 |
| chr3_142376594_142347234_-29360-XRN1 | 23.013 | 7.20E-15 | 2.03E-13 | up | chr3 | XRN1 |
| chr3_146124229_146121112_-3117-PLOD2 | -6.30726 | 0.033133 | 0.184899 | down | chr3 | PLOD2 |
| chr3_152447773_152414941_+32832-MBNL1 | 6.168799 | 0.037062 | 0.192364 | up | chr3 | MBNL1 |
| chr3_16303592_16286342_+17250-OXNAD1 | -6.23634 | 0.035172 | 0.189692 | down | chr3 | OXNAD1 |
| chr3_168048365_168024526_-23839-GOLIM4 | 22.80512 | 1.26E-14 | 2.88E-13 | up | chr3 | GOLIM4 |
| chr3_183762279_183736718_+25561-YEATS2 | 6.377045 | 0.031111 | 0.183191 | up | chr3 | YEATS2 |
| chr3_195888606_195878253_-10353-TNK2 | 7.716748 | 0.00907 | 0.085427 | up | chr3 | TNK2 |
| chr3_32454542_32441840_+12702-CMTM7 | -6.02596 | 0.041863 | 0.199711 | down | chr3 | CMTM7 |
| chr3_32545965_32544876_-1089-DYNC1LI1 | -6.65615 | 0.024516 | 0.167803 | down | chr3 | DYNC1LI1 |
| chr3_44840456_44829971_+10485-KIF15 | 6.729047 | 0.022911 | 0.163944 | up | chr3 | KIF15 |
| chr3_47636136_47635190_-946-SMARCC1 | 5.977791 | 0.043298 | 0.203039 | up | chr3 | SMARCC1 |
| chr3_47638780_47622207_-16573-SMARCC1 | 7.098312 | 0.011013 | 0.099477 | up | chr3 | SMARCC1 |
| chr3_47638780_47635190_-3590-SMARCC1 | 7.982378 | 0.004646 | 0.050199 | up | chr3 | SMARCC1 |
| chr3_47678311_47610066_-68245-SMARCC1 | 8.056195 | 0.001661 | 0.019471 | up | chr3 | SMARCC1 |
| chr3_52741499_52737586_-3913-NEK4 | 7.130614 | 0.007935 | 0.078071 | up | chr3 | NEK4 |
| chr3_71053773_71015549_-38224-FOXP1 | -5.58548 | 0.014908 | 0.126376 | down | chr3 | FOXP1 |
| chr4_112585725_112562371_-23354-ZGRF1 | -22.4872 | 2.92E-14 | 5.75E-13 | down | chr4 | ZGRF1 |
| chr4_139168042_139125164_-42878-ELF2 | -6.48022 | 0.028581 | 0.17498 | down | chr4 | ELF2 |
| chr4_147881932_147857553_+24379-ARHGAP10 | 23.05842 | 6.37E-15 | 1.98E-13 | up | chr4 | ARHGAP10 |
| chr4_150817257_150798081_-19176-LRBA | 6.288679 | 0.033527 | 0.185665 | up | chr4 | LRBA |
| chr4_1703700_1694774_-8926-SLBP | 6.354099 | 0.031723 | 0.183191 | up | chr4 | SLBP |
| chr4_3107423_3086939_+20484-HTT | 6.104548 | 0.03904 | 0.192924 | up | chr4 | HTT |
| chr4_53428183_53425872_+2311-AC058822.1 | 20.53082 | 4.02E-12 | 4.82E-11 | up | chr4 | AC058822.1 |
| chr4_7001251_6994184_+7067-TBC1D14 | -21.2056 | 7.96E-13 | 1.01E-11 | down | chr4 | TBC1D14 |
| chr5_132893118_132892164_-954-AFF4 | 23.57106 | 1.58E-15 | 9.82E-14 | up | chr5 | AFF4 |
| chr5_154034967_154029489_-5478-FAM114A2 | 6.503488 | 0.01129 | 0.101154 | up | chr5 | FAM114A2 |
| chr5_157304366_157294783_+9583-CYFIP2 | 6.503435 | 0.027915 | 0.17344 | up | chr5 | CYFIP2 |
| chr5_172057473_172055588_-1885-STK10 | 23.45101 | 2.20E-15 | 1.10E-13 | up | chr5 | STK10 |
| chr5_177050936_177041126_+9810-ZNF346 | 23.47851 | 2.04E-15 | 1.07E-13 | up | chr5 | ZNF346 |
| chr5_177212195_177209636_+2559-NSD1 | -21.7315 | 2.03E-13 | 2.98E-12 | down | chr5 | NSD1 |
| chr5_179720560_179709873_+10687-CANX | 5.878636 | 0.046954 | 0.212936 | up | chr5 | CANX |
| chr5_36986301_36982165_+4136-na | 6.409851 | 0.026957 | 0.17344 | up | chr5 | na |
| chr5_41807438_41794003_-13435-OXCT1 | -22.5966 | 2.20E-14 | 4.40E-13 | down | chr5 | OXCT1 |
| chr5_50411383_50399107_-12276-EMB | 5.821157 | 0.049157 | 0.214512 | up | chr5 | EMB |
| chr5_56111782_56111118_-664-ANKRD55 | -5.82467 | 0.049248 | 0.214512 | down | chr5 | ANKRD55 |
| chr5_71511151_71509465_+1686-BDP1 | -5.93847 | 0.044859 | 0.207387 | down | chr5 | BDP1 |
| chr5_73077493_73074742_+2751-FCHO2 | 6.006801 | 0.027899 | 0.17344 | up | chr5 | FCHO2 |
| chr5_77464809_77449760_-15049-WDR41 | 6.885933 | 0.019908 | 0.154987 | up | chr5 | WDR41 |
| chr5_79623489_79619612_+3877-PAPD4 | 22.88288 | 1.02E-14 | 2.61E-13 | up | chr5 | PAPD4 |
| chr5_81127163_81092801_+34362-RASGRF2 | 7.099323 | 0.01638 | 0.133382 | up | chr5 | RASGRF2 |
| chr5_81127163_81123642_+3521-RASGRF2 | -6.10475 | 0.03924 | 0.193048 | down | chr5 | RASGRF2 |
| chr5_87338091_87331348_+6743-RASA1 | 5.82759 | 0.048907 | 0.214512 | up | chr5 | RASA1 |
| chr6_116660846_116645714_-15132-ZUFSP | 6.608108 | 0.025486 | 0.172307 | up | chr6 | ZUFSP |
| chr6_130184623_130184103_-520-SAMD3 | -5.78921 | 0.048017 | 0.214231 | down | chr6 | SAMD3 |
| chr6_13584225_13579451_+4774-SIRT5 | 7.716978 | 0.00428 | 0.046696 | up | chr6 | SIRT5 |
| chr6_143765798_143765261_+537-PHACTR2 | 6.701698 | 0.023472 | 0.165811 | up | chr6 | PHACTR2 |
| chr6_144803147_144781922_+21225-UTRN | -6.51706 | 0.027685 | 0.17344 | down | chr6 | UTRN |
| chr6_32126252_32120771_-5481-ATF6B | 6.221881 | 0.03546 | 0.189692 | up | chr6 | ATF6B |
| chr6_35642843_35619096_-23747-FKBP5 | 7.41484 | 0.012169 | 0.108151 | up | chr6 | FKBP5 |
| chr6_42606651_42603588_+3063-UBR2 | -6.35787 | 0.031741 | 0.183191 | down | chr6 | UBR2 |
| chr6_72333835_72295937_+37898-RIMS1 | -21.3787 | 5.14E-13 | 6.67E-12 | down | chr6 | RIMS1 |
| chr6_84204096_84185187_-18909-CEP162 | 23.07565 | 6.08E-15 | 1.98E-13 | up | chr6 | CEP162 |
| chr7_100024307_100019231_+5076-ZKSCAN1 | 21.80713 | 1.68E-13 | 2.64E-12 | up | chr7 | ZKSCAN1 |
| chr7_10990847_10982372_+8475-PHF14 | 6.029963 | 0.038318 | 0.192813 | up | chr7 | PHF14 |
| chr7_122133741_122113118_-20623-AASS | -6.74283 | 0.022704 | 0.163529 | down | chr7 | AASS |
| chr7_129018157_129014979_-3178-TNPO3 | 7.548713 | 0.010699 | 0.097437 | up | chr7 | TNPO3 |
| chr7_131399433_131375424_+24009-MKLN1 | 23.34126 | 2.96E-15 | 1.21E-13 | up | chr7 | MKLN1 |
| chr7_131445903_131429033_+16870-MKLN1 | 21.93779 | 1.22E-13 | 1.98E-12 | up | chr7 | MKLN1 |
| chr7_140058034_140054662_-3372-PARP12 | -21.8097 | 1.70E-13 | 2.64E-12 | down | chr7 | PARP12 |
| chr7_152358675_152330601_-28074-KMT2C | -6.2166 | 0.035758 | 0.190365 | down | chr7 | KMT2C |
| chr7_22984045_22976210_-7835-FAM126A | 23.73969 | 9.93E-16 | 9.11E-14 | up | chr7 | FAM126A |
| chr7_24668660_24623666_+44994-MPP6 | 5.846358 | 0.042802 | 0.202782 | up | chr7 | MPP6 |
| chr7_27800090_27785163_+14927-TAX1BP1 | 23.91578 | 6.08E-16 | 7.45E-14 | up | chr7 | TAX1BP1 |
| chr7_40002031_39987599_+14432-CDK13 | -21.1897 | 8.29E-13 | 1.04E-11 | down | chr7 | CDK13 |
| chr7_50327757_50319048_+8709-IKZF1 | -23.9647 | 5.31E-16 | 7.45E-14 | down | chr7 | IKZF1 |
| chr7_70771644_70762870_+8774-AUTS2 | 7.950753 | 0.007177 | 0.072126 | up | chr7 | AUTS2 |
| chr7_92307656_92294889_+12767-ANKIB1 | 6.430034 | 0.027179 | 0.17344 | up | chr7 | ANKIB1 |
| chr8_100288267_100287501_-766-RNF19A | 6.774368 | 0.020388 | 0.157118 | up | chr8 | RNF19A |
| chr8_123337821_123333878_-3943-ATAD2 | 22.94098 | 8.74E-15 | 2.41E-13 | up | chr8 | ATAD2 |
| chr8_123339446_123337625_-1821-ATAD2 | 7.086047 | 0.016582 | 0.133382 | up | chr8 | ATAD2 |
| chr8_140864399_140818277_-46122-PTK2 | -6.93616 | 0.019079 | 0.150179 | down | chr8 | PTK2 |
| chr8_140890769_140864312_-26457-PTK2 | 7.020163 | 0.005333 | 0.056506 | up | chr8 | PTK2 |
| chr8_18011827_18011237_+590-PCM1 | 7.457116 | 0.007122 | 0.072126 | up | chr8 | PCM1 |
| chr8_18872733_18867674_-5059-PSD3 | 21.65356 | 2.54E-13 | 3.54E-12 | up | chr8 | PSD3 |
| chr8_1909470_1876571_+32899-ARHGEF10 | -24.2966 | 2.10E-16 | 7.45E-14 | down | chr8 | ARHGEF10 |
| chr8_47957431_47953620_-3811-PRKDC | 23.02044 | 7.06E-15 | 2.03E-13 | up | chr8 | PRKDC |
| chr8_51861246_51831444_-29802-PCMTD1 | 21.76889 | 1.89E-13 | 2.88E-12 | up | chr8 | PCMTD1 |
| chr8_66593194_66572482_-20712-MYBL1 | 7.115755 | 0.016132 | 0.133382 | up | chr8 | MYBL1 |
| chr8_67116122_67105905_+10217-CSPP1 | 8.158091 | 0.003258 | 0.036637 | up | chr8 | CSPP1 |
| chr8_67137603_67131951_+5652-CSPP1 | 21.94838 | 1.19E-13 | 1.95E-12 | up | chr8 | CSPP1 |
| chr8_68030658_68017846_+12812-PREX2 | 6.142151 | 0.037869 | 0.19255 | up | chr8 | PREX2 |
| chr8_96880005_96834973_+45032-CPQ | -6.07201 | 0.040313 | 0.195703 | down | chr8 | CPQ |
| chr8_99193057_99134632_+58425-VPS13B | 6.234179 | 0.035097 | 0.189692 | up | chr8 | VPS13B |
| chr9_112297916_112250929_-46987-PTBP3 | 21.02233 | 1.25E-12 | 1.51E-11 | up | chr9 | PTBP3 |
| chr9_122859102_122855184_-3918-RC3H2 | 5.933149 | 0.044978 | 0.207387 | up | chr9 | RC3H2 |
| chr9_33989126_33953285_-35841-UBAP2 | 6.505483 | 0.027865 | 0.17344 | up | chr9 | UBAP2 |
| chr9_37206494_37126312_+80182-ZCCHC7 | 23.47788 | 2.04E-15 | 1.07E-13 | up | chr9 | ZCCHC7 |
| chr9_77227485_77214329_+13156-VPS13A | 6.306876 | 0.012381 | 0.108289 | up | chr9 | VPS13A |
| chr9_83686155_83679775_-6380-UBQLN1 | 6.134868 | 0.038076 | 0.19255 | up | chr9 | UBQLN1 |
| chr9_85712566_85669485_-43081-AGTPBP1 | 6.087323 | 0.036351 | 0.192364 | up | chr9 | AGTPBP1 |
| chr9_86040868_86033282_-7586-GOLM1 | 6.573629 | 0.026265 | 0.17344 | up | chr9 | GOLM1 |
| chr9_96471094_96458379_+12715-HABP4 | 5.402165 | 0.048804 | 0.214512 | up | chr9 | HABP4 |
| chr9_97350555_97347314_+3241-CCDC180 | 24.31589 | 1.98E-16 | 7.45E-14 | up | chr9 | CCDC180 |
| chrX_135556300_135545423_+10877-INTS6L | 21.54699 | 3.33E-13 | 4.47E-12 | up | chrX | INTS6L |
| chrX_17138961_17103718_+35243-REPS2 | -6.23287 | 0.035274 | 0.189692 | down | chrX | REPS2 |
| chrX_19695741_19683823_-11918-SH3KBP1 | -24.1046 | 3.60E-16 | 7.45E-14 | down | chrX | SH3KBP1 |
| chrX_53615835_53614534_-1301-HUWE1 | 22.29718 | 4.80E-14 | 8.97E-13 | up | chrX | HUWE1 |
| chrY_2961646_2953909_+7737-ZFY | 4.651257 | 0.042894 | 0.202782 | up | chrY | ZFY |
