# Supplementary material for: Expression Profiles and Functional Analysis of Plasma Exosomal Circular RNAs in Acute Myocardial Infarction
Source: Biomed Res Int. 2022 Oct 1;2022:3458227. doi: 10.1155/2022/3458227 (PMC9547997; doi:10.1155/2022/3458227)
Supplement: Supplementary 3 — Supplementary Table S3: Differentially expressed exosomal circRNAs based on the screening criteria of fold change ≥1 and P < 0.05 in comparison of CAD and control. [file 3458227.f3.docx]

Supplementary Table S3 Differentially expressed exosomal circRNAs based on the screening criteria of fold change ≥ 1 and P < 0.05 in comparison of CAD and control.

| circRNA ID | log2FC | Pvalue | FDR | Style | CHROM | GeneName |
| --- | --- | --- | --- | --- | --- | --- |
| chr1_10105744_10095461_+10283-UBE4B | -6.0285 | 0.044199 | 0.204661 | down | chr1 | UBE4B |
| chr1_108185954_108154983_-30971-SLC25A24 | -22.2792 | 9.80E-14 | 2.30E-12 | down | chr1 | SLC25A24 |
| chr1_117466427_117402186_+64241-MAN1A2 | 6.074791 | 0.035732 | 0.195526 | up | chr1 | MAN1A2 |
| chr1_151639119_151638888_+231-SNX27 | -20.8607 | 3.30E-12 | 4.83E-11 | down | chr1 | SNX27 |
| chr1_15536814_15534237_+2577-DNAJC16 | -6.39042 | 0.032868 | 0.187298 | down | chr1 | DNAJC16 |
| chr1_180993425_180984677_-8748-STX6 | -6.00171 | 0.045155 | 0.20626 | down | chr1 | STX6 |
| chr1_205620838_205618957_-1881-ELK4 | -6.1981 | 0.038532 | 0.196951 | down | chr1 | ELK4 |
| chr1_20773610_20749723_-23887-HP1BP3 | -6.39079 | 0.032858 | 0.187298 | down | chr1 | HP1BP3 |
| chr1_20942330_20904883_-37447-EIF4G3 | -6.70234 | 0.025199 | 0.164113 | down | chr1 | EIF4G3 |
| chr1_20981227_20969474_-11753-EIF4G3 | 7.664636 | 0.009987 | 0.094068 | up | chr1 | EIF4G3 |
| chr1_21002808_20969474_-33334-EIF4G3 | -6.12598 | 0.040862 | 0.199168 | down | chr1 | EIF4G3 |
| chr1_243843282_243572926_-270356-AKT3 | 6.035992 | 0.038525 | 0.196951 | up | chr1 | AKT3 |
| chr1_246591941_246591512_+429-CNST | -7.55218 | 0.01163 | 0.102424 | down | chr1 | CNST |
| chr1_31007099_30992390_-14709-PUM1 | -23.1455 | 1.04E-14 | 5.52E-13 | down | chr1 | PUM1 |
| chr1_33295305_33294937_+368-ZNF362 | 6.39956 | 0.027751 | 0.165712 | up | chr1 | ZNF362 |
| chr1_36173478_36170971_+2507-MAP7D1 | 24.4185 | 2.23E-16 | 6.48E-14 | up | chr1 | MAP7D1 |
| chr1_51408332_51402435_-5897-EPS15 | 7.122544 | 0.016577 | 0.131092 | up | chr1 | EPS15 |
| chr1_52509712_52493611_-16101-ZCCHC11 | -22.5447 | 4.96E-14 | 1.48E-12 | down | chr1 | ZCCHC11 |
| chr1_70315566_70292388_-23178-ANKRD13C | -5.64929 | 0.037773 | 0.196951 | down | chr1 | ANKRD13C |
| chr1_93877862_93876268_-1594-DNTTIP2 | -20.7326 | 4.52E-12 | 6.15E-11 | down | chr1 | DNTTIP2 |
| chr10_101813606_101792839_-20767-MGEA5 | 5.82223 | 0.04371 | 0.204067 | up | chr10 | MGEA5 |
| chr10_110985765_110964125_+21640-SHOC2 | 7.784493 | 0.007461 | 0.078489 | up | chr10 | SHOC2 |
| chr10_12120267_12081472_+38795-DHTKD1 | 23.98071 | 7.56E-16 | 1.17E-13 | up | chr10 | DHTKD1 |
| chr10_124943307_124940374_+2933-ZRANB1 | 6.430308 | 0.030559 | 0.179417 | up | chr10 | ZRANB1 |
| chr10_12666810_12649654_+17156-CAMK1D | 6.688578 | 0.024609 | 0.163009 | up | chr10 | CAMK1D |
| chr10_30029866_30026103_-3763-JCAD | 9.35401 | 0.000442 | 0.005273 | up | chr10 | JCAD |
| chr10_32037607_32034690_-2917-KIF5B | -21.0174 | 2.27E-12 | 3.38E-11 | down | chr10 | KIF5B |
| chr10_32481782_32451592_+30190-CCDC7 | -6.05142 | 0.043394 | 0.203821 | down | chr10 | CCDC7 |
| chr10_4908420_4830675_+77745-AKR1E2 | 6.122753 | 0.039711 | 0.197336 | up | chr10 | AKR1E2 |
| chr10_4909606_4830675_+78931-AKR1E2 | -21.9723 | 2.13E-13 | 4.14E-12 | down | chr10 | AKR1E2 |
| chr10_5800705_5773842_-26863-GDI2 | 4.806849 | 0.049243 | 0.207713 | up | chr10 | GDI2 |
| chr10_71732375_71730469_+1906-CDH23 | 22.03231 | 1.36E-13 | 2.92E-12 | up | chr10 | CDH23 |
| chr10_7381949_7367649_-14300-SFMBT2 | 7.016346 | 0.018378 | 0.140135 | up | chr10 | SFMBT2 |
| chr11_108332037_108329021_+3016-ATM | 7.325426 | 0.004907 | 0.055112 | up | chr11 | ATM |
| chr11_118584644_118581246_+3398-ARCN1 | 7.051884 | 0.017793 | 0.137897 | up | chr11 | ARCN1 |
| chr11_118584644_118583809_+835-ARCN1 | -6.67368 | 0.025833 | 0.165625 | down | chr11 | ARCN1 |
| chr11_121060085_121045674_+14411-TBCEL | -8.54314 | 0.000145 | 0.001755 | down | chr11 | TBCEL |
| chr11_128490576_128480191_-10385-ETS1 | -6.99728 | 0.019427 | 0.146176 | down | chr11 | ETS1 |
| chr11_128768272_128758115_+10157-FLI1 | -20.3023 | 1.27E-11 | 1.66E-10 | down | chr11 | FLI1 |
| chr11_32935435_32927157_+8278-QSER1 | 24.14731 | 4.77E-16 | 9.24E-14 | up | chr11 | QSER1 |
| chr11_77693611_77683710_-9901-RSF1 | -22.8895 | 2.03E-14 | 8.01E-13 | down | chr11 | RSF1 |
| chr11_96093517_96091892_-1625-MAML2 | 23.62604 | 2.00E-15 | 1.94E-13 | up | chr11 | MAML2 |
| chr12_111683307_111679151_-4156-BRAP | 7.572053 | 0.008404 | 0.082444 | up | chr12 | BRAP |
| chr12_1190052_1115866_+74186-ERC1 | 6.014663 | 0.043354 | 0.203821 | up | chr12 | ERC1 |
| chr12_12244655_12244262_-393-LRP6 | 6.124841 | 0.039644 | 0.197336 | up | chr12 | LRP6 |
| chr12_26632059_26621123_-10936-ITPR2 | 21.23321 | 9.91E-13 | 1.63E-11 | up | chr12 | ITPR2 |
| chr12_42210680_42210355_-325-YAF2 | -6.12598 | 0.040862 | 0.199168 | down | chr12 | YAF2 |
| chr12_45964188_45945249_-18939-SCAF11 | -5.96772 | 0.046394 | 0.20626 | down | chr12 | SCAF11 |
| chr12_79822190_79793863_-28327-PPP1R12A | 6.133629 | 0.027773 | 0.165712 | up | chr12 | PPP1R12A |
| chr12_897681_894562_+3119-WNK1 | 9.267864 | 0.000489 | 0.005743 | up | chr12 | WNK1 |
| chr13_28220378_28174272_+46106-PAN3 | 6.22901 | 0.036388 | 0.196392 | up | chr13 | PAN3 |
| chr13_37051583_37040405_-11178-SUPT20H | 8.185534 | 0.004725 | 0.053854 | up | chr13 | SUPT20H |
| chr13_42970670_42917541_-53129-EPSTI1 | -6.84544 | 0.022235 | 0.155474 | down | chr13 | EPSTI1 |
| chr13_52402681_52397232_-5449-THSD1 | 6.892682 | 0.020544 | 0.148801 | up | chr13 | THSD1 |
| chr14_102040673_102040236_+437-DYNC1H1 | 7.624108 | 0.007642 | 0.078489 | up | chr14 | DYNC1H1 |
| chr14_24211829_24210348_-1481-AL136295.1 | 7.012389 | 0.018444 | 0.140135 | up | chr14 | AL136295.1 |
| chr14_32094386_32090502_+3884-ARHGAP5 | -22.0569 | 1.72E-13 | 3.43E-12 | down | chr14 | ARHGAP5 |
| chr14_49831361_49825867_-5494-NEMF | 6.327423 | 0.033524 | 0.187333 | up | chr14 | NEMF |
| chr14_52544371_52511240_-33131-TXNDC16 | 21.52745 | 4.89E-13 | 8.82E-12 | up | chr14 | TXNDC16 |
| chr14_61457679_61443111_+14568-PRKCH | 22.52679 | 3.77E-14 | 1.33E-12 | up | chr14 | PRKCH |
| chr14_73297285_73282359_-14926-NUMB | -6.66553 | 0.026015 | 0.165625 | down | chr14 | NUMB |
| chr15_59031702_59030804_+898-RNF111 | -5.93199 | 0.047727 | 0.207713 | down | chr15 | RNF111 |
| chr15_77471814_77467095_+4719-HMG20A | 6.742156 | 0.02348 | 0.158451 | up | chr15 | HMG20A |
| chr15_78471924_78470532_+1392-IREB2 | -5.9671 | 0.046417 | 0.20626 | down | chr15 | IREB2 |
| chr15_90443478_90439332_+4146-IQGAP1 | -7.13739 | 0.017117 | 0.133994 | down | chr15 | IQGAP1 |
| chr15_90492711_90486954_+5757-IQGAP1 | 22.81696 | 1.76E-14 | 7.96E-13 | up | chr15 | IQGAP1 |
| chr15_92956649_92937518_+19131-CHD2 | 5.925322 | 0.046575 | 0.20626 | up | chr15 | CHD2 |
| chr15_92967513_92945821_+21692-CHD2 | -23.2059 | 8.78E-15 | 5.52E-13 | down | chr15 | CHD2 |
| chr15_94402019_94356137_+45882-MCTP2 | 22.45906 | 4.50E-14 | 1.39E-12 | up | chr15 | MCTP2 |
| chr16_17259498_17258988_-510-XYLT1 | 22.46213 | 4.46E-14 | 1.39E-12 | up | chr16 | XYLT1 |
| chr16_24035547_24032136_+3411-PRKCB | -6.37028 | 0.033426 | 0.187333 | down | chr16 | PRKCB |
| chr16_31723353_31722626_+727-ZNF720 | 23.43616 | 3.35E-15 | 2.60E-13 | up | chr16 | ZNF720 |
| chr16_3745354_3736036_-9318-CREBBP | 6.427455 | 0.030813 | 0.179552 | up | chr16 | CREBBP |
| chr16_53274302_53255600_+18702-CHD9 | -6.35929 | 0.033599 | 0.187333 | down | chr16 | CHD9 |
| chr16_67612121_67610824_+1297-CTCF | 22.25972 | 7.55E-14 | 1.84E-12 | up | chr16 | CTCF |
| chr16_81908591_81895807_+12784-PLCG2 | 7.355413 | 0.013431 | 0.113143 | up | chr16 | PLCG2 |
| chr16_88611536_88598178_+13358-ZC3H18 | 22.06673 | 1.24E-13 | 2.75E-12 | up | chr16 | ZC3H18 |
| chr17_1100735_1050050_-50685-ABR | -6.26445 | 0.036491 | 0.196392 | down | chr17 | ABR |
| chr17_29703062_29684563_-18499-SSH2 | -6.1807 | 0.039084 | 0.197336 | down | chr17 | SSH2 |
| chr17_39715939_39709813_+6126-ERBB2 | 7.518696 | 0.011205 | 0.100974 | up | chr17 | ERBB2 |
| chr17_41983636_41981855_-1781-DNAJC7 | 5.865713 | 0.048834 | 0.207713 | up | chr17 | DNAJC7 |
| chr17_56862251_56848696_+13555-DGKE | 22.75485 | 2.07E-14 | 8.01E-13 | up | chr17 | DGKE |
| chr17_76304941_76287192_-17749-QRICH2 | -7.3875 | 0.013587 | 0.113224 | down | chr17 | QRICH2 |
| chr17_78087093_78079395_+7698-TNRC6C | -22.5099 | 5.43E-14 | 1.50E-12 | down | chr17 | TNRC6C |
| chr17_78093763_78079395_+14368-TNRC6C | 6.484206 | 0.027363 | 0.165712 | up | chr17 | TNRC6C |
| chr17_80925480_80922724_+2756-RPTOR | -6.61444 | 0.027185 | 0.165712 | down | chr17 | RPTOR |
| chr18_13682105_13681605_-500-FAM210A | -21.2354 | 1.33E-12 | 2.11E-11 | down | chr18 | FAM210A |
| chr18_2892486_2884964_+7522-EMILIN2 | -5.9671 | 0.046417 | 0.20626 | down | chr18 | EMILIN2 |
| chr18_2892486_2890561_+1925-EMILIN2 | 7.790975 | 0.008764 | 0.084904 | up | chr18 | EMILIN2 |
| chr18_54287411_54277703_+9708-POLI | 6.745483 | 0.023411 | 0.158451 | up | chr18 | POLI |
| chr19_10177367_10163326_-14041-DNMT1 | -23.6939 | 2.39E-15 | 2.05E-13 | down | chr19 | DNMT1 |
| chr19_19492712_19492306_+406-GATAD2A | -21.2668 | 1.23E-12 | 1.99E-11 | down | chr19 | GATAD2A |
| chr19_40036626_40034950_-1676-na | -6.12598 | 0.040862 | 0.199168 | down | chr19 | na |
| chr19_48913564_48913011_+553-NUCB1 | 8.105273 | 0.005236 | 0.057975 | up | chr19 | NUCB1 |
| chr19_5082504_5047476_+35028-KDM4B | 6.053577 | 0.042011 | 0.203037 | up | chr19 | KDM4B |
| chr19_51880877_51877282_-3595-ZNF577 | -6.89589 | 0.021266 | 0.1512 | down | chr19 | ZNF577 |
| chr19_58263676_58260851_+2825-na | -21.4004 | 8.88E-13 | 1.50E-11 | down | chr19 | na |
| chr19_8548317_8548036_-281-MYO1F | 6.759672 | 0.02312 | 0.158451 | up | chr19 | MYO1F |
| chr2_112311937_112299849_+12088-ZC3H6 | -20.2364 | 1.48E-11 | 1.91E-10 | down | chr2 | ZC3H6 |
| chr2_135639122_135631718_+7404-R3HDM1 | 22.79097 | 1.85E-14 | 7.96E-13 | up | chr2 | R3HDM1 |
| chr2_1436384_1413264_+23120-TPO | -23.829 | 1.67E-15 | 1.84E-13 | down | chr2 | TPO |
| chr2_1456282_1413264_+43018-TPO | 6.921047 | 0.01472 | 0.121361 | up | chr2 | TPO |
| chr2_168129758_168063500_-66258-STK39 | -6.07902 | 0.042441 | 0.203037 | down | chr2 | STK39 |
| chr2_201133134_201129729_+3405-CFLAR | 21.94259 | 1.71E-13 | 3.43E-12 | up | chr2 | CFLAR |
| chr2_201149835_201129729_+20106-CFLAR | 7.738156 | 0.009315 | 0.089126 | up | chr2 | CFLAR |
| chr2_214781509_214752447_-29062-BARD1 | -19.4123 | 9.23E-11 | 1.14E-09 | down | chr2 | BARD1 |
| chr2_230361697_230357805_+3892-SP140L | -6.97139 | 0.019883 | 0.146756 | down | chr2 | SP140L |
| chr2_230450255_230440476_+9779-SP100 | 21.47798 | 5.58E-13 | 9.61E-12 | up | chr2 | SP100 |
| chr2_233398449_233388257_+10192-DGKD | -6.28676 | 0.035825 | 0.195526 | down | chr2 | DGKD |
| chr2_29135666_29131258_+4408-CLIP4 | -21.8795 | 2.70E-13 | 5.10E-12 | down | chr2 | CLIP4 |
| chr2_40430301_40428473_-1828-SLC8A1 | -21.7482 | 3.66E-13 | 6.75E-12 | down | chr2 | SLC8A1 |
| chr2_45562756_45546732_-16024-SRBD1 | -24.5477 | 2.31E-16 | 6.48E-14 | down | chr2 | SRBD1 |
| chr2_61526521_61522611_-3910-XPO1 | 5.865542 | 0.048841 | 0.207713 | up | chr2 | XPO1 |
| chr2_71427414_71426460_+954-ZNF638 | 6.802181 | 0.022268 | 0.155474 | up | chr2 | ZNF638 |
| chr20_35659014_35653528_-5486-AL109827.1 | 7.032007 | 0.013328 | 0.113143 | up | chr20 | AL109827.1 |
| chr20_35732135_35716740_-15395-RBM39 | 24.37741 | 2.51E-16 | 6.48E-14 | up | chr20 | RBM39 |
| chr20_37068186_37065424_-2762-RBL1 | -7.62502 | 0.010847 | 0.098903 | down | chr20 | RBL1 |
| chr20_38066256_38057532_+8724-RPRD1B | -23.1611 | 9.94E-15 | 5.52E-13 | down | chr20 | RPRD1B |
| chr20_53256571_53253499_+3072-TSHZ2 | -5.9728 | 0.046207 | 0.20626 | down | chr20 | TSHZ2 |
| chr20_58673711_58667490_+6221-STX16 | 23.25411 | 5.48E-15 | 3.86E-13 | up | chr20 | STX16 |
| chr20_62859526_62857395_-2131-TCFL5 | -22.5211 | 5.27E-14 | 1.50E-12 | down | chr20 | TCFL5 |
| chr21_39212707_39206108_-6599-BRWD1 | -23.1096 | 1.14E-14 | 5.52E-13 | down | chr21 | BRWD1 |
| chr22_21807846_21799012_-8834-MAPK1 | -5.97614 | 0.046085 | 0.20626 | down | chr22 | MAPK1 |
| chr22_38501280_38499400_-1880-DDX17 | 21.47798 | 5.58E-13 | 9.61E-12 | up | chr22 | DDX17 |
| chr3_125331238_125277726_-53512-ZNF148 | -20.6948 | 4.68E-12 | 6.25E-11 | down | chr3 | ZNF148 |
| chr3_170178938_170136419_-42519-PHC3 | 6.03472 | 0.038628 | 0.196951 | up | chr3 | PHC3 |
| chr3_195888606_195878253_-10353-TNK2 | 22.26392 | 7.47E-14 | 1.84E-12 | up | chr3 | TNK2 |
| chr3_32454542_32441840_+12702-CMTM7 | -6.20215 | 0.038405 | 0.196951 | down | chr3 | CMTM7 |
| chr3_32545965_32544876_-1089-DYNC1LI1 | -7.17945 | 0.016472 | 0.131092 | down | chr3 | DYNC1LI1 |
| chr3_47098081_47067070_-31011-SETD2 | 7.603354 | 0.007798 | 0.078489 | up | chr3 | SETD2 |
| chr3_47678311_47610066_-68245-SMARCC1 | 6.612828 | 0.026286 | 0.165625 | up | chr3 | SMARCC1 |
| chr3_65442849_65429520_-13329-MAGI1 | -20.8509 | 3.40E-12 | 4.88E-11 | down | chr3 | MAGI1 |
| chr4_10103986_10097711_-6275-WDR1 | 8.255014 | 0.00552 | 0.060249 | up | chr4 | WDR1 |
| chr4_127939997_127921524_-18473-MFSD8 | 7.929474 | 0.007689 | 0.078489 | up | chr4 | MFSD8 |
| chr4_128948570_128936655_-11915-SCLT1 | 7.690651 | 0.007725 | 0.078489 | up | chr4 | SCLT1 |
| chr4_139168042_139125164_-42878-ELF2 | -6.23391 | 0.037419 | 0.196951 | down | chr4 | ELF2 |
| chr4_177353728_177353308_+420-NEIL3 | 6.901417 | 0.020384 | 0.148801 | up | chr4 | NEIL3 |
| chr4_7001251_6994184_+7067-TBC1D14 | -20.818 | 3.68E-12 | 5.19E-11 | down | chr4 | TBC1D14 |
| chr4_76144473_76134175_-10298-NUP54 | -6.9726 | 0.019841 | 0.146756 | down | chr4 | NUP54 |
| chr4_87115299_87114367_+932-AFF1 | 6.856134 | 0.021226 | 0.1512 | up | chr4 | AFF1 |
| chr4_94586444_94573351_+13093-PDLIM5 | -6.16082 | 0.039722 | 0.197336 | down | chr4 | PDLIM5 |
| chr5_41807438_41794003_-13435-OXCT1 | -5.753 | 0.046983 | 0.206884 | down | chr5 | OXCT1 |
| chr5_43161931_43161249_+682-ZNF131 | -5.8985 | 0.049006 | 0.207713 | down | chr5 | ZNF131 |
| chr5_69311204_69294793_-16411-CCDC125 | 6.258985 | 0.024065 | 0.160782 | up | chr5 | CCDC125 |
| chr5_71504751_71495250_+9501-BDP1 | 7.648486 | 0.010146 | 0.094068 | up | chr5 | BDP1 |
| chr5_77048272_77046347_+1925-AGGF1 | -6.61567 | 0.027156 | 0.165712 | down | chr5 | AGGF1 |
| chr5_81127163_81092801_+34362-RASGRF2 | 5.952606 | 0.027551 | 0.165712 | up | chr5 | RASGRF2 |
| chr5_81127163_81123642_+3521-RASGRF2 | -5.88865 | 0.049388 | 0.207713 | down | chr5 | RASGRF2 |
| chr6_130184623_130154825_-29798-SAMD3 | -7.56213 | 0.008016 | 0.079645 | down | chr6 | SAMD3 |
| chr6_136698682_136694140_-4542-MAP3K5 | 6.740584 | 0.023512 | 0.158451 | up | chr6 | MAP3K5 |
| chr6_144516948_144513909_+3039-UTRN | 6.175251 | 0.038038 | 0.196951 | up | chr6 | UTRN |
| chr6_145894977_145864388_-30589-SHPRH | -8.01884 | 0.007379 | 0.078489 | down | chr6 | SHPRH |
| chr6_167040339_167022409_+17930-FGFR1OP | 22.11144 | 1.11E-13 | 2.53E-12 | up | chr6 | FGFR1OP |
| chr6_32126252_32120771_-5481-ATF6B | 22.37522 | 5.60E-14 | 1.50E-12 | up | chr6 | ATF6B |
| chr6_42606651_42603588_+3063-UBR2 | -6.07819 | 0.042405 | 0.203037 | down | chr6 | UBR2 |
| chr6_72333835_72295937_+37898-RIMS1 | -21.1896 | 1.49E-12 | 2.31E-11 | down | chr6 | RIMS1 |
| chr6_75647801_75621532_+26269-SENP6 | -23.1149 | 1.12E-14 | 5.52E-13 | down | chr6 | SENP6 |
| chr7_105091314_105090009_+1305-KMT2E | 7.224319 | 0.015183 | 0.123864 | up | chr7 | KMT2E |
| chr7_122133741_122113118_-20623-AASS | -6.56169 | 0.02844 | 0.168254 | down | chr7 | AASS |
| chr7_140058034_140054662_-3372-PARP12 | -21.1604 | 1.60E-12 | 2.43E-11 | down | chr7 | PARP12 |
| chr7_152315338_152309966_-5372-KMT2C | 7.365409 | 0.013305 | 0.113143 | up | chr7 | KMT2C |
| chr7_155685052_155665175_+19877-RBM33 | 6.254866 | 0.03126 | 0.180794 | up | chr7 | RBM33 |
| chr7_18594029_18585281_+8748-HDAC9 | 22.4985 | 4.05E-14 | 1.37E-12 | up | chr7 | HDAC9 |
| chr7_27649633_27629371_-20262-HIBADH | -7.36923 | 0.010196 | 0.094068 | down | chr7 | HIBADH |
| chr7_40002031_39987599_+14432-CDK13 | -20.7991 | 3.85E-12 | 5.33E-11 | down | chr7 | CDK13 |
| chr7_66286709_66240325_+46384-TPST1 | 23.78815 | 1.29E-15 | 1.66E-13 | up | chr7 | TPST1 |
| chr8_123337821_123333878_-3943-ATAD2 | 8.654596 | 0.002902 | 0.03357 | up | chr8 | ATAD2 |
| chr8_37877551_37862737_-14814-RAB11FIP1 | 22.2575 | 7.59E-14 | 1.84E-12 | up | chr8 | RAB11FIP1 |
| chr8_51861246_51831444_-29802-PCMTD1 | 6.2008 | 0.037246 | 0.196951 | up | chr8 | PCMTD1 |
| chr8_55969793_55941855_+27938-LYN | 6.653624 | 0.025142 | 0.164113 | up | chr8 | LYN |
| chr8_61653660_61618978_-34682-ASPH | 7.423069 | 0.012599 | 0.109709 | up | chr8 | ASPH |
| chr8_96880005_96834973_+45032-CPQ | -6.58847 | 0.027797 | 0.165712 | down | chr8 | CPQ |
| chr9_107311737_107300141_+11596-RAD23B | -7.21431 | 0.015954 | 0.128794 | down | chr9 | RAD23B |
| chr9_137744090_137728349_+15741-EHMT1 | -19.7873 | 3.93E-11 | 4.92E-10 | down | chr9 | EHMT1 |
| chr9_2123937_2096657_+27280-SMARCA2 | -20.2022 | 1.61E-11 | 2.04E-10 | down | chr9 | SMARCA2 |
| chr9_83678599_83677727_-872-UBQLN1 | 6.029472 | 0.042839 | 0.203682 | up | chr9 | UBQLN1 |
| chrX_110109146_110020352_+88794-TMEM164 | 22.61206 | 3.01E-14 | 1.11E-12 | up | chrX | TMEM164 |
| chrX_118590302_118584735_+5567-DOCK11 | 21.96609 | 1.61E-13 | 3.37E-12 | up | chrX | DOCK11 |
| chrX_119653040_119640692_-12348-SEPT6 | -7.56563 | 0.011482 | 0.102281 | down | chrX | Sep6 |
| chrX_17138961_17103718_+35243-REPS2 | -6.34985 | 0.034 | 0.188212 | down | chrX | REPS2 |
| chrX_19695741_19683823_-11918-SH3KBP1 | -5.89157 | 0.044365 | 0.204661 | down | chrX | SH3KBP1 |
| chrY_2961646_2953909_+7737-ZFY | 6.277634 | 0.026253 | 0.165625 | up | chrY | ZFY |
