# Supplementary material for: Expression Profiles and Functional Analysis of Plasma Exosomal Circular RNAs in Acute Myocardial Infarction
Source: Biomed Res Int. 2022 Oct 1;2022:3458227. doi: 10.1155/2022/3458227 (PMC9547997; doi:10.1155/2022/3458227)
Supplement: Supplementary 4 — Supplementary Table S4: Differentially expressed exosomal circRNAs based on the screening criteria of fold change ≥1 and P < 0.05 in comparison of AMI and CAD. [file 3458227.f4.docx]

Supplementary Table S4 Differentially expressed exosomal circRNAs based on the screening criteria of fold change ≥ 1 and P < 0.05 in comparison of AMI and CAD.

| circRNA ID | log2FC | Pvalue | FDR | Style | CHROM | GeneName |
| --- | --- | --- | --- | --- | --- | --- |
| chr1_13782831_13778418_+4413-PRDM2 | 23.64302 | 2.77E-15 | 1.99E-13 | up | chr1 | PRDM2 |
| chr1_145794196_145771711_-22485-RNF115 | -6.12338 | 0.039721 | 0.21325 | down | chr1 | RNF115 |
| chr1_155459898_155415744_-44154-ASH1L | 20.88017 | 3.04E-12 | 4.47E-11 | up | chr1 | ASH1L |
| chr1_155521618_155521100_-518-ASH1L | 7.170289 | 0.016519 | 0.138965 | up | chr1 | ASH1L |
| chr1_15643650_15626669_+16981-DDI2 | 6.21753 | 0.037879 | 0.209122 | up | chr1 | DDI2 |
| chr1_184711877_184706643_-5234-EDEM3 | 22.50849 | 5.52E-14 | 1.33E-12 | up | chr1 | EDEM3 |
| chr1_224974153_224952670_+21483-DNAH14 | 7.652189 | 0.002106 | 0.0255 | up | chr1 | DNAH14 |
| chr1_230961303_230954333_-6970-TTC13 | 5.872743 | 0.032068 | 0.196358 | up | chr1 | TTC13 |
| chr1_235214026_235194013_-20013-ARID4B | 23.62741 | 2.89E-15 | 1.99E-13 | up | chr1 | ARID4B |
| chr1_245929937_245915530_-14407-SMYD3 | 23.61272 | 3.00E-15 | 1.99E-13 | up | chr1 | SMYD3 |
| chr1_28697084_28683583_+13501-GMEB1 | 6.570078 | 0.028208 | 0.18918 | up | chr1 | GMEB1 |
| chr1_31007102_30992390_-14712-PUM1 | 5.764156 | 0.048516 | 0.226783 | up | chr1 | PUM1 |
| chr1_33280457_33280124_+333-ZNF362 | 22.95823 | 1.70E-14 | 5.01E-13 | up | chr1 | ZNF362 |
| chr1_35389082_35381259_+7823-ZMYM4 | -6.07838 | 0.041206 | 0.216162 | down | chr1 | ZMYM4 |
| chr1_36173478_36170971_+2507-MAP7D1 | -23.5649 | 2.35E-15 | 1.99E-13 | down | chr1 | MAP7D1 |
| chr1_51408332_51403419_-4913-EPS15 | 6.015545 | 0.0446 | 0.220617 | up | chr1 | EPS15 |
| chr1_52509712_52493611_-16101-ZCCHC11 | 6.383427 | 0.033023 | 0.196358 | up | chr1 | ZCCHC11 |
| chr1_77584655_77576068_-8587-ZZZ3 | 5.944569 | 0.04719 | 0.226343 | up | chr1 | ZZZ3 |
| chr1_97549744_97515726_-34018-DPYD | 8.163943 | 0.006368 | 0.065533 | up | chr1 | DPYD |
| chr10_101813606_101792839_-20767-MGEA5 | -6.07269 | 0.041397 | 0.216162 | down | chr10 | MGEA5 |
| chr10_104018908_104008177_+10731-SLK | 6.953248 | 0.02019 | 0.157362 | up | chr10 | SLK |
| chr10_110130486_110124017_+6469-ADD3 | 8.056243 | 0.003335 | 0.039282 | up | chr10 | ADD3 |
| chr10_110132400_110124017_+8383-ADD3 | 23.99162 | 1.66E-17 | 3.52E-15 | up | chr10 | ADD3 |
| chr10_1105267_1072116_+33151-WDR37 | 22.24082 | 1.10E-13 | 2.47E-12 | up | chr10 | WDR37 |
| chr10_113884380_113876521_+7859-NHLRC2 | 6.518604 | 0.024987 | 0.176582 | up | chr10 | NHLRC2 |
| chr10_12666810_12649654_+17156-CAMK1D | -6.68531 | 0.024694 | 0.176582 | down | chr10 | CAMK1D |
| chr10_13136897_13127745_+9152-OPTN | 8.44834 | 0.000969 | 0.012522 | up | chr10 | OPTN |
| chr10_131947791_131934458_+13333-PPP2R2D | 6.932624 | 0.020564 | 0.157957 | up | chr10 | PPP2R2D |
| chr10_15847943_15833630_-14313-MINDY3 | -5.98984 | 0.014079 | 0.123337 | down | chr10 | MINDY3 |
| chr10_26770345_26759062_-11283-ABI1 | 22.8072 | 2.54E-14 | 6.68E-13 | up | chr10 | ABI1 |
| chr10_28131691_28119651_-12040-MPP7 | 6.099135 | 0.033051 | 0.196358 | up | chr10 | MPP7 |
| chr10_32584304_32543300_+41004-CCDC7 | 6.107539 | 0.032352 | 0.196358 | up | chr10 | CCDC7 |
| chr10_48410168_48401612_+8556-MAPK8 | 24.35075 | 6.12E-18 | 1.74E-15 | up | chr10 | MAPK8 |
| chr10_50590247_50433476_-156771-SGMS1 | 9.38343 | 0.000343 | 0.004548 | up | chr10 | SGMS1 |
| chr10_71732375_71730469_+1906-CDH23 | -21.6404 | 3.59E-13 | 6.56E-12 | down | chr10 | CDH23 |
| chr10_73479517_73467553_-11964-PPP3CB | -5.88546 | 0.048116 | 0.226783 | down | chr10 | PPP3CB |
| chr11_107392895_107390074_-2821-CWF19L2 | 7.079172 | 0.018031 | 0.147018 | up | chr11 | CWF19L2 |
| chr11_108235834_108227595_+8239-ATM | 8.533347 | 0.001892 | 0.023325 | up | chr11 | ATM |
| chr11_121060085_121045674_+14411-TBCEL | 7.322237 | 0.004826 | 0.051675 | up | chr11 | TBCEL |
| chr11_128490576_128480191_-10385-ETS1 | 7.231432 | 0.007127 | 0.071945 | up | chr11 | ETS1 |
| chr11_34970286_34947506_+22780-PDHX | -6.21606 | 0.036807 | 0.205343 | down | chr11 | PDHX |
| chr11_45872233_45862060_+10173-CRY2 | -9.06849 | 0.000597 | 0.007814 | down | chr11 | CRY2 |
| chr11_45979966_45965315_-14651-PHF21A | 22.29766 | 9.49E-14 | 2.19E-12 | up | chr11 | PHF21A |
| chr11_68190152_68166982_-23170-KMT5B | 21.43885 | 8.29E-13 | 1.31E-11 | up | chr11 | KMT5B |
| chr11_68564432_68551121_+13311-PPP6R3 | 8.777623 | 0.001747 | 0.022047 | up | chr11 | PPP6R3 |
| chr11_68603492_68596097_+7395-PPP6R3 | 6.632879 | 0.02673 | 0.183987 | up | chr11 | PPP6R3 |
| chr11_93700186_93696320_+3866-CEP295 | -20.6845 | 3.83E-12 | 5.56E-11 | down | chr11 | CEP295 |
| chr12_111683307_111679151_-4156-BRAP | -7.02858 | 0.009826 | 0.095554 | down | chr12 | BRAP |
| chr12_1190052_1115866_+74186-ERC1 | -5.97057 | 0.044956 | 0.220617 | down | chr12 | ERC1 |
| chr12_120155719_120154970_-749-GCN1 | 5.900595 | 0.048858 | 0.226783 | up | chr12 | GCN1 |
| chr12_120784593_120782655_-1938-SPPL3 | 6.584807 | 0.027747 | 0.188537 | up | chr12 | SPPL3 |
| chr12_1444750_1371833_+72917-ERC1 | 23.05673 | 1.32E-14 | 4.13E-13 | up | chr12 | ERC1 |
| chr12_26632059_26621123_-10936-ITPR2 | -5.8654 | 0.048729 | 0.226783 | down | chr12 | ITPR2 |
| chr12_28307749_28305649_+2100-CCDC91 | 7.829323 | 0.004537 | 0.049578 | up | chr12 | CCDC91 |
| chr12_28391411_28255581_+135830-CCDC91 | 22.17135 | 1.31E-13 | 2.83E-12 | up | chr12 | CCDC91 |
| chr12_42398994_42355161_+43833-na | 6.187599 | 0.038817 | 0.21319 | up | chr12 | na |
| chr12_5922804_5921040_-1764-ANO2 | 6.080488 | 0.042337 | 0.217368 | up | chr12 | ANO2 |
| chr12_66217235_66203711_+13524-IRAK3 | 9.52374 | 4.85E-05 | 0.000667 | up | chr12 | IRAK3 |
| chr12_66228370_66209456_+18914-IRAK3 | 6.159819 | 0.039705 | 0.21325 | up | chr12 | IRAK3 |
| chr12_79822190_79793863_-28327-PPP1R12A | -8.24291 | 0.004483 | 0.049496 | down | chr12 | PPP1R12A |
| chr13_28220378_28174272_+46106-PAN3 | -6.59882 | 0.026624 | 0.183987 | down | chr13 | PAN3 |
| chr13_30631474_30630835_+639-USPL1 | 6.442363 | 0.031432 | 0.195986 | up | chr13 | USPL1 |
| chr13_77243951_77205256_-38695-MYCBP2 | -6.92099 | 0.019825 | 0.156825 | down | chr13 | MYCBP2 |
| chr14_32094386_32090502_+3884-ARHGAP5 | 6.477121 | 0.030524 | 0.191455 | up | chr14 | ARHGAP5 |
| chr14_34802988_34800224_-2764-BAZ1A | 6.94089 | 0.020413 | 0.157944 | up | chr14 | BAZ1A |
| chr14_39327022_39313340_+13682-CTAGE5 | 6.47889 | 0.030479 | 0.191455 | up | chr14 | CTAGE5 |
| chr14_49831361_49799475_-31886-NEMF | -5.98663 | 0.04438 | 0.220617 | down | chr14 | NEMF |
| chr14_52544371_52536719_-7652-TXNDC16 | 5.682099 | 0.047924 | 0.226783 | up | chr14 | TXNDC16 |
| chr14_57247712_57229734_-17978-EXOC5 | 7.052474 | 0.01648 | 0.138965 | up | chr14 | EXOC5 |
| chr14_61457679_61443111_+14568-PRKCH | -7.88133 | 0.008104 | 0.080284 | down | chr14 | PRKCH |
| chr14_69122360_69116366_-5994-DCAF5 | 21.43885 | 8.29E-13 | 1.31E-11 | up | chr14 | DCAF5 |
| chr15_44660616_44600467_-60149-SPG11 | 6.327226 | 0.034605 | 0.200044 | up | chr15 | SPG11 |
| chr15_80122800_80120328_+2472-ZFAND6 | 6.642421 | 0.022924 | 0.167586 | up | chr15 | ZFAND6 |
| chr15_82833584_82826410_+7174-WHAMM | 22.32627 | 8.82E-14 | 2.08E-12 | up | chr15 | WHAMM |
| chr15_90221765_90217439_+4326-SEMA4B | 23.14806 | 1.04E-14 | 3.79E-13 | up | chr15 | SEMA4B |
| chr15_92956649_92937518_+19131-CHD2 | -6.28618 | 0.034725 | 0.200044 | down | chr15 | CHD2 |
| chr15_94458246_94440176_+18070-MCTP2 | 7.755467 | 0.005435 | 0.05704 | up | chr15 | MCTP2 |
| chr16_11779363_11774236_-5127-ZC3H7A | 6.26643 | 0.036388 | 0.205166 | up | chr16 | ZC3H7A |
| chr16_19647882_19616114_+31768-C16orf62 | -6.0988 | 0.02185 | 0.162159 | down | chr16 | C16orf62 |
| chr16_75414695_75411825_-2870-AC009163.4 | 23.63986 | 2.79E-15 | 1.99E-13 | up | chr16 | AC009163.4 |
| chr16_81908591_81895807_+12784-PLCG2 | -8.65297 | 0.003543 | 0.041267 | down | chr16 | PLCG2 |
| chr16_88611536_88598178_+13358-ZC3H18 | -21.6843 | 3.21E-13 | 6.08E-12 | down | chr16 | ZC3H18 |
| chr17_16101805_16101250_-555-NCOR1 | 6.737315 | 0.011692 | 0.105927 | up | chr17 | NCOR1 |
| chr17_37289528_37283267_-6261-ACACA | 21.60808 | 5.43E-13 | 9.14E-12 | up | chr17 | ACACA |
| chr17_40162901_40161753_+1148-CASC3 | 7.607065 | 0.011029 | 0.100778 | up | chr17 | CASC3 |
| chr17_49322508_49316826_-5682-ZNF652 | 5.956301 | 0.046754 | 0.225267 | up | chr17 | ZNF652 |
| chr17_50741731_50736960_+4771-LUC7L3 | 6.481185 | 0.03042 | 0.191455 | up | chr17 | LUC7L3 |
| chr17_56862251_56848696_+13555-DGKE | -21.8311 | 2.22E-13 | 4.27E-12 | down | chr17 | DGKE |
| chr17_61985090_61984171_-919-MED13 | 23.48857 | 4.19E-15 | 2.40E-13 | up | chr17 | MED13 |
| chr17_63766194_63760914_-5280-CCDC47 | 6.402491 | 0.031948 | 0.196358 | up | chr17 | CCDC47 |
| chr17_76304941_76287192_-17749-QRICH2 | 23.07022 | 1.27E-14 | 4.13E-13 | up | chr17 | QRICH2 |
| chr17_78087093_78079395_+7698-TNRC6C | 9.117006 | 8.31E-05 | 0.001129 | up | chr17 | TNRC6C |
| chr18_21044186_21039472_-4714-ROCK1 | 6.819031 | 0.019494 | 0.155364 | up | chr18 | ROCK1 |
| chr18_50940386_50918110_+22276-ME2 | 23.12853 | 1.09E-14 | 3.86E-13 | up | chr18 | ME2 |
| chr18_8143779_8113486_+30293-PTPRM | 21.16909 | 1.62E-12 | 2.41E-11 | up | chr18 | PTPRM |
| chr18_9221999_9208657_+13342-ANKRD12 | 23.39109 | 5.44E-15 | 2.40E-13 | up | chr18 | ANKRD12 |
| chr19_10177367_10163326_-14041-DNMT1 | 6.343059 | 0.034153 | 0.200044 | up | chr19 | DNMT1 |
| chr19_11514221_11513056_-1165-ECSIT | 24.10237 | 7.94E-16 | 9.35E-14 | up | chr19 | ECSIT |
| chr19_12528696_12517745_-10951-AC010422.6 | -24.378 | 7.31E-17 | 1.29E-14 | down | chr19 | AC010422.6 |
| chr19_18076327_18075749_-578-IL12RB1 | 6.499298 | 0.029957 | 0.191455 | up | chr19 | IL12RB1 |
| chr19_18147624_18147443_+181-MAST3 | -6.22762 | 0.016715 | 0.139509 | down | chr19 | MAST3 |
| chr19_18539720_18539371_-349-FKBP8 | -6.11994 | 0.039833 | 0.21325 | down | chr19 | FKBP8 |
| chr19_39453521_39453356_+165-SUPT5H | 23.472 | 4.38E-15 | 2.40E-13 | up | chr19 | SUPT5H |
| chr19_48913564_48913011_+553-NUCB1 | -8.30958 | 0.004125 | 0.047014 | down | chr19 | NUCB1 |
| chr19_5654456_5653115_+1341-SAFB | 6.523178 | 0.024674 | 0.176582 | up | chr19 | SAFB |
| chr19_8463686_8455405_+8281-HNRNPM | 7.547586 | 0.00635 | 0.065533 | up | chr19 | HNRNPM |
| chr2_101295181_101281859_-13322-RNF149 | 6.872168 | 0.020732 | 0.158099 | up | chr2 | RNF149 |
| chr2_101859864_101855977_+3887-MAP4K4 | 20.60639 | 6.08E-12 | 8.71E-11 | up | chr2 | MAP4K4 |
| chr2_113942359_113913172_+29187-ACTR3 | 6.166914 | 0.039476 | 0.21325 | up | chr2 | ACTR3 |
| chr2_135639122_135631718_+7404-R3HDM1 | -23.1243 | 7.67E-15 | 3.10E-13 | down | chr2 | R3HDM1 |
| chr2_135772547_135769769_+2778-UBXN4 | 7.438686 | 0.012723 | 0.113334 | up | chr2 | UBXN4 |
| chr2_152669270_152662603_-6667-PRPF40A | 23.75016 | 2.07E-15 | 1.99E-13 | up | chr2 | PRPF40A |
| chr2_168140758_168129541_-11217-STK39 | 5.997256 | 0.045256 | 0.221066 | up | chr2 | STK39 |
| chr2_168182090_168161787_-20303-STK39 | 23.27804 | 7.35E-15 | 3.10E-13 | up | chr2 | STK39 |
| chr2_173956232_173954873_-1359-SP3 | 21.67257 | 4.62E-13 | 8.17E-12 | up | chr2 | SP3 |
| chr2_201761818_201760881_-937-ALS2 | 6.643023 | 0.026498 | 0.183987 | up | chr2 | ALS2 |
| chr2_214792445_214752447_-39998-BARD1 | 23.43965 | 4.78E-15 | 2.40E-13 | up | chr2 | BARD1 |
| chr2_214797117_214767482_-29635-BARD1 | 6.26643 | 0.036388 | 0.205166 | up | chr2 | BARD1 |
| chr2_24147086_24135119_+11967-AC008073.3 | 7.113835 | 0.015832 | 0.136435 | up | chr2 | AC008073.3 |
| chr2_44218537_44209210_+9327-PPM1B | 23.40224 | 5.25E-15 | 2.40E-13 | up | chr2 | PPM1B |
| chr2_64553409_64551443_+1966-AFTPH | 5.911186 | 0.048452 | 0.226783 | up | chr2 | AFTPH |
| chr2_85371562_85368686_+2876-ELMOD3 | 6.186654 | 0.03566 | 0.203226 | up | chr2 | ELMOD3 |
| chr2_95153259_95148885_-4374-ZNF514 | -6.11994 | 0.039833 | 0.21325 | down | chr2 | ZNF514 |
| chr20_41101353_41092472_+8881-TOP1 | 6.926724 | 0.010008 | 0.096437 | up | chr20 | TOP1 |
| chr20_47633636_47623911_+9725-NCOA3 | 23.76449 | 1.99E-15 | 1.99E-13 | up | chr20 | NCOA3 |
| chr20_48963898_48953556_+10342-ARFGEF2 | 7.836249 | 0.001858 | 0.023175 | up | chr20 | ARFGEF2 |
| chr20_63931022_63928335_+2687-DNAJC5 | 7.613833 | 0.010201 | 0.097419 | up | chr20 | DNAJC5 |
| chr21_39212707_39206108_-6599-BRWD1 | 8.706825 | 0.000134 | 0.0018 | up | chr21 | BRWD1 |
| chr22_28846512_28844127_+2385-Z93930.2 | 23.05506 | 1.32E-14 | 4.13E-13 | up | chr22 | Z93930.2 |
| chr22_32485209_32478981_+6228-FBXO7 | 24.65033 | 1.91E-18 | 1.01E-15 | up | chr22 | FBXO7 |
| chr22_38248019_38245935_-2084-TMEM184B | 7.931216 | 0.007973 | 0.079725 | up | chr22 | TMEM184B |
| chr22_38501280_38499400_-1880-DDX17 | -20.3789 | 8.02E-12 | 1.12E-10 | down | chr22 | DDX17 |
| chr22_42215341_42209651_-5690-TCF20 | -21.8614 | 2.05E-13 | 4.04E-12 | down | chr22 | TCF20 |
| chr3_107733023_107710452_+22571-BBX | 21.61027 | 5.41E-13 | 9.14E-12 | up | chr3 | BBX |
| chr3_172251541_172112452_+139089-FNDC3B | 6.141259 | 0.040308 | 0.214705 | up | chr3 | FNDC3B |
| chr3_32737490_32733423_+4067-CNOT10 | 5.956301 | 0.046754 | 0.225267 | up | chr3 | CNOT10 |
| chr3_44840456_44829971_+10485-KIF15 | 6.886627 | 0.021421 | 0.161039 | up | chr3 | KIF15 |
| chr3_47428670_47425485_-3185-SCAP | 6.223546 | 0.012002 | 0.107813 | up | chr3 | SCAP |
| chr3_47636136_47635190_-946-SMARCC1 | 6.831633 | 0.022368 | 0.164656 | up | chr3 | SMARCC1 |
| chr3_52741499_52737586_-3913-NEK4 | 7.751389 | 0.004458 | 0.049496 | up | chr3 | NEK4 |
| chr3_56628614_56627037_-1577-FAM208A | 6.955237 | 0.020154 | 0.157362 | up | chr3 | FAM208A |
| chr3_71710484_71690010_-20474-EIF4E3 | 6.591671 | 0.027692 | 0.188537 | up | chr3 | EIF4E3 |
| chr4_102726683_102714438_-12245-MANBA | 6.017957 | 0.044129 | 0.220617 | up | chr4 | MANBA |
| chr4_118143684_118105018_+38666-NDST3 | 22.72142 | 3.18E-14 | 8.02E-13 | up | chr4 | NDST3 |
| chr4_150817257_150798081_-19176-LRBA | 6.862855 | 0.021876 | 0.162159 | up | chr4 | LRBA |
| chr4_1703700_1694774_-8926-SLBP | 22.84564 | 2.30E-14 | 6.24E-13 | up | chr4 | SLBP |
| chr4_254837_253560_+1277-ZNF876P | 6.011067 | 0.04476 | 0.220617 | up | chr4 | ZNF876P |
| chr4_3107423_3086939_+20484-HTT | 22.66318 | 3.70E-14 | 9.11E-13 | up | chr4 | HTT |
| chr4_39777810_39737420_+40390-UBE2K | 21.47256 | 7.62E-13 | 1.24E-11 | up | chr4 | UBE2K |
| chr4_39845880_39841948_-3932-PDS5A | 21.2164 | 1.44E-12 | 2.18E-11 | up | chr4 | PDS5A |
| chr4_41033304_41013583_-19721-APBB2 | 6.528336 | 0.029229 | 0.191455 | up | chr4 | APBB2 |
| chr4_53428183_53425872_+2311-AC058822.1 | 21.7312 | 3.99E-13 | 7.16E-12 | up | chr4 | AC058822.1 |
| chr4_78851056_78826037_+25019-BMP2K | 6.510876 | 0.029665 | 0.191455 | up | chr4 | BMP2K |
| chr4_82875822_82871944_-3878-SEC31A | 5.897064 | 0.048994 | 0.226783 | up | chr4 | SEC31A |
| chr4_87115299_87114367_+932-AFF1 | -7.13618 | 0.01648 | 0.138965 | down | chr4 | AFF1 |
| chr5_131510491_131504626_-5865-AC008695.1 | -6.20499 | 0.037145 | 0.206145 | down | chr5 | AC008695.1 |
| chr5_132893118_132892164_-954-AFF4 | 23.54178 | 3.63E-15 | 2.27E-13 | up | chr5 | AFF4 |
| chr5_138827718_138810038_+17680-CTNNA1 | 25.31232 | 1.45E-18 | 1.01E-15 | up | chr5 | CTNNA1 |
| chr5_154034967_154029489_-5478-FAM114A2 | 6.457824 | 0.01571 | 0.136435 | up | chr5 | FAM114A2 |
| chr5_157304366_157294783_+9583-CYFIP2 | 23.07801 | 1.25E-14 | 4.13E-13 | up | chr5 | CYFIP2 |
| chr5_172057473_172055588_-1885-STK10 | 6.735051 | 0.024181 | 0.175564 | up | chr5 | STK10 |
| chr5_172061268_172052929_-8339-STK10 | -3.5426 | 0.043988 | 0.220617 | down | chr5 | STK10 |
| chr5_177050936_177041126_+9810-ZNF346 | 24.12748 | 7.41E-16 | 9.35E-14 | up | chr5 | ZNF346 |
| chr5_179720560_179709873_+10687-CANX | 6.404445 | 0.032448 | 0.196358 | up | chr5 | CANX |
| chr5_39250806_39201826_-48980-FYB1 | 6.710906 | 0.024988 | 0.176582 | up | chr5 | FYB1 |
| chr5_50797233_50788523_+8710-PARP8 | -6.0418 | 0.042447 | 0.217368 | down | chr5 | PARP8 |
| chr5_66014725_65988635_+26090-ERBIN | 6.667885 | 0.025936 | 0.182067 | up | chr5 | ERBIN |
| chr5_71504751_71495250_+9501-BDP1 | -22.8341 | 1.66E-14 | 5.01E-13 | down | chr5 | BDP1 |
| chr5_77048272_77046347_+1925-AGGF1 | 7.726225 | 0.004969 | 0.052673 | up | chr5 | AGGF1 |
| chr5_77464809_77449760_-15049-WDR41 | 6.69178 | 0.017234 | 0.14272 | up | chr5 | WDR41 |
| chr5_87338091_87331348_+6743-RASA1 | 6.555724 | 0.028555 | 0.18918 | up | chr5 | RASA1 |
| chr5_94630743_94628811_+1932-SLF1 | 6.378524 | 0.033159 | 0.196358 | up | chr5 | SLF1 |
| chr5_94912976_94888879_-24097-MCTP1 | 8.560922 | 0.002216 | 0.026394 | up | chr5 | MCTP1 |
| chr5_97028612_97013648_+14964-LNPEP | -20.4537 | 6.70E-12 | 9.46E-11 | down | chr5 | LNPEP |
| chr6_130184623_130154825_-29798-SAMD3 | 7.089416 | 0.017718 | 0.145592 | up | chr6 | SAMD3 |
| chr6_13584225_13579451_+4774-SIRT5 | 8.463744 | 0.002117 | 0.0255 | up | chr6 | SIRT5 |
| chr6_144577288_144458770_+118518-UTRN | 6.488783 | 0.030225 | 0.191455 | up | chr6 | UTRN |
| chr6_145894977_145864388_-30589-SHPRH | 6.89297 | 0.021311 | 0.161039 | up | chr6 | SHPRH |
| chr6_158589782_158573420_+16362-TMEM181 | 6.505932 | 0.02979 | 0.191455 | up | chr6 | TMEM181 |
| chr6_167040339_167022409_+17930-FGFR1OP | -21.4894 | 5.25E-13 | 9.12E-12 | down | chr6 | FGFR1OP |
| chr6_57142095_57124734_+17361-ZNF451 | 7.967256 | 0.003725 | 0.042923 | up | chr6 | ZNF451 |
| chr6_75647801_75621532_+26269-SENP6 | 6.379839 | 0.033122 | 0.196358 | up | chr6 | SENP6 |
| chr6_84204096_84185187_-18909-CEP162 | 23.25051 | 7.91E-15 | 3.10E-13 | up | chr6 | CEP162 |
| chr7_100024307_100019231_+5076-ZKSCAN1 | 23.4328 | 4.87E-15 | 2.40E-13 | up | chr7 | ZKSCAN1 |
| chr7_105078963_105073619_+5344-KMT2E | -5.84358 | 0.049739 | 0.228787 | down | chr7 | KMT2E |
| chr7_10990847_10982372_+8475-PHF14 | 5.612877 | 0.041166 | 0.216162 | up | chr7 | PHF14 |
| chr7_111945798_111935540_-10258-DOCK4 | -5.88469 | 0.048145 | 0.226783 | down | chr7 | DOCK4 |
| chr7_116112038_116110708_-1330-TFEC | 6.850968 | 0.004228 | 0.047682 | up | chr7 | TFEC |
| chr7_131399433_131387120_+12313-MKLN1 | 6.075209 | 0.042517 | 0.217368 | up | chr7 | MKLN1 |
| chr7_139412580_139398599_+13981-LUC7L2 | 22.84943 | 2.27E-14 | 6.24E-13 | up | chr7 | LUC7L2 |
| chr7_139412580_139409563_+3017-LUC7L2 | -6.04812 | 0.042231 | 0.217368 | down | chr7 | LUC7L2 |
| chr7_152315338_152309966_-5372-KMT2C | -5.97951 | 0.040584 | 0.215095 | down | chr7 | KMT2C |
| chr7_155685052_155665175_+19877-RBM33 | -7.01183 | 0.018463 | 0.149393 | down | chr7 | RBM33 |
| chr7_22984045_22976210_-7835-FAM126A | 24.4602 | 2.96E-16 | 4.48E-14 | up | chr7 | FAM126A |
| chr7_24668660_24623666_+44994-MPP6 | 5.842545 | 0.034259 | 0.200044 | up | chr7 | MPP6 |
| chr7_27800090_27785163_+14927-TAX1BP1 | 6.247259 | 0.036591 | 0.205221 | up | chr7 | TAX1BP1 |
| chr7_66286709_66240325_+46384-TPST1 | -7.74917 | 0.009198 | 0.090276 | down | chr7 | TPST1 |
| chr7_77592256_77581427_+10829-PTPN12 | 21.99394 | 2.06E-13 | 4.04E-12 | up | chr7 | PTPN12 |
| chr8_100288267_100287501_-766-RNF19A | 7.65659 | 0.010516 | 0.098006 | up | chr8 | RNF19A |
| chr8_100713181_100709133_-4048-PABPC1 | 21.79371 | 3.41E-13 | 6.34E-12 | up | chr8 | PABPC1 |
| chr8_108455930_108449823_+6107-EMC2 | 6.334128 | 0.034407 | 0.200044 | up | chr8 | EMC2 |
| chr8_123339446_123337625_-1821-ATAD2 | 7.615863 | 0.010936 | 0.100778 | up | chr8 | ATAD2 |
| chr8_140890769_140846260_-44509-PTK2 | 22.80058 | 2.58E-14 | 6.68E-13 | up | chr8 | PTK2 |
| chr8_22475624_22474954_+670-PPP3CC | 21.4777 | 7.52E-13 | 1.24E-11 | up | chr8 | PPP3CC |
| chr8_47957431_47953620_-3811-PRKDC | 23.21271 | 8.74E-15 | 3.31E-13 | up | chr8 | PRKDC |
| chr8_60743097_60741259_+1838-CHD7 | 5.951411 | 0.046736 | 0.225267 | up | chr8 | CHD7 |
| chr8_61653660_61618978_-34682-ASPH | -5.89677 | 0.032042 | 0.196358 | down | chr8 | ASPH |
| chr8_66593194_66572482_-20712-MYBL1 | 7.654216 | 0.01054 | 0.098006 | up | chr8 | MYBL1 |
| chr8_67116122_67105905_+10217-CSPP1 | 24.56749 | 6.56E-18 | 1.74E-15 | up | chr8 | CSPP1 |
| chr8_67137603_67131951_+5652-CSPP1 | 21.31616 | 1.07E-12 | 1.65E-11 | up | chr8 | CSPP1 |
| chr8_674047_668598_-5449-ERICH1 | -22.0264 | 1.35E-13 | 2.86E-12 | down | chr8 | ERICH1 |
| chr8_68030658_68017846_+12812-PREX2 | 6.287893 | 0.035595 | 0.203226 | up | chr8 | PREX2 |
| chr8_99193057_99134632_+58425-VPS13B | 6.561662 | 0.028411 | 0.18918 | up | chr8 | VPS13B |
| chr9_112297916_112250929_-46987-PTBP3 | 21.36951 | 9.85E-13 | 1.54E-11 | up | chr9 | PTBP3 |
| chr9_137758011_137752331_+5680-EHMT1 | -6.03582 | 0.042653 | 0.217368 | down | chr9 | EHMT1 |
| chr9_137762820_137752331_+10489-EHMT1 | -5.99567 | 0.044058 | 0.220617 | down | chr9 | EHMT1 |
| chr9_14307520_14146689_-160831-NFIB | 6.037548 | 0.043822 | 0.220617 | up | chr9 | NFIB |
| chr9_16437524_16435555_-1969-BNC2 | -22.7909 | 1.84E-14 | 5.28E-13 | down | chr9 | BNC2 |
| chr9_33989126_33953285_-35841-UBAP2 | 22.01707 | 1.90E-13 | 3.87E-12 | up | chr9 | UBAP2 |
| chr9_37206494_37126312_+80182-ZCCHC7 | 7.659885 | 0.010361 | 0.098006 | up | chr9 | ZCCHC7 |
| chr9_37426654_37424845_+1809-GRHPR | 6.570078 | 0.028208 | 0.18918 | up | chr9 | GRHPR |
| chr9_77227485_77214329_+13156-VPS13A | 6.3987 | 0.012879 | 0.113763 | up | chr9 | VPS13A |
| chr9_96324169_96320998_-3171-SLC35D2 | -7.86911 | 0.00693 | 0.070635 | down | chr9 | SLC35D2 |
| chr9_96465778_96458379_+7399-HABP4 | -8.41514 | 0.004676 | 0.050579 | down | chr9 | HABP4 |
| chrX_110109146_110020352_+88794-TMEM164 | -22.0744 | 1.19E-13 | 2.63E-12 | down | chrX | TMEM164 |
| chrX_118546116_118542725_+3391-DOCK11 | 7.026101 | 0.018915 | 0.15189 | up | chrX | DOCK11 |
| chrX_131794466_131749306_-45160-FIRRE | 6.501881 | 0.029892 | 0.191455 | up | chrX | FIRRE |
| chrX_135556300_135545423_+10877-INTS6L | 22.11683 | 1.51E-13 | 3.13E-12 | up | chrX | INTS6L |
| chrX_140784659_140783175_+1484-na | 8.636326 | 0.00174 | 0.022047 | up | chrX | na |
| chrX_53615835_53614534_-1301-HUWE1 | 23.41872 | 5.05E-15 | 2.40E-13 | up | chrX | HUWE1 |
