# Supplementary material for: Expression Profiles and Functional Analysis of Plasma Exosomal Circular RNAs in Acute Myocardial Infarction
Source: Biomed Res Int. 2022 Oct 1;2022:3458227. doi: 10.1155/2022/3458227 (PMC9547997; doi:10.1155/2022/3458227)
Supplement: Supplementary 5 — Supplementary Table S5: Biological process enrichment analyses of the differentially expressed exosomal circRNAs in comparison of AMI and control. [file 3458227.f5.docx]

Supplementary Table S5 Biological process enrichment analyses of the differentially expressed exosomal circRNAs in comparison of AMI and control.

| GOID | GOTerm | DifGene | AllDifGene | GeneInGO | AllGene | P-Value | FDR | Enrichment | (-log10P) |
| --- | --- | --- | --- | --- | --- | --- | --- | --- | --- |
| GO:0016310 | phosphorylation | 26 | 209 | 785 | 16961 | 4.378E-06 | 0.0040254 | 2.6878737 | 5.3587234 |
| GO:0006355 | regulation of transcription, DNA-templated | 56 | 209 | 2550 | 16961 | 7.081E-06 | 0.0040254 | 1.7821859 | 5.1499179 |
| GO:0016568 | chromatin modification | 14 | 209 | 286 | 16961 | 1.293E-05 | 0.0049003 | 3.9725299 | 4.8884184 |
| GO:0006351 | transcription, DNA-templated | 52 | 209 | 2398 | 16961 | 2.408E-05 | 0.0068435 | 1.7597839 | 4.6184218 |
| GO:0006468 | protein phosphorylation | 21 | 209 | 638 | 16961 | 4.243E-05 | 0.0096488 | 2.6711839 | 4.372319 |
| GO:0030098 | lymphocyte differentiation | 3 | 209 | 8 | 16961 | 9.868E-05 | 0.0187003 | 30.432416 | 4.0057613 |
| GO:0035556 | intracellular signal transduction | 17 | 209 | 488 | 16961 | 0.0001191 | 0.019348 | 2.8270551 | 3.9240261 |
| GO:0051493 | regulation of cytoskeleton organization | 4 | 209 | 25 | 16961 | 0.0002313 | 0.0328734 | 12.984498 | 3.6358257 |
| GO:0034968 | histone lysine methylation | 4 | 209 | 29 | 16961 | 0.0004179 | 0.0497725 | 11.193532 | 3.3789767 |
| GO:0061470 | T follicular helper cell differentiation | 2 | 209 | 3 | 16961 | 0.0004497 | 0.0497725 | 54.102073 | 3.3470949 |
| GO:0000380 | alternative mRNA splicing, via spliceosome | 3 | 209 | 13 | 16961 | 0.0004815 | 0.0497725 | 18.727641 | 3.317378 |
| GO:0071044 | histone mRNA catabolic process | 3 | 209 | 14 | 16961 | 0.0006073 | 0.0575419 | 17.389952 | 3.2165947 |
| GO:0007010 | cytoskeleton organization | 7 | 209 | 122 | 16961 | 0.0007902 | 0.0691093 | 4.656326 | 3.1022805 |
| GO:0042159 | lipoprotein catabolic process | 2 | 209 | 4 | 16961 | 0.0008921 | 0.0717138 | 40.576555 | 3.0496098 |
| GO:0043547 | positive regulation of GTPase activity | 15 | 209 | 483 | 16961 | 0.0009834 | 0.0717138 | 2.5202829 | 3.0072772 |
| GO:0045893 | positive regulation of transcription, DNA-templated | 17 | 209 | 588 | 16961 | 0.0010092 | 0.0717138 | 2.3462634 | 2.9960375 |
| GO:0018107 | peptidyl-threonine phosphorylation | 4 | 209 | 37 | 16961 | 0.0010759 | 0.0719591 | 8.7733092 | 2.9682256 |
| GO:0033152 | immunoglobulin V(D)J recombination | 2 | 209 | 5 | 16961 | 0.0014747 | 0.0931506 | 32.461244 | 2.8313025 |
| GO:0051298 | centrosome duplication | 3 | 209 | 20 | 16961 | 0.0018011 | 0.1077835 | 12.172967 | 2.7444546 |
| GO:0018105 | peptidyl-serine phosphorylation | 6 | 209 | 105 | 16961 | 0.0019063 | 0.1083705 | 4.6373206 | 2.7198192 |
| GO:0045862 | positive regulation of proteolysis | 3 | 209 | 23 | 16961 | 0.0027231 | 0.1385686 | 10.585188 | 2.5649409 |
| GO:0035023 | regulation of Rho protein signal transduction | 5 | 209 | 79 | 16961 | 0.0029168 | 0.1385686 | 5.1362728 | 2.5351005 |
| GO:0032321 | positive regulation of Rho GTPase activity | 6 | 209 | 115 | 16961 | 0.0030123 | 0.1385686 | 4.2340753 | 2.5210986 |
| GO:0046826 | negative regulation of protein export from nucleus | 2 | 209 | 7 | 16961 | 0.0030468 | 0.1385686 | 23.186603 | 2.5161555 |
| GO:0043297 | apical junction assembly | 2 | 209 | 7 | 16961 | 0.0030468 | 0.1385686 | 23.186603 | 2.5161555 |
| GO:0001756 | somitogenesis | 4 | 209 | 51 | 16961 | 0.0035604 | 0.1556972 | 6.3649498 | 2.4485062 |
| GO:0051013 | microtubule severing | 2 | 209 | 8 | 16961 | 0.0040295 | 0.1696872 | 20.288278 | 2.3947476 |
| GO:0006325 | chromatin organization | 9 | 209 | 261 | 16961 | 0.0051684 | 0.1842021 | 2.7983831 | 2.286648 |
| GO:0043392 | negative regulation of DNA binding | 3 | 209 | 32 | 16961 | 0.0070315 | 0.1842021 | 7.6081041 | 2.1529497 |
| GO:0006338 | chromatin remodeling | 5 | 209 | 98 | 16961 | 0.0072885 | 0.1842021 | 4.1404648 | 2.1373606 |
| GO:0048010 | vascular endothelial growth factor receptor signaling pathway | 9 | 209 | 281 | 16961 | 0.0082352 | 0.1842021 | 2.5992099 | 2.0843247 |
| GO:0006397 | mRNA processing | 10 | 209 | 335 | 16961 | 0.0087946 | 0.1842021 | 2.4224809 | 2.055783 |
| GO:0042177 | negative regulation of protein catabolic process | 3 | 209 | 35 | 16961 | 0.0090316 | 0.1842021 | 6.9559809 | 2.0442357 |
| GO:0006590 | thyroid hormone generation | 2 | 209 | 12 | 16961 | 0.009195 | 0.1842021 | 13.525518 | 2.0364499 |
| GO:0043124 | negative regulation of I-kappaB kinase/NF-kappaB signaling | 3 | 209 | 36 | 16961 | 0.0097646 | 0.1842021 | 6.7627592 | 2.010347 |
| GO:0031122 | cytoplasmic microtubule organization | 3 | 209 | 36 | 16961 | 0.0097646 | 0.1842021 | 6.7627592 | 2.010347 |
| GO:0043473 | pigmentation | 3 | 209 | 36 | 16961 | 0.0097646 | 0.1842021 | 6.7627592 | 2.010347 |
| GO:0033151 | V(D)J recombination | 2 | 209 | 13 | 16961 | 0.0107792 | 0.1842021 | 12.485094 | 1.9674124 |
| GO:0071539 | protein localization to centrosome | 2 | 209 | 13 | 16961 | 0.0107792 | 0.1842021 | 12.485094 | 1.9674124 |
| GO:2000785 | regulation of autophagic vacuole assembly | 2 | 209 | 13 | 16961 | 0.0107792 | 0.1842021 | 12.485094 | 1.9674124 |
| GO:0032855 | positive regulation of Rac GTPase activity | 3 | 209 | 39 | 16961 | 0.0121666 | 0.1842021 | 6.2425469 | 1.9148313 |
| GO:2001229 | negative regulation of response to gamma radiation | 1 | 209 | 1 | 16961 | 0.0123224 | 0.1842021 | 81.15311 | 1.9093052 |
| GO:1901256 | regulation of macrophage colony-stimulating factor production | 1 | 209 | 1 | 16961 | 0.0123224 | 0.1842021 | 81.15311 | 1.9093052 |
| GO:0060567 | negative regulation of DNA-templated transcription, termination | 1 | 209 | 1 | 16961 | 0.0123224 | 0.1842021 | 81.15311 | 1.9093052 |
| GO:0036049 | peptidyl-lysine desuccinylation | 1 | 209 | 1 | 16961 | 0.0123224 | 0.1842021 | 81.15311 | 1.9093052 |
| GO:0036046 | protein demalonylation | 1 | 209 | 1 | 16961 | 0.0123224 | 0.1842021 | 81.15311 | 1.9093052 |
| GO:0036047 | peptidyl-lysine demalonylation | 1 | 209 | 1 | 16961 | 0.0123224 | 0.1842021 | 81.15311 | 1.9093052 |
| GO:0036048 | protein desuccinylation | 1 | 209 | 1 | 16961 | 0.0123224 | 0.1842021 | 81.15311 | 1.9093052 |
| GO:0098582 | innate vocalization behavior | 1 | 209 | 1 | 16961 | 0.0123224 | 0.1842021 | 81.15311 | 1.9093052 |
| GO:0010933 | positive regulation of macrophage tolerance induction | 1 | 209 | 1 | 16961 | 0.0123224 | 0.1842021 | 81.15311 | 1.9093052 |
| GO:0019319 | hexose biosynthetic process | 1 | 209 | 1 | 16961 | 0.0123224 | 0.1842021 | 81.15311 | 1.9093052 |
| GO:0031343 | positive regulation of cell killing | 1 | 209 | 1 | 16961 | 0.0123224 | 0.1842021 | 81.15311 | 1.9093052 |
| GO:0019477 | L-lysine catabolic process | 1 | 209 | 1 | 16961 | 0.0123224 | 0.1842021 | 81.15311 | 1.9093052 |
| GO:0000414 | regulation of histone H3-K36 methylation | 1 | 209 | 1 | 16961 | 0.0123224 | 0.1842021 | 81.15311 | 1.9093052 |
| GO:0033320 | UDP-D-xylose biosynthetic process | 1 | 209 | 1 | 16961 | 0.0123224 | 0.1842021 | 81.15311 | 1.9093052 |
| GO:0001770 | establishment of natural killer cell polarity | 1 | 209 | 1 | 16961 | 0.0123224 | 0.1842021 | 81.15311 | 1.9093052 |
| GO:1901340 | negative regulation of store-operated calcium channel activity | 1 | 209 | 1 | 16961 | 0.0123224 | 0.1842021 | 81.15311 | 1.9093052 |
| GO:0070265 | necrotic cell death | 1 | 209 | 1 | 16961 | 0.0123224 | 0.1842021 | 81.15311 | 1.9093052 |
| GO:0072431 | signal transduction involved in mitotic G1 DNA damage checkpoint | 1 | 209 | 1 | 16961 | 0.0123224 | 0.1842021 | 81.15311 | 1.9093052 |
| GO:0072434 | signal transduction involved in mitotic G2 DNA damage checkpoint | 1 | 209 | 1 | 16961 | 0.0123224 | 0.1842021 | 81.15311 | 1.9093052 |
| GO:0035048 | splicing factor protein import into nucleus | 1 | 209 | 1 | 16961 | 0.0123224 | 0.1842021 | 81.15311 | 1.9093052 |
| GO:1902044 | regulation of Fas signaling pathway | 1 | 209 | 1 | 16961 | 0.0123224 | 0.1842021 | 81.15311 | 1.9093052 |
| GO:2001182 | regulation of interleukin-12 secretion | 1 | 209 | 1 | 16961 | 0.0123224 | 0.1842021 | 81.15311 | 1.9093052 |
| GO:0010616 | negative regulation of cardiac muscle adaptation | 1 | 209 | 1 | 16961 | 0.0123224 | 0.1842021 | 81.15311 | 1.9093052 |
| GO:1900062 | regulation of replicative cell aging | 1 | 209 | 1 | 16961 | 0.0123224 | 0.1842021 | 81.15311 | 1.9093052 |
| GO:0021990 | neural plate formation | 1 | 209 | 1 | 16961 | 0.0123224 | 0.1842021 | 81.15311 | 1.9093052 |
| GO:0051341 | regulation of oxidoreductase activity | 1 | 209 | 1 | 16961 | 0.0123224 | 0.1842021 | 81.15311 | 1.9093052 |
| GO:0060051 | negative regulation of protein glycosylation | 1 | 209 | 1 | 16961 | 0.0123224 | 0.1842021 | 81.15311 | 1.9093052 |
| GO:0045660 | positive regulation of neutrophil differentiation | 1 | 209 | 1 | 16961 | 0.0123224 | 0.1842021 | 81.15311 | 1.9093052 |
| GO:0043244 | regulation of protein complex disassembly | 1 | 209 | 1 | 16961 | 0.0123224 | 0.1842021 | 81.15311 | 1.9093052 |
| GO:0031441 | negative regulation of mRNA 3'-end processing | 1 | 209 | 1 | 16961 | 0.0123224 | 0.1842021 | 81.15311 | 1.9093052 |
| GO:0035624 | receptor transactivation | 1 | 209 | 1 | 16961 | 0.0123224 | 0.1842021 | 81.15311 | 1.9093052 |
| GO:0010566 | regulation of ketone biosynthetic process | 1 | 209 | 1 | 16961 | 0.0123224 | 0.1842021 | 81.15311 | 1.9093052 |
| GO:0072619 | interleukin-21 secretion | 1 | 209 | 1 | 16961 | 0.0123224 | 0.1842021 | 81.15311 | 1.9093052 |
| GO:1900424 | regulation of defense response to bacterium | 1 | 209 | 1 | 16961 | 0.0123224 | 0.1842021 | 81.15311 | 1.9093052 |
| GO:0042182 | ketone catabolic process | 1 | 209 | 1 | 16961 | 0.0123224 | 0.1842021 | 81.15311 | 1.9093052 |
| GO:2000145 | regulation of cell motility | 2 | 209 | 14 | 16961 | 0.0124745 | 0.1842021 | 11.593301 | 1.9039752 |
| GO:0016573 | histone acetylation | 3 | 209 | 41 | 16961 | 0.0139396 | 0.1930405 | 5.9380324 | 1.8557504 |
| GO:0016477 | cell migration | 7 | 209 | 206 | 16961 | 0.0140632 | 0.1930405 | 2.75763 | 1.8519165 |
| GO:0051893 | regulation of focal adhesion assembly | 2 | 209 | 15 | 16961 | 0.014278 | 0.1930405 | 10.820415 | 1.8453333 |
| GO:0008340 | determination of adult lifespan | 2 | 209 | 15 | 16961 | 0.014278 | 0.1930405 | 10.820415 | 1.8453333 |
| GO:0047496 | vesicle transport along microtubule | 2 | 209 | 15 | 16961 | 0.014278 | 0.1930405 | 10.820415 | 1.8453333 |
| GO:0035855 | megakaryocyte development | 2 | 209 | 15 | 16961 | 0.014278 | 0.1930405 | 10.820415 | 1.8453333 |
| GO:0007018 | microtubule-based movement | 4 | 209 | 76 | 16961 | 0.014416 | 0.1930405 | 4.2712163 | 1.8411545 |
| GO:0038096 | Fc-gamma receptor signaling pathway involved in phagocytosis | 5 | 209 | 116 | 16961 | 0.0144314 | 0.1930405 | 3.4979789 | 1.840693 |
| GO:1903506 | regulation of nucleic acid-templated transcription | 3 | 209 | 43 | 16961 | 0.0158517 | 0.2008536 | 5.6618449 | 1.799925 |
| GO:0072358 | cardiovascular system development | 2 | 209 | 16 | 16961 | 0.0161866 | 0.2008536 | 10.144139 | 1.7908438 |
| GO:0051056 | regulation of small GTPase mediated signal transduction | 6 | 209 | 165 | 16961 | 0.016567 | 0.2008536 | 2.9510222 | 1.7807561 |
| GO:0006839 | mitochondrial transport | 2 | 209 | 17 | 16961 | 0.0181976 | 0.2008536 | 9.5474247 | 1.739985 |
| GO:0042445 | hormone metabolic process | 2 | 209 | 17 | 16961 | 0.0181976 | 0.2008536 | 9.5474247 | 1.739985 |
| GO:0043966 | histone H3 acetylation | 3 | 209 | 46 | 16961 | 0.0189829 | 0.2008536 | 5.2925941 | 1.7216366 |
| GO:0007173 | epidermal growth factor receptor signaling pathway | 9 | 209 | 324 | 16961 | 0.0193075 | 0.2008536 | 2.2542531 | 1.7142746 |
| GO:0007596 | blood coagulation | 12 | 209 | 496 | 16961 | 0.020269 | 0.2008536 | 1.9633817 | 1.6931671 |
| GO:0048535 | lymph node development | 2 | 209 | 18 | 16961 | 0.0203082 | 0.2008536 | 9.0170122 | 1.6923278 |
| GO:0031532 | actin cytoskeleton reorganization | 3 | 209 | 48 | 16961 | 0.0212468 | 0.2008536 | 5.0720694 | 1.6727056 |
| GO:0046777 | protein autophosphorylation | 6 | 209 | 175 | 16961 | 0.0214585 | 0.2008536 | 2.7823923 | 1.6684012 |
| GO:0042787 | protein ubiquitination involved in ubiquitin-dependent protein catabolic process | 4 | 209 | 86 | 16961 | 0.0217152 | 0.2008536 | 3.7745633 | 1.6632352 |
| GO:0048025 | negative regulation of mRNA splicing, via spliceosome | 2 | 209 | 19 | 16961 | 0.0225157 | 0.2008536 | 8.5424326 | 1.6475148 |
| GO:0070536 | protein K63-linked deubiquitination | 2 | 209 | 19 | 16961 | 0.0225157 | 0.2008536 | 8.5424326 | 1.6475148 |
| GO:0006376 | mRNA splice site selection | 2 | 209 | 19 | 16961 | 0.0225157 | 0.2008536 | 8.5424326 | 1.6475148 |
| GO:1901249 | regulation of lung goblet cell differentiation | 1 | 209 | 2 | 16961 | 0.0244936 | 0.2008536 | 40.576555 | 1.6109465 |
| GO:1901250 | negative regulation of lung goblet cell differentiation | 1 | 209 | 2 | 16961 | 0.0244936 | 0.2008536 | 40.576555 | 1.6109465 |
| GO:0071233 | cellular response to leucine | 1 | 209 | 2 | 16961 | 0.0244936 | 0.2008536 | 40.576555 | 1.6109465 |
| GO:0001172 | transcription, RNA-templated | 1 | 209 | 2 | 16961 | 0.0244936 | 0.2008536 | 40.576555 | 1.6109465 |
| GO:1903025 | regulation of RNA polymerase II regulatory region sequence-specific DNA binding | 1 | 209 | 2 | 16961 | 0.0244936 | 0.2008536 | 40.576555 | 1.6109465 |
| GO:0032510 | endosome to lysosome transport via multivesicular body sorting pathway | 1 | 209 | 2 | 16961 | 0.0244936 | 0.2008536 | 40.576555 | 1.6109465 |
| GO:0019244 | lactate biosynthetic process from pyruvate | 1 | 209 | 2 | 16961 | 0.0244936 | 0.2008536 | 40.576555 | 1.6109465 |
| GO:0010899 | regulation of phosphatidylcholine catabolic process | 1 | 209 | 2 | 16961 | 0.0244936 | 0.2008536 | 40.576555 | 1.6109465 |
| GO:0035926 | chemokine (C-C motif) ligand 2 secretion | 1 | 209 | 2 | 16961 | 0.0244936 | 0.2008536 | 40.576555 | 1.6109465 |
| GO:0043314 | negative regulation of neutrophil degranulation | 1 | 209 | 2 | 16961 | 0.0244936 | 0.2008536 | 40.576555 | 1.6109465 |
| GO:0043558 | regulation of translational initiation in response to stress | 1 | 209 | 2 | 16961 | 0.0244936 | 0.2008536 | 40.576555 | 1.6109465 |
| GO:2000629 | negative regulation of miRNA metabolic process | 1 | 209 | 2 | 16961 | 0.0244936 | 0.2008536 | 40.576555 | 1.6109465 |
| GO:2000620 | positive regulation of histone H4-K16 acetylation | 1 | 209 | 2 | 16961 | 0.0244936 | 0.2008536 | 40.576555 | 1.6109465 |
| GO:0002638 | negative regulation of immunoglobulin production | 1 | 209 | 2 | 16961 | 0.0244936 | 0.2008536 | 40.576555 | 1.6109465 |
| GO:0014870 | response to muscle inactivity | 1 | 209 | 2 | 16961 | 0.0244936 | 0.2008536 | 40.576555 | 1.6109465 |
| GO:0035305 | negative regulation of dephosphorylation | 1 | 209 | 2 | 16961 | 0.0244936 | 0.2008536 | 40.576555 | 1.6109465 |
| GO:2000401 | regulation of lymphocyte migration | 1 | 209 | 2 | 16961 | 0.0244936 | 0.2008536 | 40.576555 | 1.6109465 |
| GO:0033364 | mast cell secretory granule organization | 1 | 209 | 2 | 16961 | 0.0244936 | 0.2008536 | 40.576555 | 1.6109465 |
| GO:0002684 | positive regulation of immune system process | 1 | 209 | 2 | 16961 | 0.0244936 | 0.2008536 | 40.576555 | 1.6109465 |
| GO:0002692 | negative regulation of cellular extravasation | 1 | 209 | 2 | 16961 | 0.0244936 | 0.2008536 | 40.576555 | 1.6109465 |
| GO:0050706 | regulation of interleukin-1 beta secretion | 1 | 209 | 2 | 16961 | 0.0244936 | 0.2008536 | 40.576555 | 1.6109465 |
| GO:0002337 | B-1a B cell differentiation | 1 | 209 | 2 | 16961 | 0.0244936 | 0.2008536 | 40.576555 | 1.6109465 |
| GO:0071593 | lymphocyte aggregation | 1 | 209 | 2 | 16961 | 0.0244936 | 0.2008536 | 40.576555 | 1.6109465 |
| GO:1902031 | regulation of NADP metabolic process | 1 | 209 | 2 | 16961 | 0.0244936 | 0.2008536 | 40.576555 | 1.6109465 |
| GO:0021506 | anterior neuropore closure | 1 | 209 | 2 | 16961 | 0.0244936 | 0.2008536 | 40.576555 | 1.6109465 |
| GO:0097338 | response to clozapine | 1 | 209 | 2 | 16961 | 0.0244936 | 0.2008536 | 40.576555 | 1.6109465 |
| GO:0032635 | interleukin-6 production | 1 | 209 | 2 | 16961 | 0.0244936 | 0.2008536 | 40.576555 | 1.6109465 |
| GO:0061140 | lung secretory cell differentiation | 1 | 209 | 2 | 16961 | 0.0244936 | 0.2008536 | 40.576555 | 1.6109465 |
| GO:0045655 | regulation of monocyte differentiation | 1 | 209 | 2 | 16961 | 0.0244936 | 0.2008536 | 40.576555 | 1.6109465 |
| GO:0071955 | recycling endosome to Golgi transport | 1 | 209 | 2 | 16961 | 0.0244936 | 0.2008536 | 40.576555 | 1.6109465 |
| GO:0035617 | stress granule disassembly | 1 | 209 | 2 | 16961 | 0.0244936 | 0.2008536 | 40.576555 | 1.6109465 |
| GO:0021691 | cerebellar Purkinje cell layer maturation | 1 | 209 | 2 | 16961 | 0.0244936 | 0.2008536 | 40.576555 | 1.6109465 |
| GO:0051946 | regulation of glutamate uptake involved in transmission of nerve impulse | 1 | 209 | 2 | 16961 | 0.0244936 | 0.2008536 | 40.576555 | 1.6109465 |
| GO:2000791 | negative regulation of mesenchymal cell proliferation involved in lung development | 1 | 209 | 2 | 16961 | 0.0244936 | 0.2008536 | 40.576555 | 1.6109465 |
| GO:0035408 | histone H3-T6 phosphorylation | 1 | 209 | 2 | 16961 | 0.0244936 | 0.2008536 | 40.576555 | 1.6109465 |
| GO:0090241 | negative regulation of histone H4 acetylation | 1 | 209 | 2 | 16961 | 0.0244936 | 0.2008536 | 40.576555 | 1.6109465 |
| GO:0048753 | pigment granule organization | 1 | 209 | 2 | 16961 | 0.0244936 | 0.2008536 | 40.576555 | 1.6109465 |
| GO:2000584 | negative regulation of platelet-derived growth factor receptor-alpha signaling pathway | 1 | 209 | 2 | 16961 | 0.0244936 | 0.2008536 | 40.576555 | 1.6109465 |
| GO:0072583 | clathrin-mediated endocytosis | 2 | 209 | 20 | 16961 | 0.0248173 | 0.2008536 | 8.115311 | 1.6052454 |
| GO:0032318 | regulation of Ras GTPase activity | 2 | 209 | 20 | 16961 | 0.0248173 | 0.2008536 | 8.115311 | 1.6052454 |
| GO:0045727 | positive regulation of translation | 3 | 209 | 51 | 16961 | 0.024908 | 0.2008536 | 4.7737124 | 1.6036618 |
| GO:0035162 | embryonic hemopoiesis | 2 | 209 | 21 | 16961 | 0.0272105 | 0.2178753 | 7.7288676 | 1.5652642 |
| GO:0034047 | regulation of protein phosphatase type 2A activity | 2 | 209 | 22 | 16961 | 0.0296926 | 0.2306571 | 7.3775555 | 1.5273524 |
| GO:0070932 | histone H3 deacetylation | 2 | 209 | 22 | 16961 | 0.0296926 | 0.2306571 | 7.3775555 | 1.5273524 |
| GO:0006897 | endocytosis | 6 | 209 | 191 | 16961 | 0.0311713 | 0.2306571 | 2.5493124 | 1.5062457 |
| GO:0051568 | histone H3-K4 methylation | 2 | 209 | 23 | 16961 | 0.0322611 | 0.2306571 | 7.0567922 | 1.4913211 |
| GO:0008344 | adult locomotory behavior | 3 | 209 | 57 | 16961 | 0.0331822 | 0.2306571 | 4.2712163 | 1.4790953 |
| GO:0007519 | skeletal muscle tissue development | 3 | 209 | 58 | 16961 | 0.0346837 | 0.2306571 | 4.1975747 | 1.4598749 |
| GO:0030010 | establishment of cell polarity | 2 | 209 | 24 | 16961 | 0.0349135 | 0.2306571 | 6.7627592 | 1.4570061 |
| GO:0046060 | dATP metabolic process | 1 | 209 | 3 | 16961 | 0.0365156 | 0.2306571 | 27.051037 | 1.4375212 |
| GO:0060313 | negative regulation of blood vessel remodeling | 1 | 209 | 3 | 16961 | 0.0365156 | 0.2306571 | 27.051037 | 1.4375212 |
| GO:0060177 | regulation of angiotensin metabolic process | 1 | 209 | 3 | 16961 | 0.0365156 | 0.2306571 | 27.051037 | 1.4375212 |
| GO:0097676 | histone H3-K36 dimethylation | 1 | 209 | 3 | 16961 | 0.0365156 | 0.2306571 | 27.051037 | 1.4375212 |
| GO:0019249 | lactate biosynthetic process | 1 | 209 | 3 | 16961 | 0.0365156 | 0.2306571 | 27.051037 | 1.4375212 |
| GO:0031587 | positive regulation of inositol 1,4,5-trisphosphate-sensitive calcium-release channel activity | 1 | 209 | 3 | 16961 | 0.0365156 | 0.2306571 | 27.051037 | 1.4375212 |
| GO:0070508 | cholesterol import | 1 | 209 | 3 | 16961 | 0.0365156 | 0.2306571 | 27.051037 | 1.4375212 |
| GO:0090312 | positive regulation of protein deacetylation | 1 | 209 | 3 | 16961 | 0.0365156 | 0.2306571 | 27.051037 | 1.4375212 |
| GO:0033512 | L-lysine catabolic process to acetyl-CoA via saccharopine | 1 | 209 | 3 | 16961 | 0.0365156 | 0.2306571 | 27.051037 | 1.4375212 |
| GO:0035973 | aggrephagy | 1 | 209 | 3 | 16961 | 0.0365156 | 0.2306571 | 27.051037 | 1.4375212 |
| GO:0035523 | protein K29-linked deubiquitination | 1 | 209 | 3 | 16961 | 0.0365156 | 0.2306571 | 27.051037 | 1.4375212 |
| GO:0045586 | regulation of gamma-delta T cell differentiation | 1 | 209 | 3 | 16961 | 0.0365156 | 0.2306571 | 27.051037 | 1.4375212 |
| GO:1900024 | regulation of substrate adhesion-dependent cell spreading | 1 | 209 | 3 | 16961 | 0.0365156 | 0.2306571 | 27.051037 | 1.4375212 |
| GO:0000189 | MAPK import into nucleus | 1 | 209 | 3 | 16961 | 0.0365156 | 0.2306571 | 27.051037 | 1.4375212 |
| GO:0019836 | hemolysis by symbiont of host erythrocytes | 1 | 209 | 3 | 16961 | 0.0365156 | 0.2306571 | 27.051037 | 1.4375212 |
| GO:0071596 | ubiquitin-dependent protein catabolic process via the N-end rule pathway | 1 | 209 | 3 | 16961 | 0.0365156 | 0.2306571 | 27.051037 | 1.4375212 |
| GO:0010829 | negative regulation of glucose transport | 1 | 209 | 3 | 16961 | 0.0365156 | 0.2306571 | 27.051037 | 1.4375212 |
| GO:0019858 | cytosine metabolic process | 1 | 209 | 3 | 16961 | 0.0365156 | 0.2306571 | 27.051037 | 1.4375212 |
| GO:0089709 | L-histidine transmembrane transport | 1 | 209 | 3 | 16961 | 0.0365156 | 0.2306571 | 27.051037 | 1.4375212 |
| GO:1990168 | protein K33-linked deubiquitination | 1 | 209 | 3 | 16961 | 0.0365156 | 0.2306571 | 27.051037 | 1.4375212 |
| GO:0021988 | olfactory lobe development | 1 | 209 | 3 | 16961 | 0.0365156 | 0.2306571 | 27.051037 | 1.4375212 |
| GO:0034982 | mitochondrial protein processing | 1 | 209 | 3 | 16961 | 0.0365156 | 0.2306571 | 27.051037 | 1.4375212 |
| GO:0043243 | positive regulation of protein complex disassembly | 1 | 209 | 3 | 16961 | 0.0365156 | 0.2306571 | 27.051037 | 1.4375212 |
| GO:0033623 | regulation of integrin activation | 1 | 209 | 3 | 16961 | 0.0365156 | 0.2306571 | 27.051037 | 1.4375212 |
| GO:0018076 | N-terminal peptidyl-lysine acetylation | 1 | 209 | 3 | 16961 | 0.0365156 | 0.2306571 | 27.051037 | 1.4375212 |
| GO:0033260 | nuclear DNA replication | 1 | 209 | 3 | 16961 | 0.0365156 | 0.2306571 | 27.051037 | 1.4375212 |
| GO:0014737 | positive regulation of muscle atrophy | 1 | 209 | 3 | 16961 | 0.0365156 | 0.2306571 | 27.051037 | 1.4375212 |
| GO:1901509 | regulation of endothelial tube morphogenesis | 1 | 209 | 3 | 16961 | 0.0365156 | 0.2306571 | 27.051037 | 1.4375212 |
| GO:0015817 | histidine transport | 1 | 209 | 3 | 16961 | 0.0365156 | 0.2306571 | 27.051037 | 1.4375212 |
| GO:0034140 | negative regulation of toll-like receptor 3 signaling pathway | 1 | 209 | 3 | 16961 | 0.0365156 | 0.2306571 | 27.051037 | 1.4375212 |
| GO:0002331 | pre-B cell allelic exclusion | 1 | 209 | 3 | 16961 | 0.0365156 | 0.2306571 | 27.051037 | 1.4375212 |
| GO:0007264 | small GTPase mediated signal transduction | 15 | 209 | 732 | 16961 | 0.0376133 | 0.233134 | 1.6629736 | 1.4246582 |
| GO:0016571 | histone methylation | 2 | 209 | 25 | 16961 | 0.0376475 | 0.233134 | 6.4922488 | 1.4242639 |
| GO:0008045 | motor neuron axon guidance | 2 | 209 | 25 | 16961 | 0.0376475 | 0.233134 | 6.4922488 | 1.4242639 |
| GO:0008380 | RNA splicing | 8 | 209 | 311 | 16961 | 0.0392423 | 0.233134 | 2.0875398 | 1.4062454 |
| GO:0030316 | osteoclast differentiation | 2 | 209 | 26 | 16961 | 0.0404606 | 0.233134 | 6.2425469 | 1.3929682 |
| GO:0071549 | cellular response to dexamethasone stimulus | 2 | 209 | 27 | 16961 | 0.0433504 | 0.233134 | 6.0113415 | 1.3630071 |
| GO:0007623 | circadian rhythm | 4 | 209 | 108 | 16961 | 0.0446084 | 0.233134 | 3.0056707 | 1.3505833 |
| GO:0051881 | regulation of mitochondrial membrane potential | 2 | 209 | 28 | 16961 | 0.0463147 | 0.233134 | 5.7966507 | 1.3342815 |
| GO:0007052 | mitotic spindle organization | 2 | 209 | 28 | 16961 | 0.0463147 | 0.233134 | 5.7966507 | 1.3342815 |
| GO:0034587 | piRNA metabolic process | 2 | 209 | 28 | 16961 | 0.0463147 | 0.233134 | 5.7966507 | 1.3342815 |
| GO:0006366 | transcription from RNA polymerase II promoter | 14 | 209 | 692 | 16961 | 0.0477211 | 0.233134 | 1.6418259 | 1.3212894 |
| GO:2000117 | negative regulation of cysteine-type endopeptidase activity | 1 | 209 | 4 | 16961 | 0.0483902 | 0.233134 | 20.288278 | 1.3152429 |
| GO:0000052 | citrulline metabolic process | 1 | 209 | 4 | 16961 | 0.0483902 | 0.233134 | 20.288278 | 1.3152429 |
| GO:0003383 | apical constriction | 1 | 209 | 4 | 16961 | 0.0483902 | 0.233134 | 20.288278 | 1.3152429 |
| GO:0006857 | oligopeptide transport | 1 | 209 | 4 | 16961 | 0.0483902 | 0.233134 | 20.288278 | 1.3152429 |
| GO:0036091 | positive regulation of transcription from RNA polymerase II promoter in response to oxidative stress | 1 | 209 | 4 | 16961 | 0.0483902 | 0.233134 | 20.288278 | 1.3152429 |
| GO:0051252 | regulation of RNA metabolic process | 1 | 209 | 4 | 16961 | 0.0483902 | 0.233134 | 20.288278 | 1.3152429 |
| GO:0016241 | regulation of macroautophagy | 1 | 209 | 4 | 16961 | 0.0483902 | 0.233134 | 20.288278 | 1.3152429 |
| GO:1902175 | regulation of oxidative stress-induced intrinsic apoptotic signaling pathway | 1 | 209 | 4 | 16961 | 0.0483902 | 0.233134 | 20.288278 | 1.3152429 |
| GO:1901097 | negative regulation of autophagic vacuole maturation | 1 | 209 | 4 | 16961 | 0.0483902 | 0.233134 | 20.288278 | 1.3152429 |
| GO:0042985 | negative regulation of amyloid precursor protein biosynthetic process | 1 | 209 | 4 | 16961 | 0.0483902 | 0.233134 | 20.288278 | 1.3152429 |
| GO:0006499 | N-terminal protein myristoylation | 1 | 209 | 4 | 16961 | 0.0483902 | 0.233134 | 20.288278 | 1.3152429 |
| GO:0042998 | positive regulation of Golgi to plasma membrane protein transport | 1 | 209 | 4 | 16961 | 0.0483902 | 0.233134 | 20.288278 | 1.3152429 |
| GO:0032970 | regulation of actin filament-based process | 1 | 209 | 4 | 16961 | 0.0483902 | 0.233134 | 20.288278 | 1.3152429 |
| GO:0010961 | cellular magnesium ion homeostasis | 1 | 209 | 4 | 16961 | 0.0483902 | 0.233134 | 20.288278 | 1.3152429 |
| GO:0007028 | cytoplasm organization | 1 | 209 | 4 | 16961 | 0.0483902 | 0.233134 | 20.288278 | 1.3152429 |
| GO:0033522 | histone H2A ubiquitination | 1 | 209 | 4 | 16961 | 0.0483902 | 0.233134 | 20.288278 | 1.3152429 |
| GO:0071800 | podosome assembly | 1 | 209 | 4 | 16961 | 0.0483902 | 0.233134 | 20.288278 | 1.3152429 |
| GO:0001920 | negative regulation of receptor recycling | 1 | 209 | 4 | 16961 | 0.0483902 | 0.233134 | 20.288278 | 1.3152429 |
| GO:0097167 | circadian regulation of translation | 1 | 209 | 4 | 16961 | 0.0483902 | 0.233134 | 20.288278 | 1.3152429 |
| GO:0001754 | eye photoreceptor cell differentiation | 1 | 209 | 4 | 16961 | 0.0483902 | 0.233134 | 20.288278 | 1.3152429 |
| GO:0035984 | cellular response to trichostatin A | 1 | 209 | 4 | 16961 | 0.0483902 | 0.233134 | 20.288278 | 1.3152429 |
| GO:0090170 | regulation of Golgi inheritance | 1 | 209 | 4 | 16961 | 0.0483902 | 0.233134 | 20.288278 | 1.3152429 |
| GO:0033136 | serine phosphorylation of STAT3 protein | 1 | 209 | 4 | 16961 | 0.0483902 | 0.233134 | 20.288278 | 1.3152429 |
| GO:0034454 | microtubule anchoring at centrosome | 1 | 209 | 4 | 16961 | 0.0483902 | 0.233134 | 20.288278 | 1.3152429 |
| GO:0060713 | labyrinthine layer morphogenesis | 1 | 209 | 4 | 16961 | 0.0483902 | 0.233134 | 20.288278 | 1.3152429 |
| GO:0044027 | hypermethylation of CpG island | 1 | 209 | 4 | 16961 | 0.0483902 | 0.233134 | 20.288278 | 1.3152429 |
| GO:0023051 | regulation of signaling | 1 | 209 | 4 | 16961 | 0.0483902 | 0.233134 | 20.288278 | 1.3152429 |
| GO:0071389 | cellular response to mineralocorticoid stimulus | 1 | 209 | 4 | 16961 | 0.0483902 | 0.233134 | 20.288278 | 1.3152429 |
| GO:0071322 | cellular response to carbohydrate stimulus | 1 | 209 | 4 | 16961 | 0.0483902 | 0.233134 | 20.288278 | 1.3152429 |
| GO:0072201 | negative regulation of mesenchymal cell proliferation | 1 | 209 | 4 | 16961 | 0.0483902 | 0.233134 | 20.288278 | 1.3152429 |
| GO:0031936 | negative regulation of chromatin silencing | 1 | 209 | 4 | 16961 | 0.0483902 | 0.233134 | 20.288278 | 1.3152429 |
| GO:0019805 | quinolinate biosynthetic process | 1 | 209 | 4 | 16961 | 0.0483902 | 0.233134 | 20.288278 | 1.3152429 |
| GO:0010810 | regulation of cell-substrate adhesion | 1 | 209 | 4 | 16961 | 0.0483902 | 0.233134 | 20.288278 | 1.3152429 |
| GO:0038127 | ERBB signaling pathway | 1 | 209 | 4 | 16961 | 0.0483902 | 0.233134 | 20.288278 | 1.3152429 |
| GO:0060043 | regulation of cardiac muscle cell proliferation | 1 | 209 | 4 | 16961 | 0.0483902 | 0.233134 | 20.288278 | 1.3152429 |
| GO:1903347 | negative regulation of tight junction assembly | 1 | 209 | 4 | 16961 | 0.0483902 | 0.233134 | 20.288278 | 1.3152429 |
| GO:0043242 | negative regulation of protein complex disassembly | 1 | 209 | 4 | 16961 | 0.0483902 | 0.233134 | 20.288278 | 1.3152429 |
| GO:0010998 | regulation of translational initiation by eIF2 alpha phosphorylation | 1 | 209 | 4 | 16961 | 0.0483902 | 0.233134 | 20.288278 | 1.3152429 |
| GO:0071947 | protein deubiquitination involved in ubiquitin-dependent protein catabolic process | 1 | 209 | 4 | 16961 | 0.0483902 | 0.233134 | 20.288278 | 1.3152429 |
| GO:0090207 | regulation of triglyceride metabolic process | 1 | 209 | 4 | 16961 | 0.0483902 | 0.233134 | 20.288278 | 1.3152429 |
| GO:0046952 | ketone body catabolic process | 1 | 209 | 4 | 16961 | 0.0483902 | 0.233134 | 20.288278 | 1.3152429 |
| GO:0070816 | phosphorylation of RNA polymerase II C-terminal domain | 1 | 209 | 4 | 16961 | 0.0483902 | 0.233134 | 20.288278 | 1.3152429 |
| GO:0034340 | response to type I interferon | 1 | 209 | 4 | 16961 | 0.0483902 | 0.233134 | 20.288278 | 1.3152429 |
| GO:0033299 | secretion of lysosomal enzymes | 1 | 209 | 4 | 16961 | 0.0483902 | 0.233134 | 20.288278 | 1.3152429 |
| GO:0002329 | pre-B cell differentiation | 1 | 209 | 4 | 16961 | 0.0483902 | 0.233134 | 20.288278 | 1.3152429 |
