# Supplementary material for: Expression Profiles and Functional Analysis of Plasma Exosomal Circular RNAs in Acute Myocardial Infarction
Source: Biomed Res Int. 2022 Oct 1;2022:3458227. doi: 10.1155/2022/3458227 (PMC9547997; doi:10.1155/2022/3458227)
Supplement: Supplementary 6 — Supplementary Table S6: Biological process enrichment analyses of the differentially expressed exosomal circRNAs in comparison of CAD and control. [file 3458227.f6.docx]

Supplementary Table S6 Biological process enrichment analyses of the differentially expressed exosomal circRNAs in comparison of CAD and control.

| GOID | GOTerm | DifGene | AllDifGene | GeneInGO | AllGene | P-Value | FDR | Enrichment | (-log10P) |
| --- | --- | --- | --- | --- | --- | --- | --- | --- | --- |
| GO:0016568 | chromatin modification | 15 | 156 | 286 | 16961 | 6.286E-08 | 6.745E-05 | 5.7023265 | 7.2016449 |
| GO:0006355 | regulation of transcription, DNA-templated | 48 | 156 | 2550 | 16961 | 4.627E-07 | 0.0002483 | 2.0465762 | 6.3346711 |
| GO:0006306 | DNA methylation | 5 | 156 | 26 | 16961 | 3.474E-06 | 0.0012424 | 20.908531 | 5.4592172 |
| GO:0034605 | cellular response to heat | 8 | 156 | 103 | 16961 | 4.857E-06 | 0.0013028 | 8.4446104 | 5.3136482 |
| GO:0006351 | transcription, DNA-templated | 43 | 156 | 2398 | 16961 | 8.242E-06 | 0.0017687 | 1.9496028 | 5.0839641 |
| GO:0050861 | positive regulation of B cell receptor signaling pathway | 3 | 156 | 6 | 16961 | 1.496E-05 | 0.0026751 | 54.362179 | 4.8251131 |
| GO:0045893 | positive regulation of transcription, DNA-templated | 17 | 156 | 588 | 16961 | 2.995E-05 | 0.004591 | 3.1433913 | 4.5235981 |
| GO:1900034 | regulation of cellular response to heat | 6 | 156 | 70 | 16961 | 4.436E-05 | 0.0059499 | 9.3192308 | 4.3529968 |
| GO:0006325 | chromatin organization | 10 | 156 | 261 | 16961 | 0.0001535 | 0.0182975 | 4.1656843 | 3.8139661 |
| GO:0030168 | platelet activation | 9 | 156 | 216 | 16961 | 0.0001766 | 0.0189487 | 4.5301816 | 3.7530198 |
| GO:0007173 | epidermal growth factor receptor signaling pathway | 11 | 156 | 324 | 16961 | 0.0002074 | 0.0202291 | 3.6912591 | 3.6832307 |
| GO:0006338 | chromatin remodeling | 6 | 156 | 98 | 16961 | 0.0002883 | 0.0238317 | 6.6565934 | 3.5400936 |
| GO:0050853 | B cell receptor signaling pathway | 4 | 156 | 35 | 16961 | 0.0002887 | 0.0238317 | 12.425641 | 3.5395021 |
| GO:0030335 | positive regulation of cell migration | 7 | 156 | 162 | 16961 | 0.0007545 | 0.0520984 | 4.6979661 | 3.1223285 |
| GO:0032237 | activation of store-operated calcium channel activity | 2 | 156 | 5 | 16961 | 0.0008254 | 0.0520984 | 43.489744 | 3.0833267 |
| GO:0090188 | negative regulation of pancreatic juice secretion | 2 | 156 | 5 | 16961 | 0.0008254 | 0.0520984 | 43.489744 | 3.0833267 |
| GO:0060964 | regulation of gene silencing by miRNA | 2 | 156 | 5 | 16961 | 0.0008254 | 0.0520984 | 43.489744 | 3.0833267 |
| GO:0071277 | cellular response to calcium ion | 4 | 156 | 48 | 16961 | 0.000978 | 0.0574123 | 9.0603632 | 3.0096411 |
| GO:0008361 | regulation of cell size | 3 | 156 | 23 | 16961 | 0.0011808 | 0.0574123 | 14.181438 | 2.9278252 |
| GO:0045862 | positive regulation of proteolysis | 3 | 156 | 23 | 16961 | 0.0011808 | 0.0574123 | 14.181438 | 2.9278252 |
| GO:0045727 | positive regulation of translation | 4 | 156 | 51 | 16961 | 0.0012296 | 0.0574123 | 8.5274007 | 2.910238 |
| GO:0010649 | regulation of cell communication by electrical coupling | 2 | 156 | 6 | 16961 | 0.0012306 | 0.0574123 | 36.241453 | 2.9098669 |
| GO:0010216 | maintenance of DNA methylation | 2 | 156 | 6 | 16961 | 0.0012306 | 0.0574123 | 36.241453 | 2.9098669 |
| GO:0046826 | negative regulation of protein export from nucleus | 2 | 156 | 7 | 16961 | 0.0017125 | 0.0765632 | 31.064103 | 2.7663684 |
| GO:0016441 | posttranscriptional gene silencing | 2 | 156 | 8 | 16961 | 0.0022696 | 0.0885708 | 27.18109 | 2.6440572 |
| GO:0016584 | nucleosome positioning | 2 | 156 | 8 | 16961 | 0.0022696 | 0.0885708 | 27.18109 | 2.6440572 |
| GO:0006468 | protein phosphorylation | 14 | 156 | 638 | 16961 | 0.0023051 | 0.0885708 | 2.385801 | 2.6373155 |
| GO:0034968 | histone lysine methylation | 3 | 156 | 29 | 16961 | 0.0023399 | 0.0885708 | 11.247347 | 2.6308016 |
| GO:0016310 | phosphorylation | 16 | 156 | 785 | 16961 | 0.0023938 | 0.0885708 | 2.2160379 | 2.6209111 |
| GO:0045765 | regulation of angiogenesis | 3 | 156 | 31 | 16961 | 0.0028401 | 0.1015795 | 10.521712 | 2.5466725 |
| GO:0043392 | negative regulation of DNA binding | 3 | 156 | 32 | 16961 | 0.0031129 | 0.1049893 | 10.192909 | 2.5068346 |
| GO:0060252 | positive regulation of glial cell proliferation | 2 | 156 | 10 | 16961 | 0.0036037 | 0.1049893 | 21.744872 | 2.4432522 |
| GO:1990138 | neuron projection extension | 2 | 156 | 10 | 16961 | 0.0036037 | 0.1049893 | 21.744872 | 2.4432522 |
| GO:0016032 | viral process | 13 | 156 | 601 | 16961 | 0.0037156 | 0.1049893 | 2.3517748 | 2.4299698 |
| GO:0032321 | positive regulation of Rho GTPase activity | 5 | 156 | 115 | 16961 | 0.0042087 | 0.1049893 | 4.727146 | 2.3758516 |
| GO:0051272 | positive regulation of cellular component movement | 2 | 156 | 11 | 16961 | 0.004378 | 0.1049893 | 19.768065 | 2.3587239 |
| GO:0090161 | Golgi ribbon formation | 2 | 156 | 11 | 16961 | 0.004378 | 0.1049893 | 19.768065 | 2.3587239 |
| GO:0071456 | cellular response to hypoxia | 5 | 156 | 118 | 16961 | 0.0046941 | 0.1049893 | 4.6069644 | 2.3284505 |
| GO:0018107 | peptidyl-threonine phosphorylation | 3 | 156 | 37 | 16961 | 0.0047158 | 0.1049893 | 8.8154886 | 2.3264468 |
| GO:0010941 | regulation of cell death | 2 | 156 | 12 | 16961 | 0.005222 | 0.1049893 | 18.120726 | 2.2821626 |
| GO:0045945 | positive regulation of transcription from RNA polymerase III promoter | 2 | 156 | 12 | 16961 | 0.005222 | 0.1049893 | 18.120726 | 2.2821626 |
| GO:0006590 | thyroid hormone generation | 2 | 156 | 12 | 16961 | 0.005222 | 0.1049893 | 18.120726 | 2.2821626 |
| GO:0048193 | Golgi vesicle transport | 2 | 156 | 12 | 16961 | 0.005222 | 0.1049893 | 18.120726 | 2.2821626 |
| GO:0043268 | positive regulation of potassium ion transport | 2 | 156 | 12 | 16961 | 0.005222 | 0.1049893 | 18.120726 | 2.2821626 |
| GO:0035556 | intracellular signal transduction | 11 | 156 | 488 | 16961 | 0.0055188 | 0.1049893 | 2.450754 | 2.2581574 |
| GO:0006461 | protein complex assembly | 5 | 156 | 123 | 16961 | 0.0055895 | 0.1049893 | 4.4196894 | 2.2526292 |
| GO:0008543 | fibroblast growth factor receptor signaling pathway | 8 | 156 | 292 | 16961 | 0.0056721 | 0.1049893 | 2.9787496 | 2.2462534 |
| GO:0002446 | neutrophil mediated immunity | 2 | 156 | 13 | 16961 | 0.0061344 | 0.1049893 | 16.726824 | 2.21223 |
| GO:2000785 | regulation of autophagic vacuole assembly | 2 | 156 | 13 | 16961 | 0.0061344 | 0.1049893 | 16.726824 | 2.21223 |
| GO:0007596 | blood coagulation | 11 | 156 | 496 | 16961 | 0.0062124 | 0.1049893 | 2.4112257 | 2.2067375 |
| GO:0071044 | histone mRNA catabolic process | 2 | 156 | 14 | 16961 | 0.0071138 | 0.1049893 | 15.532051 | 2.1478993 |
| GO:0045055 | regulated secretory pathway | 2 | 156 | 14 | 16961 | 0.0071138 | 0.1049893 | 15.532051 | 2.1478993 |
| GO:0010524 | positive regulation of calcium ion transport into cytosol | 2 | 156 | 15 | 16961 | 0.008159 | 0.1049893 | 14.496581 | 2.0883656 |
| GO:0001817 | regulation of cytokine production | 2 | 156 | 15 | 16961 | 0.008159 | 0.1049893 | 14.496581 | 2.0883656 |
| GO:2000151 | negative regulation of planar cell polarity pathway involved in cardiac muscle tissue morphogenesis | 1 | 156 | 1 | 16961 | 0.0091976 | 0.1049893 | 108.72436 | 2.0363269 |
| GO:2000149 | negative regulation of planar cell polarity pathway involved in ventricular septum morphogenesis | 1 | 156 | 1 | 16961 | 0.0091976 | 0.1049893 | 108.72436 | 2.0363269 |
| GO:2000164 | negative regulation of planar cell polarity pathway involved in outflow tract morphogenesis | 1 | 156 | 1 | 16961 | 0.0091976 | 0.1049893 | 108.72436 | 2.0363269 |
| GO:2000162 | negative regulation of planar cell polarity pathway involved in cardiac right atrium morphogenesis | 1 | 156 | 1 | 16961 | 0.0091976 | 0.1049893 | 108.72436 | 2.0363269 |
| GO:2000168 | negative regulation of planar cell polarity pathway involved in neural tube closure | 1 | 156 | 1 | 16961 | 0.0091976 | 0.1049893 | 108.72436 | 2.0363269 |
| GO:2000166 | negative regulation of planar cell polarity pathway involved in pericardium morphogenesis | 1 | 156 | 1 | 16961 | 0.0091976 | 0.1049893 | 108.72436 | 2.0363269 |
| GO:0014029 | neural crest formation | 1 | 156 | 1 | 16961 | 0.0091976 | 0.1049893 | 108.72436 | 2.0363269 |
| GO:0031343 | positive regulation of cell killing | 1 | 156 | 1 | 16961 | 0.0091976 | 0.1049893 | 108.72436 | 2.0363269 |
| GO:0019477 | L-lysine catabolic process | 1 | 156 | 1 | 16961 | 0.0091976 | 0.1049893 | 108.72436 | 2.0363269 |
| GO:0097198 | histone H3-K36 trimethylation | 1 | 156 | 1 | 16961 | 0.0091976 | 0.1049893 | 108.72436 | 2.0363269 |
| GO:0035508 | positive regulation of myosin-light-chain-phosphatase activity | 1 | 156 | 1 | 16961 | 0.0091976 | 0.1049893 | 108.72436 | 2.0363269 |
| GO:1901879 | regulation of protein depolymerization | 1 | 156 | 1 | 16961 | 0.0091976 | 0.1049893 | 108.72436 | 2.0363269 |
| GO:0014866 | skeletal myofibril assembly | 1 | 156 | 1 | 16961 | 0.0091976 | 0.1049893 | 108.72436 | 2.0363269 |
| GO:0001770 | establishment of natural killer cell polarity | 1 | 156 | 1 | 16961 | 0.0091976 | 0.1049893 | 108.72436 | 2.0363269 |
| GO:2000209 | regulation of anoikis | 1 | 156 | 1 | 16961 | 0.0091976 | 0.1049893 | 108.72436 | 2.0363269 |
| GO:0070304 | positive regulation of stress-activated protein kinase signaling cascade | 1 | 156 | 1 | 16961 | 0.0091976 | 0.1049893 | 108.72436 | 2.0363269 |
| GO:0071622 | regulation of granulocyte chemotaxis | 1 | 156 | 1 | 16961 | 0.0091976 | 0.1049893 | 108.72436 | 2.0363269 |
| GO:1901340 | negative regulation of store-operated calcium channel activity | 1 | 156 | 1 | 16961 | 0.0091976 | 0.1049893 | 108.72436 | 2.0363269 |
| GO:1903533 | regulation of protein targeting | 1 | 156 | 1 | 16961 | 0.0091976 | 0.1049893 | 108.72436 | 2.0363269 |
| GO:0061310 | canonical Wnt signaling pathway involved in cardiac neural crest cell differentiation involved in heart development | 1 | 156 | 1 | 16961 | 0.0091976 | 0.1049893 | 108.72436 | 2.0363269 |
| GO:0070265 | necrotic cell death | 1 | 156 | 1 | 16961 | 0.0091976 | 0.1049893 | 108.72436 | 2.0363269 |
| GO:2000055 | positive regulation of Wnt signaling pathway involved in dorsal/ventral axis specification | 1 | 156 | 1 | 16961 | 0.0091976 | 0.1049893 | 108.72436 | 2.0363269 |
| GO:0072434 | signal transduction involved in mitotic G2 DNA damage checkpoint | 1 | 156 | 1 | 16961 | 0.0091976 | 0.1049893 | 108.72436 | 2.0363269 |
| GO:0097369 | sodium ion import | 1 | 156 | 1 | 16961 | 0.0091976 | 0.1049893 | 108.72436 | 2.0363269 |
| GO:1902044 | regulation of Fas signaling pathway | 1 | 156 | 1 | 16961 | 0.0091976 | 0.1049893 | 108.72436 | 2.0363269 |
| GO:0060284 | regulation of cell development | 1 | 156 | 1 | 16961 | 0.0091976 | 0.1049893 | 108.72436 | 2.0363269 |
| GO:0010616 | negative regulation of cardiac muscle adaptation | 1 | 156 | 1 | 16961 | 0.0091976 | 0.1049893 | 108.72436 | 2.0363269 |
| GO:0010869 | regulation of receptor biosynthetic process | 1 | 156 | 1 | 16961 | 0.0091976 | 0.1049893 | 108.72436 | 2.0363269 |
| GO:0060051 | negative regulation of protein glycosylation | 1 | 156 | 1 | 16961 | 0.0091976 | 0.1049893 | 108.72436 | 2.0363269 |
| GO:0044557 | relaxation of smooth muscle | 1 | 156 | 1 | 16961 | 0.0091976 | 0.1049893 | 108.72436 | 2.0363269 |
| GO:0034728 | nucleosome organization | 1 | 156 | 1 | 16961 | 0.0091976 | 0.1049893 | 108.72436 | 2.0363269 |
| GO:0031441 | negative regulation of mRNA 3'-end processing | 1 | 156 | 1 | 16961 | 0.0091976 | 0.1049893 | 108.72436 | 2.0363269 |
| GO:0051293 | establishment of spindle localization | 1 | 156 | 1 | 16961 | 0.0091976 | 0.1049893 | 108.72436 | 2.0363269 |
| GO:0097298 | regulation of nucleus size | 1 | 156 | 1 | 16961 | 0.0091976 | 0.1049893 | 108.72436 | 2.0363269 |
| GO:0070447 | positive regulation of oligodendrocyte progenitor proliferation | 1 | 156 | 1 | 16961 | 0.0091976 | 0.1049893 | 108.72436 | 2.0363269 |
| GO:0042182 | ketone catabolic process | 1 | 156 | 1 | 16961 | 0.0091976 | 0.1049893 | 108.72436 | 2.0363269 |
| GO:0002316 | follicular B cell differentiation | 1 | 156 | 1 | 16961 | 0.0091976 | 0.1049893 | 108.72436 | 2.0363269 |
| GO:0070646 | protein modification by small protein removal | 1 | 156 | 1 | 16961 | 0.0091976 | 0.1049893 | 108.72436 | 2.0363269 |
| GO:0090025 | regulation of monocyte chemotaxis | 1 | 156 | 1 | 16961 | 0.0091976 | 0.1049893 | 108.72436 | 2.0363269 |
| GO:0070667 | negative regulation of mast cell proliferation | 1 | 156 | 1 | 16961 | 0.0091976 | 0.1049893 | 108.72436 | 2.0363269 |
| GO:0006357 | regulation of transcription from RNA polymerase II promoter | 11 | 156 | 530 | 16961 | 0.0099597 | 0.1124918 | 2.2565433 | 2.0017554 |
| GO:0016925 | protein sumoylation | 4 | 156 | 92 | 16961 | 0.0102903 | 0.1131686 | 4.727146 | 1.9875707 |
| GO:0042325 | regulation of phosphorylation | 2 | 156 | 17 | 16961 | 0.0104415 | 0.1131686 | 12.791101 | 1.9812385 |
| GO:1902041 | regulation of extrinsic apoptotic signaling pathway via death domain receptors | 2 | 156 | 17 | 16961 | 0.0104415 | 0.1131686 | 12.791101 | 1.9812385 |
| GO:0030889 | negative regulation of B cell proliferation | 2 | 156 | 17 | 16961 | 0.0104415 | 0.1131686 | 12.791101 | 1.9812385 |
| GO:0045892 | negative regulation of transcription, DNA-templated | 11 | 156 | 535 | 16961 | 0.0106329 | 0.1140909 | 2.2354541 | 1.9733487 |
| GO:0071320 | cellular response to cAMP | 3 | 156 | 50 | 16961 | 0.010907 | 0.1158733 | 6.5234615 | 1.962295 |
| GO:0001756 | somitogenesis | 3 | 156 | 51 | 16961 | 0.0115118 | 0.1210999 | 6.3955505 | 1.9388557 |
| GO:0070301 | cellular response to hydrogen peroxide | 3 | 156 | 53 | 16961 | 0.0127786 | 0.1292618 | 6.154209 | 1.8935178 |
| GO:0038095 | Fc-epsilon receptor signaling pathway | 8 | 156 | 337 | 16961 | 0.0128364 | 0.1292618 | 2.5809937 | 1.8915574 |
| GO:0010881 | regulation of cardiac muscle contraction by regulation of the release of sequestered calcium ion | 2 | 156 | 19 | 16961 | 0.0129719 | 0.1292618 | 11.444669 | 1.8869962 |
| GO:0033198 | response to ATP | 2 | 156 | 20 | 16961 | 0.0143271 | 0.1292618 | 10.872436 | 1.8438432 |
| GO:0048384 | retinoic acid receptor signaling pathway | 2 | 156 | 20 | 16961 | 0.0143271 | 0.1292618 | 10.872436 | 1.8438432 |
| GO:0032259 | methylation | 6 | 156 | 215 | 16961 | 0.0146274 | 0.1292618 | 3.0341682 | 1.8348321 |
| GO:0035162 | embryonic hemopoiesis | 2 | 156 | 21 | 16961 | 0.0157406 | 0.1292618 | 10.354701 | 1.80298 |
| GO:0023014 | signal transduction by phosphorylation | 3 | 156 | 58 | 16961 | 0.016282 | 0.1292618 | 5.6236737 | 1.7882912 |
| GO:0070932 | histone H3 deacetylation | 2 | 156 | 22 | 16961 | 0.0172112 | 0.1292618 | 9.8840326 | 1.7641879 |
| GO:0006974 | cellular response to DNA damage stimulus | 9 | 156 | 427 | 16961 | 0.0173748 | 0.1292618 | 2.2916141 | 1.7600802 |
| GO:0007219 | Notch signaling pathway | 5 | 156 | 163 | 16961 | 0.0174437 | 0.1292618 | 3.335103 | 1.7583624 |
| GO:0015031 | protein transport | 12 | 156 | 656 | 16961 | 0.018158 | 0.1292618 | 1.9888602 | 1.7409331 |
| GO:0060369 | positive regulation of Fc receptor mediated stimulatory signaling pathway | 1 | 156 | 2 | 16961 | 0.0183111 | 0.1292618 | 54.362179 | 1.7372859 |
| GO:0050794 | regulation of cellular process | 1 | 156 | 2 | 16961 | 0.0183111 | 0.1292618 | 54.362179 | 1.7372859 |
| GO:1902532 | negative regulation of intracellular signal transduction | 1 | 156 | 2 | 16961 | 0.0183111 | 0.1292618 | 54.362179 | 1.7372859 |
| GO:0071233 | cellular response to leucine | 1 | 156 | 2 | 16961 | 0.0183111 | 0.1292618 | 54.362179 | 1.7372859 |
| GO:1902170 | cellular response to reactive nitrogen species | 1 | 156 | 2 | 16961 | 0.0183111 | 0.1292618 | 54.362179 | 1.7372859 |
| GO:0021874 | Wnt signaling pathway involved in forebrain neuroblast division | 1 | 156 | 2 | 16961 | 0.0183111 | 0.1292618 | 54.362179 | 1.7372859 |
| GO:0021872 | forebrain generation of neurons | 1 | 156 | 2 | 16961 | 0.0183111 | 0.1292618 | 54.362179 | 1.7372859 |
| GO:0021849 | neuroblast division in subventricular zone | 1 | 156 | 2 | 16961 | 0.0183111 | 0.1292618 | 54.362179 | 1.7372859 |
| GO:0006478 | peptidyl-tyrosine sulfation | 1 | 156 | 2 | 16961 | 0.0183111 | 0.1292618 | 54.362179 | 1.7372859 |
| GO:0042264 | peptidyl-aspartic acid hydroxylation | 1 | 156 | 2 | 16961 | 0.0183111 | 0.1292618 | 54.362179 | 1.7372859 |
| GO:0031585 | regulation of inositol 1,4,5-trisphosphate-sensitive calcium-release channel activity | 1 | 156 | 2 | 16961 | 0.0183111 | 0.1292618 | 54.362179 | 1.7372859 |
| GO:0043314 | negative regulation of neutrophil degranulation | 1 | 156 | 2 | 16961 | 0.0183111 | 0.1292618 | 54.362179 | 1.7372859 |
| GO:0035507 | regulation of myosin-light-chain-phosphatase activity | 1 | 156 | 2 | 16961 | 0.0183111 | 0.1292618 | 54.362179 | 1.7372859 |
| GO:2000629 | negative regulation of miRNA metabolic process | 1 | 156 | 2 | 16961 | 0.0183111 | 0.1292618 | 54.362179 | 1.7372859 |
| GO:0035305 | negative regulation of dephosphorylation | 1 | 156 | 2 | 16961 | 0.0183111 | 0.1292618 | 54.362179 | 1.7372859 |
| GO:0071436 | sodium ion export | 1 | 156 | 2 | 16961 | 0.0183111 | 0.1292618 | 54.362179 | 1.7372859 |
| GO:0090169 | regulation of spindle assembly | 1 | 156 | 2 | 16961 | 0.0183111 | 0.1292618 | 54.362179 | 1.7372859 |
| GO:0002692 | negative regulation of cellular extravasation | 1 | 156 | 2 | 16961 | 0.0183111 | 0.1292618 | 54.362179 | 1.7372859 |
| GO:0090118 | receptor-mediated endocytosis of low-density lipoprotein particle involved in cholesterol transport | 1 | 156 | 2 | 16961 | 0.0183111 | 0.1292618 | 54.362179 | 1.7372859 |
| GO:0060977 | coronary vasculature morphogenesis | 1 | 156 | 2 | 16961 | 0.0183111 | 0.1292618 | 54.362179 | 1.7372859 |
| GO:0002431 | Fc receptor mediated stimulatory signaling pathway | 1 | 156 | 2 | 16961 | 0.0183111 | 0.1292618 | 54.362179 | 1.7372859 |
| GO:0061324 | canonical Wnt signaling pathway involved in positive regulation of cardiac outflow tract cell proliferation | 1 | 156 | 2 | 16961 | 0.0183111 | 0.1292618 | 54.362179 | 1.7372859 |
| GO:0035261 | external genitalia morphogenesis | 1 | 156 | 2 | 16961 | 0.0183111 | 0.1292618 | 54.362179 | 1.7372859 |
| GO:0002337 | B-1a B cell differentiation | 1 | 156 | 2 | 16961 | 0.0183111 | 0.1292618 | 54.362179 | 1.7372859 |
| GO:0030862 | positive regulation of polarized epithelial cell differentiation | 1 | 156 | 2 | 16961 | 0.0183111 | 0.1292618 | 54.362179 | 1.7372859 |
| GO:0048332 | mesoderm morphogenesis | 1 | 156 | 2 | 16961 | 0.0183111 | 0.1292618 | 54.362179 | 1.7372859 |
| GO:0040030 | regulation of molecular function, epigenetic | 1 | 156 | 2 | 16961 | 0.0183111 | 0.1292618 | 54.362179 | 1.7372859 |
| GO:0021506 | anterior neuropore closure | 1 | 156 | 2 | 16961 | 0.0183111 | 0.1292618 | 54.362179 | 1.7372859 |
| GO:0044335 | canonical Wnt signaling pathway involved in neural crest cell differentiation | 1 | 156 | 2 | 16961 | 0.0183111 | 0.1292618 | 54.362179 | 1.7372859 |
| GO:0044340 | canonical Wnt signaling pathway involved in regulation of cell proliferation | 1 | 156 | 2 | 16961 | 0.0183111 | 0.1292618 | 54.362179 | 1.7372859 |
| GO:0071955 | recycling endosome to Golgi transport | 1 | 156 | 2 | 16961 | 0.0183111 | 0.1292618 | 54.362179 | 1.7372859 |
| GO:0070828 | heterochromatin organization | 1 | 156 | 2 | 16961 | 0.0183111 | 0.1292618 | 54.362179 | 1.7372859 |
| GO:0035617 | stress granule disassembly | 1 | 156 | 2 | 16961 | 0.0183111 | 0.1292618 | 54.362179 | 1.7372859 |
| GO:0021691 | cerebellar Purkinje cell layer maturation | 1 | 156 | 2 | 16961 | 0.0183111 | 0.1292618 | 54.362179 | 1.7372859 |
| GO:0035441 | cell migration involved in vasculogenesis | 1 | 156 | 2 | 16961 | 0.0183111 | 0.1292618 | 54.362179 | 1.7372859 |
| GO:0035408 | histone H3-T6 phosphorylation | 1 | 156 | 2 | 16961 | 0.0183111 | 0.1292618 | 54.362179 | 1.7372859 |
| GO:0035887 | aortic smooth muscle cell differentiation | 1 | 156 | 2 | 16961 | 0.0183111 | 0.1292618 | 54.362179 | 1.7372859 |
| GO:0090234 | regulation of kinetochore assembly | 1 | 156 | 2 | 16961 | 0.0183111 | 0.1292618 | 54.362179 | 1.7372859 |
| GO:0070372 | regulation of ERK1 and ERK2 cascade | 2 | 156 | 23 | 16961 | 0.0187379 | 0.1297149 | 9.4542921 | 1.727278 |
| GO:0051568 | histone H3-K4 methylation | 2 | 156 | 23 | 16961 | 0.0187379 | 0.1297149 | 9.4542921 | 1.727278 |
| GO:0097300 | programmed necrotic cell death | 2 | 156 | 23 | 16961 | 0.0187379 | 0.1297149 | 9.4542921 | 1.727278 |
| GO:0000122 | negative regulation of transcription from RNA polymerase II promoter | 13 | 156 | 739 | 16961 | 0.0189746 | 0.1305114 | 1.9126071 | 1.7218267 |
| GO:0060397 | JAK-STAT cascade involved in growth hormone signaling pathway | 2 | 156 | 24 | 16961 | 0.0203195 | 0.1388717 | 9.0603632 | 1.6920862 |
| GO:0042147 | retrograde transport, endosome to Golgi | 3 | 156 | 64 | 16961 | 0.0211289 | 0.143489 | 5.0964543 | 1.6751239 |
| GO:0016571 | histone methylation | 2 | 156 | 25 | 16961 | 0.0219549 | 0.1438099 | 8.6979487 | 1.6584687 |
| GO:0038096 | Fc-gamma receptor signaling pathway involved in phagocytosis | 4 | 156 | 116 | 16961 | 0.0222939 | 0.1438099 | 3.7491158 | 1.6518144 |
| GO:0001933 | negative regulation of protein phosphorylation | 3 | 156 | 66 | 16961 | 0.0229013 | 0.1438099 | 4.9420163 | 1.6401394 |
| GO:0006906 | vesicle fusion | 2 | 156 | 27 | 16961 | 0.0253824 | 0.1438099 | 8.0536562 | 1.5954665 |
| GO:0002223 | stimulatory C-type lectin receptor signaling pathway | 4 | 156 | 121 | 16961 | 0.0255459 | 0.1438099 | 3.5941937 | 1.592679 |
| GO:0035108 | limb morphogenesis | 2 | 156 | 28 | 16961 | 0.0271725 | 0.1438099 | 7.7660256 | 1.5658707 |
| GO:0046060 | dATP metabolic process | 1 | 156 | 3 | 16961 | 0.0273413 | 0.1438099 | 36.241453 | 1.5631808 |
| GO:1901660 | calcium ion export | 1 | 156 | 3 | 16961 | 0.0273413 | 0.1438099 | 36.241453 | 1.5631808 |
| GO:0060313 | negative regulation of blood vessel remodeling | 1 | 156 | 3 | 16961 | 0.0273413 | 0.1438099 | 36.241453 | 1.5631808 |
| GO:0032959 | inositol trisphosphate biosynthetic process | 1 | 156 | 3 | 16961 | 0.0273413 | 0.1438099 | 36.241453 | 1.5631808 |
| GO:0031664 | regulation of lipopolysaccharide-mediated signaling pathway | 1 | 156 | 3 | 16961 | 0.0273413 | 0.1438099 | 36.241453 | 1.5631808 |
| GO:0060177 | regulation of angiotensin metabolic process | 1 | 156 | 3 | 16961 | 0.0273413 | 0.1438099 | 36.241453 | 1.5631808 |
| GO:0097676 | histone H3-K36 dimethylation | 1 | 156 | 3 | 16961 | 0.0273413 | 0.1438099 | 36.241453 | 1.5631808 |
| GO:0033512 | L-lysine catabolic process to acetyl-CoA via saccharopine | 1 | 156 | 3 | 16961 | 0.0273413 | 0.1438099 | 36.241453 | 1.5631808 |
| GO:0018193 | peptidyl-amino acid modification | 1 | 156 | 3 | 16961 | 0.0273413 | 0.1438099 | 36.241453 | 1.5631808 |
| GO:0035973 | aggrephagy | 1 | 156 | 3 | 16961 | 0.0273413 | 0.1438099 | 36.241453 | 1.5631808 |
| GO:0060544 | regulation of necroptotic process | 1 | 156 | 3 | 16961 | 0.0273413 | 0.1438099 | 36.241453 | 1.5631808 |
| GO:0019065 | receptor-mediated endocytosis of virus by host cell | 1 | 156 | 3 | 16961 | 0.0273413 | 0.1438099 | 36.241453 | 1.5631808 |
| GO:2000670 | positive regulation of dendritic cell apoptotic process | 1 | 156 | 3 | 16961 | 0.0273413 | 0.1438099 | 36.241453 | 1.5631808 |
| GO:0035523 | protein K29-linked deubiquitination | 1 | 156 | 3 | 16961 | 0.0273413 | 0.1438099 | 36.241453 | 1.5631808 |
| GO:2000681 | negative regulation of rubidium ion transport | 1 | 156 | 3 | 16961 | 0.0273413 | 0.1438099 | 36.241453 | 1.5631808 |
| GO:2000687 | negative regulation of rubidium ion transmembrane transporter activity | 1 | 156 | 3 | 16961 | 0.0273413 | 0.1438099 | 36.241453 | 1.5631808 |
| GO:0000189 | MAPK import into nucleus | 1 | 156 | 3 | 16961 | 0.0273413 | 0.1438099 | 36.241453 | 1.5631808 |
| GO:2000051 | negative regulation of non-canonical Wnt signaling pathway | 1 | 156 | 3 | 16961 | 0.0273413 | 0.1438099 | 36.241453 | 1.5631808 |
| GO:0071596 | ubiquitin-dependent protein catabolic process via the N-end rule pathway | 1 | 156 | 3 | 16961 | 0.0273413 | 0.1438099 | 36.241453 | 1.5631808 |
| GO:0006991 | response to sterol depletion | 1 | 156 | 3 | 16961 | 0.0273413 | 0.1438099 | 36.241453 | 1.5631808 |
| GO:0010829 | negative regulation of glucose transport | 1 | 156 | 3 | 16961 | 0.0273413 | 0.1438099 | 36.241453 | 1.5631808 |
| GO:0019858 | cytosine metabolic process | 1 | 156 | 3 | 16961 | 0.0273413 | 0.1438099 | 36.241453 | 1.5631808 |
| GO:0051153 | regulation of striated muscle cell differentiation | 1 | 156 | 3 | 16961 | 0.0273413 | 0.1438099 | 36.241453 | 1.5631808 |
| GO:1990168 | protein K33-linked deubiquitination | 1 | 156 | 3 | 16961 | 0.0273413 | 0.1438099 | 36.241453 | 1.5631808 |
| GO:0070602 | regulation of centromeric sister chromatid cohesion | 1 | 156 | 3 | 16961 | 0.0273413 | 0.1438099 | 36.241453 | 1.5631808 |
| GO:0043243 | positive regulation of protein complex disassembly | 1 | 156 | 3 | 16961 | 0.0273413 | 0.1438099 | 36.241453 | 1.5631808 |
| GO:0002902 | regulation of B cell apoptotic process | 1 | 156 | 3 | 16961 | 0.0273413 | 0.1438099 | 36.241453 | 1.5631808 |
| GO:0018076 | N-terminal peptidyl-lysine acetylation | 1 | 156 | 3 | 16961 | 0.0273413 | 0.1438099 | 36.241453 | 1.5631808 |
| GO:1901740 | negative regulation of myoblast fusion | 1 | 156 | 3 | 16961 | 0.0273413 | 0.1438099 | 36.241453 | 1.5631808 |
| GO:0060401 | cytosolic calcium ion transport | 1 | 156 | 3 | 16961 | 0.0273413 | 0.1438099 | 36.241453 | 1.5631808 |
| GO:0002762 | negative regulation of myeloid leukocyte differentiation | 1 | 156 | 3 | 16961 | 0.0273413 | 0.1438099 | 36.241453 | 1.5631808 |
| GO:0002513 | tolerance induction to self antigen | 1 | 156 | 3 | 16961 | 0.0273413 | 0.1438099 | 36.241453 | 1.5631808 |
| GO:0014737 | positive regulation of muscle atrophy | 1 | 156 | 3 | 16961 | 0.0273413 | 0.1438099 | 36.241453 | 1.5631808 |
| GO:0033003 | regulation of mast cell activation | 1 | 156 | 3 | 16961 | 0.0273413 | 0.1438099 | 36.241453 | 1.5631808 |
| GO:0031060 | regulation of histone methylation | 1 | 156 | 3 | 16961 | 0.0273413 | 0.1438099 | 36.241453 | 1.5631808 |
| GO:0003401 | axis elongation | 1 | 156 | 3 | 16961 | 0.0273413 | 0.1438099 | 36.241453 | 1.5631808 |
| GO:0034140 | negative regulation of toll-like receptor 3 signaling pathway | 1 | 156 | 3 | 16961 | 0.0273413 | 0.1438099 | 36.241453 | 1.5631808 |
| GO:0002331 | pre-B cell allelic exclusion | 1 | 156 | 3 | 16961 | 0.0273413 | 0.1438099 | 36.241453 | 1.5631808 |
| GO:0002774 | Fc receptor mediated inhibitory signaling pathway | 1 | 156 | 3 | 16961 | 0.0273413 | 0.1438099 | 36.241453 | 1.5631808 |
| GO:0090245 | axis elongation involved in somitogenesis | 1 | 156 | 3 | 16961 | 0.0273413 | 0.1438099 | 36.241453 | 1.5631808 |
| GO:0010468 | regulation of gene expression | 5 | 156 | 187 | 16961 | 0.0294002 | 0.1526983 | 2.9070684 | 1.5316503 |
| GO:0071300 | cellular response to retinoic acid | 3 | 156 | 73 | 16961 | 0.0297227 | 0.1526983 | 4.4681243 | 1.5269124 |
| GO:0001666 | response to hypoxia | 5 | 156 | 190 | 16961 | 0.0311841 | 0.1526983 | 2.8611673 | 1.5060664 |
| GO:0006417 | regulation of translation | 4 | 156 | 129 | 16961 | 0.0313154 | 0.1526983 | 3.371298 | 1.5042425 |
| GO:0006897 | endocytosis | 5 | 156 | 191 | 16961 | 0.0317936 | 0.1526983 | 2.8461874 | 1.4976606 |
| GO:0097190 | apoptotic signaling pathway | 4 | 156 | 130 | 16961 | 0.0320861 | 0.1526983 | 3.3453649 | 1.4936829 |
| GO:0050766 | positive regulation of phagocytosis | 2 | 156 | 31 | 16961 | 0.0328349 | 0.1526983 | 7.0144748 | 1.4836643 |
| GO:0031663 | lipopolysaccharide-mediated signaling pathway | 2 | 156 | 31 | 16961 | 0.0328349 | 0.1526983 | 7.0144748 | 1.4836643 |
| GO:0048568 | embryonic organ development | 2 | 156 | 31 | 16961 | 0.0328349 | 0.1526983 | 7.0144748 | 1.4836643 |
| GO:0071902 | positive regulation of protein serine/threonine kinase activity | 2 | 156 | 31 | 16961 | 0.0328349 | 0.1526983 | 7.0144748 | 1.4836643 |
| GO:0007018 | microtubule-based movement | 3 | 156 | 76 | 16961 | 0.0329388 | 0.1526983 | 4.291751 | 1.4822917 |
| GO:0043065 | positive regulation of apoptotic process | 7 | 156 | 330 | 16961 | 0.0331055 | 0.1526983 | 2.3062743 | 1.4800997 |
| GO:0009887 | organ morphogenesis | 4 | 156 | 132 | 16961 | 0.0336609 | 0.1526983 | 3.2946775 | 1.4728747 |
| GO:0051092 | positive regulation of NF-kappaB transcription factor activity | 4 | 156 | 135 | 16961 | 0.0361064 | 0.1526983 | 3.2214625 | 1.4424162 |
| GO:0071494 | cellular response to UV-C | 1 | 156 | 4 | 16961 | 0.036289 | 0.1526983 | 27.18109 | 1.4402252 |
| GO:0070164 | negative regulation of adiponectin secretion | 1 | 156 | 4 | 16961 | 0.036289 | 0.1526983 | 27.18109 | 1.4402252 |
| GO:0060596 | mammary placode formation | 1 | 156 | 4 | 16961 | 0.036289 | 0.1526983 | 27.18109 | 1.4402252 |
| GO:0000056 | ribosomal small subunit export from nucleus | 1 | 156 | 4 | 16961 | 0.036289 | 0.1526983 | 27.18109 | 1.4402252 |
| GO:0016241 | regulation of macroautophagy | 1 | 156 | 4 | 16961 | 0.036289 | 0.1526983 | 27.18109 | 1.4402252 |
| GO:1902175 | regulation of oxidative stress-induced intrinsic apoptotic signaling pathway | 1 | 156 | 4 | 16961 | 0.036289 | 0.1526983 | 27.18109 | 1.4402252 |
| GO:1901097 | negative regulation of autophagic vacuole maturation | 1 | 156 | 4 | 16961 | 0.036289 | 0.1526983 | 27.18109 | 1.4402252 |
| GO:0038066 | p38MAPK cascade | 1 | 156 | 4 | 16961 | 0.036289 | 0.1526983 | 27.18109 | 1.4402252 |
| GO:0042998 | positive regulation of Golgi to plasma membrane protein transport | 1 | 156 | 4 | 16961 | 0.036289 | 0.1526983 | 27.18109 | 1.4402252 |
| GO:0010715 | regulation of extracellular matrix disassembly | 1 | 156 | 4 | 16961 | 0.036289 | 0.1526983 | 27.18109 | 1.4402252 |
| GO:0010961 | cellular magnesium ion homeostasis | 1 | 156 | 4 | 16961 | 0.036289 | 0.1526983 | 27.18109 | 1.4402252 |
| GO:0090330 | regulation of platelet aggregation | 1 | 156 | 4 | 16961 | 0.036289 | 0.1526983 | 27.18109 | 1.4402252 |
| GO:0007028 | cytoplasm organization | 1 | 156 | 4 | 16961 | 0.036289 | 0.1526983 | 27.18109 | 1.4402252 |
| GO:0033522 | histone H2A ubiquitination | 1 | 156 | 4 | 16961 | 0.036289 | 0.1526983 | 27.18109 | 1.4402252 |
| GO:0035984 | cellular response to trichostatin A | 1 | 156 | 4 | 16961 | 0.036289 | 0.1526983 | 27.18109 | 1.4402252 |
| GO:0070940 | dephosphorylation of RNA polymerase II C-terminal domain | 1 | 156 | 4 | 16961 | 0.036289 | 0.1526983 | 27.18109 | 1.4402252 |
| GO:0090170 | regulation of Golgi inheritance | 1 | 156 | 4 | 16961 | 0.036289 | 0.1526983 | 27.18109 | 1.4402252 |
| GO:0060535 | trachea cartilage morphogenesis | 1 | 156 | 4 | 16961 | 0.036289 | 0.1526983 | 27.18109 | 1.4402252 |
| GO:0090116 | C-5 methylation of cytosine | 1 | 156 | 4 | 16961 | 0.036289 | 0.1526983 | 27.18109 | 1.4402252 |
| GO:0072718 | response to cisplatin | 1 | 156 | 4 | 16961 | 0.036289 | 0.1526983 | 27.18109 | 1.4402252 |
| GO:2000021 | regulation of ion homeostasis | 1 | 156 | 4 | 16961 | 0.036289 | 0.1526983 | 27.18109 | 1.4402252 |
| GO:0071389 | cellular response to mineralocorticoid stimulus | 1 | 156 | 4 | 16961 | 0.036289 | 0.1526983 | 27.18109 | 1.4402252 |
| GO:0071322 | cellular response to carbohydrate stimulus | 1 | 156 | 4 | 16961 | 0.036289 | 0.1526983 | 27.18109 | 1.4402252 |
| GO:0031936 | negative regulation of chromatin silencing | 1 | 156 | 4 | 16961 | 0.036289 | 0.1526983 | 27.18109 | 1.4402252 |
| GO:0006574 | valine catabolic process | 1 | 156 | 4 | 16961 | 0.036289 | 0.1526983 | 27.18109 | 1.4402252 |
| GO:0021943 | formation of radial glial scaffolds | 1 | 156 | 4 | 16961 | 0.036289 | 0.1526983 | 27.18109 | 1.4402252 |
| GO:0038127 | ERBB signaling pathway | 1 | 156 | 4 | 16961 | 0.036289 | 0.1526983 | 27.18109 | 1.4402252 |
| GO:0042159 | lipoprotein catabolic process | 1 | 156 | 4 | 16961 | 0.036289 | 0.1526983 | 27.18109 | 1.4402252 |
| GO:0071947 | protein deubiquitination involved in ubiquitin-dependent protein catabolic process | 1 | 156 | 4 | 16961 | 0.036289 | 0.1526983 | 27.18109 | 1.4402252 |
| GO:0010587 | miRNA catabolic process | 1 | 156 | 4 | 16961 | 0.036289 | 0.1526983 | 27.18109 | 1.4402252 |
| GO:0046952 | ketone body catabolic process | 1 | 156 | 4 | 16961 | 0.036289 | 0.1526983 | 27.18109 | 1.4402252 |
| GO:0070816 | phosphorylation of RNA polymerase II C-terminal domain | 1 | 156 | 4 | 16961 | 0.036289 | 0.1526983 | 27.18109 | 1.4402252 |
| GO:0034340 | response to type I interferon | 1 | 156 | 4 | 16961 | 0.036289 | 0.1526983 | 27.18109 | 1.4402252 |
| GO:0050855 | regulation of B cell receptor signaling pathway | 1 | 156 | 4 | 16961 | 0.036289 | 0.1526983 | 27.18109 | 1.4402252 |
| GO:0048713 | regulation of oligodendrocyte differentiation | 1 | 156 | 4 | 16961 | 0.036289 | 0.1526983 | 27.18109 | 1.4402252 |
| GO:0034136 | negative regulation of toll-like receptor 2 signaling pathway | 1 | 156 | 4 | 16961 | 0.036289 | 0.1526983 | 27.18109 | 1.4402252 |
| GO:0070668 | positive regulation of mast cell proliferation | 1 | 156 | 4 | 16961 | 0.036289 | 0.1526983 | 27.18109 | 1.4402252 |
| GO:0007264 | small GTPase mediated signal transduction | 12 | 156 | 732 | 16961 | 0.0378871 | 0.1588 | 1.7823665 | 1.4215091 |
| GO:0042113 | B cell activation | 2 | 156 | 34 | 16961 | 0.0389142 | 0.1624708 | 6.3955505 | 1.4098914 |
| GO:0006469 | negative regulation of protein kinase activity | 3 | 156 | 82 | 16961 | 0.0398918 | 0.1625878 | 3.9777205 | 1.3991165 |
| GO:0030900 | forebrain development | 3 | 156 | 82 | 16961 | 0.0398918 | 0.1625878 | 3.9777205 | 1.3991165 |
| GO:0045944 | positive regulation of transcription from RNA polymerase II promoter | 15 | 156 | 993 | 16961 | 0.0401888 | 0.1625878 | 1.6423619 | 1.3958948 |
| GO:0008152 | metabolic process | 26 | 156 | 1996 | 16961 | 0.0421321 | 0.1625878 | 1.4162492 | 1.3753871 |
| GO:0010629 | negative regulation of gene expression | 4 | 156 | 142 | 16961 | 0.0422029 | 0.1625878 | 3.062658 | 1.3746581 |
| GO:0018108 | peptidyl-tyrosine phosphorylation | 4 | 156 | 143 | 16961 | 0.0431185 | 0.1625878 | 3.0412408 | 1.3653367 |
| GO:0060325 | face morphogenesis | 2 | 156 | 36 | 16961 | 0.0431855 | 0.1625878 | 6.0402422 | 1.3646617 |
| GO:0002053 | positive regulation of mesenchymal cell proliferation | 2 | 156 | 36 | 16961 | 0.0431855 | 0.1625878 | 6.0402422 | 1.3646617 |
| GO:0070555 | response to interleukin-1 | 2 | 156 | 36 | 16961 | 0.0431855 | 0.1625878 | 6.0402422 | 1.3646617 |
| GO:0042127 | regulation of cell proliferation | 5 | 156 | 209 | 16961 | 0.0440518 | 0.1625878 | 2.6010612 | 1.3560365 |
| GO:0007050 | cell cycle arrest | 4 | 156 | 145 | 16961 | 0.0449832 | 0.1625878 | 2.9992927 | 1.34695 |
| GO:0072584 | caveolin-mediated endocytosis | 1 | 156 | 5 | 16961 | 0.0451549 | 0.1625878 | 21.744872 | 1.3452954 |
| GO:0098735 | positive regulation of the force of heart contraction | 1 | 156 | 5 | 16961 | 0.0451549 | 0.1625878 | 21.744872 | 1.3452954 |
| GO:0003344 | pericardium morphogenesis | 1 | 156 | 5 | 16961 | 0.0451549 | 0.1625878 | 21.744872 | 1.3452954 |
| GO:0030917 | midbrain-hindbrain boundary development | 1 | 156 | 5 | 16961 | 0.0451549 | 0.1625878 | 21.744872 | 1.3452954 |
| GO:0070389 | chaperone cofactor-dependent protein refolding | 1 | 156 | 5 | 16961 | 0.0451549 | 0.1625878 | 21.744872 | 1.3452954 |
| GO:0030718 | germ-line stem cell maintenance | 1 | 156 | 5 | 16961 | 0.0451549 | 0.1625878 | 21.744872 | 1.3452954 |
| GO:0019932 | second-messenger-mediated signaling | 1 | 156 | 5 | 16961 | 0.0451549 | 0.1625878 | 21.744872 | 1.3452954 |
| GO:0042531 | positive regulation of tyrosine phosphorylation of STAT protein | 1 | 156 | 5 | 16961 | 0.0451549 | 0.1625878 | 21.744872 | 1.3452954 |
| GO:0008626 | granzyme-mediated apoptotic signaling pathway | 1 | 156 | 5 | 16961 | 0.0451549 | 0.1625878 | 21.744872 | 1.3452954 |
| GO:0021861 | forebrain radial glial cell differentiation | 1 | 156 | 5 | 16961 | 0.0451549 | 0.1625878 | 21.744872 | 1.3452954 |
| GO:0030578 | PML body organization | 1 | 156 | 5 | 16961 | 0.0451549 | 0.1625878 | 21.744872 | 1.3452954 |
| GO:0035948 | positive regulation of gluconeogenesis by positive regulation of transcription from RNA polymerase II promoter | 1 | 156 | 5 | 16961 | 0.0451549 | 0.1625878 | 21.744872 | 1.3452954 |
| GO:0070734 | histone H3-K27 methylation | 1 | 156 | 5 | 16961 | 0.0451549 | 0.1625878 | 21.744872 | 1.3452954 |
| GO:0043550 | regulation of lipid kinase activity | 1 | 156 | 5 | 16961 | 0.0451549 | 0.1625878 | 21.744872 | 1.3452954 |
| GO:0060903 | positive regulation of meiosis I | 1 | 156 | 5 | 16961 | 0.0451549 | 0.1625878 | 21.744872 | 1.3452954 |
| GO:0033088 | negative regulation of immature T cell proliferation in thymus | 1 | 156 | 5 | 16961 | 0.0451549 | 0.1625878 | 21.744872 | 1.3452954 |
| GO:0061157 | mRNA destabilization | 1 | 156 | 5 | 16961 | 0.0451549 | 0.1625878 | 21.744872 | 1.3452954 |
| GO:0045646 | regulation of erythrocyte differentiation | 1 | 156 | 5 | 16961 | 0.0451549 | 0.1625878 | 21.744872 | 1.3452954 |
| GO:0018023 | peptidyl-lysine trimethylation | 1 | 156 | 5 | 16961 | 0.0451549 | 0.1625878 | 21.744872 | 1.3452954 |
| GO:0018027 | peptidyl-lysine dimethylation | 1 | 156 | 5 | 16961 | 0.0451549 | 0.1625878 | 21.744872 | 1.3452954 |
| GO:0000715 | nucleotide-excision repair, DNA damage recognition | 1 | 156 | 5 | 16961 | 0.0451549 | 0.1625878 | 21.744872 | 1.3452954 |
| GO:0051054 | positive regulation of DNA metabolic process | 1 | 156 | 5 | 16961 | 0.0451549 | 0.1625878 | 21.744872 | 1.3452954 |
| GO:0010560 | positive regulation of glycoprotein biosynthetic process | 1 | 156 | 5 | 16961 | 0.0451549 | 0.1625878 | 21.744872 | 1.3452954 |
| GO:0046950 | cellular ketone body metabolic process | 1 | 156 | 5 | 16961 | 0.0451549 | 0.1625878 | 21.744872 | 1.3452954 |
| GO:0001842 | neural fold formation | 1 | 156 | 5 | 16961 | 0.0451549 | 0.1625878 | 21.744872 | 1.3452954 |
| GO:0002768 | immune response-regulating cell surface receptor signaling pathway | 1 | 156 | 5 | 16961 | 0.0451549 | 0.1625878 | 21.744872 | 1.3452954 |
| GO:0035404 | histone-serine phosphorylation | 1 | 156 | 5 | 16961 | 0.0451549 | 0.1625878 | 21.744872 | 1.3452954 |
| GO:0034392 | negative regulation of smooth muscle cell apoptotic process | 1 | 156 | 5 | 16961 | 0.0451549 | 0.1625878 | 21.744872 | 1.3452954 |
| GO:0048742 | regulation of skeletal muscle fiber development | 1 | 156 | 5 | 16961 | 0.0451549 | 0.1625878 | 21.744872 | 1.3452954 |
| GO:0002553 | histamine secretion by mast cell | 1 | 156 | 5 | 16961 | 0.0451549 | 0.1625878 | 21.744872 | 1.3452954 |
| GO:0042059 | negative regulation of epidermal growth factor receptor signaling pathway | 2 | 156 | 37 | 16961 | 0.0453838 | 0.1628656 | 5.8769924 | 1.3430991 |
| GO:0048010 | vascular endothelial growth factor receptor signaling pathway | 6 | 156 | 281 | 16961 | 0.0456483 | 0.1632688 | 2.3215166 | 1.3405754 |
| GO:0007205 | protein kinase C-activating G-protein coupled receptor signaling pathway | 2 | 156 | 38 | 16961 | 0.0476226 | 0.1687385 | 5.7223347 | 1.3221871 |
