# Supplementary material for: Expression Profiles and Functional Analysis of Plasma Exosomal Circular RNAs in Acute Myocardial Infarction
Source: Biomed Res Int. 2022 Oct 1;2022:3458227. doi: 10.1155/2022/3458227 (PMC9547997; doi:10.1155/2022/3458227)
Supplement: Supplementary 7 — Supplementary Table S7: Biological process enrichment analyses of the differentially expressed exosomal circRNAs in comparison of AMI and CAD. [file 3458227.f7.docx]

Supplementary Table S7. Biological process enrichment analyses of the differentially expressed exosomal circRNAs in comparison of AMI and CAD.

| GOID | GOTerm | DifGene | AllDifGene | GeneInGO | AllGene | P-Value | FDR | Enrichment | (-log10P) |
| --- | --- | --- | --- | --- | --- | --- | --- | --- | --- |
| GO:0034968 | histone lysine methylation | 5 | 190 | 29 | 16961 | 1.597E-05 | 0.0134246 | 15.391107 | 4.7965859 |
| GO:0031098 | stress-activated protein kinase signaling cascade | 5 | 190 | 33 | 16961 | 3.079E-05 | 0.0134246 | 13.525518 | 4.5116523 |
| GO:0035556 | intracellular signal transduction | 17 | 190 | 488 | 16961 | 3.628E-05 | 0.0134246 | 3.1097606 | 4.4403011 |
| GO:0016568 | chromatin modification | 12 | 190 | 286 | 16961 | 9.473E-05 | 0.0230946 | 3.7455282 | 4.0235327 |
| GO:0006516 | glycoprotein catabolic process | 3 | 190 | 9 | 16961 | 0.0001106 | 0.0230946 | 29.75614 | 3.9562377 |
| GO:2000791 | negative regulation of mesenchymal cell proliferation involved in lung development | 2 | 190 | 2 | 16961 | 0.0001248 | 0.0230946 | 89.268421 | 3.9036619 |
| GO:0023014 | signal transduction by phosphorylation | 5 | 190 | 58 | 16961 | 0.000474 | 0.0751614 | 7.6955535 | 3.3242299 |
| GO:0008340 | determination of adult lifespan | 3 | 190 | 15 | 16961 | 0.0005701 | 0.079101 | 17.853684 | 3.2440512 |
| GO:0032147 | activation of protein kinase activity | 5 | 190 | 62 | 16961 | 0.0006457 | 0.0796317 | 7.1990662 | 3.1899944 |
| GO:0006325 | chromatin organization | 10 | 190 | 261 | 16961 | 0.0007382 | 0.079718 | 3.420246 | 3.1318162 |
| GO:0006468 | protein phosphorylation | 17 | 190 | 638 | 16961 | 0.0008541 | 0.079718 | 2.3786256 | 3.0684697 |
| GO:0006351 | transcription, DNA-templated | 43 | 190 | 2398 | 16961 | 0.0010144 | 0.079718 | 1.6007265 | 2.9938024 |
| GO:0006355 | regulation of transcription, DNA-templated | 45 | 190 | 2550 | 16961 | 0.0010521 | 0.079718 | 1.5753251 | 2.9779233 |
| GO:0031334 | positive regulation of protein complex assembly | 3 | 190 | 19 | 16961 | 0.0011747 | 0.079718 | 14.095014 | 2.9300648 |
| GO:0032237 | activation of store-operated calcium channel activity | 2 | 190 | 5 | 16961 | 0.0012209 | 0.079718 | 35.707368 | 2.9133178 |
| GO:0060964 | regulation of gene silencing by miRNA | 2 | 190 | 5 | 16961 | 0.0012209 | 0.079718 | 35.707368 | 2.9133178 |
| GO:0034773 | histone H4-K20 trimethylation | 2 | 190 | 5 | 16961 | 0.0012209 | 0.079718 | 35.707368 | 2.9133178 |
| GO:0007346 | regulation of mitotic cell cycle | 5 | 190 | 76 | 16961 | 0.0016251 | 0.1002175 | 5.8729224 | 2.7891069 |
| GO:0016926 | protein desumoylation | 2 | 190 | 6 | 16961 | 0.0018179 | 0.1048177 | 29.75614 | 2.7404395 |
| GO:0038096 | Fc-gamma receptor signaling pathway involved in phagocytosis | 6 | 190 | 116 | 16961 | 0.0019559 | 0.1048177 | 4.6173321 | 2.7086511 |
| GO:0051568 | histone H3-K4 methylation | 3 | 190 | 23 | 16961 | 0.0020775 | 0.1048177 | 11.643707 | 2.6824655 |
| GO:0045862 | positive regulation of proteolysis | 3 | 190 | 23 | 16961 | 0.0020775 | 0.1048177 | 11.643707 | 2.6824655 |
| GO:0060510 | Type II pneumocyte differentiation | 2 | 190 | 7 | 16961 | 0.0025263 | 0.1052137 | 25.505263 | 2.5975215 |
| GO:1903140 | regulation of establishment of endothelial barrier | 2 | 190 | 7 | 16961 | 0.0025263 | 0.1052137 | 25.505263 | 2.5975215 |
| GO:0031047 | gene silencing by RNA | 4 | 190 | 51 | 16961 | 0.0025281 | 0.1052137 | 7.0014448 | 2.5971993 |
| GO:0000289 | nuclear-transcribed mRNA poly(A) tail shortening | 3 | 190 | 25 | 16961 | 0.002654 | 0.1052137 | 10.712211 | 2.5760925 |
| GO:0051493 | regulation of cytoskeleton organization | 3 | 190 | 25 | 16961 | 0.002654 | 0.1052137 | 10.712211 | 2.5760925 |
| GO:0016571 | histone methylation | 3 | 190 | 25 | 16961 | 0.002654 | 0.1052137 | 10.712211 | 2.5760925 |
| GO:0032259 | methylation | 8 | 190 | 215 | 16961 | 0.0029682 | 0.1091577 | 3.3216157 | 2.5275114 |
| GO:0006306 | DNA methylation | 3 | 190 | 26 | 16961 | 0.0029757 | 0.1091577 | 10.300202 | 2.5264122 |
| GO:0016310 | phosphorylation | 18 | 190 | 785 | 16961 | 0.003184 | 0.1091577 | 2.0469192 | 2.4970251 |
| GO:0006417 | regulation of translation | 6 | 190 | 129 | 16961 | 0.0033285 | 0.1091577 | 4.1520196 | 2.4777548 |
| GO:0016441 | posttranscriptional gene silencing | 2 | 190 | 8 | 16961 | 0.0033436 | 0.1091577 | 22.317105 | 2.4757899 |
| GO:0001881 | receptor recycling | 2 | 190 | 8 | 16961 | 0.0033436 | 0.1091577 | 22.317105 | 2.4757899 |
| GO:0019722 | calcium-mediated signaling | 4 | 190 | 57 | 16961 | 0.0037937 | 0.1203145 | 6.2644506 | 2.4209369 |
| GO:0051533 | positive regulation of NFAT protein import into nucleus | 2 | 190 | 10 | 16961 | 0.0052949 | 0.1445861 | 17.853684 | 2.276141 |
| GO:0097202 | activation of cysteine-type endopeptidase activity | 2 | 190 | 11 | 16961 | 0.0064241 | 0.1445861 | 16.230622 | 2.1921892 |
| GO:0060213 | positive regulation of nuclear-transcribed mRNA poly(A) tail shortening | 2 | 190 | 11 | 16961 | 0.0064241 | 0.1445861 | 16.230622 | 2.1921892 |
| GO:0018105 | peptidyl-serine phosphorylation | 5 | 190 | 105 | 16961 | 0.0065523 | 0.1445861 | 4.2508772 | 2.1836051 |
| GO:0043124 | negative regulation of I-kappaB kinase/NF-kappaB signaling | 3 | 190 | 36 | 16961 | 0.0075295 | 0.1445861 | 7.4390351 | 2.1232325 |
| GO:0032088 | negative regulation of NF-kappaB transcription factor activity | 4 | 190 | 70 | 16961 | 0.0078709 | 0.1445861 | 5.1010526 | 2.1039746 |
| GO:0045944 | positive regulation of transcription from RNA polymerase II promoter | 20 | 190 | 993 | 16961 | 0.0079913 | 0.1445861 | 1.7979541 | 2.0973821 |
| GO:0030217 | T cell differentiation | 3 | 190 | 37 | 16961 | 0.0081273 | 0.1445861 | 7.2379801 | 2.0900534 |
| GO:1900153 | positive regulation of nuclear-transcribed mRNA catabolic process, deadenylation-dependent decay | 2 | 190 | 13 | 16961 | 0.0089775 | 0.1445861 | 13.733603 | 2.0468455 |
| GO:0033151 | V(D)J recombination | 2 | 190 | 13 | 16961 | 0.0089775 | 0.1445861 | 13.733603 | 2.0468455 |
| GO:0045618 | positive regulation of keratinocyte differentiation | 2 | 190 | 13 | 16961 | 0.0089775 | 0.1445861 | 13.733603 | 2.0468455 |
| GO:0032855 | positive regulation of Rac GTPase activity | 3 | 190 | 39 | 16961 | 0.0094047 | 0.1445861 | 6.8668016 | 2.0266572 |
| GO:0071044 | histone mRNA catabolic process | 2 | 190 | 14 | 16961 | 0.0103971 | 0.1445861 | 12.752632 | 1.9830884 |
| GO:0043122 | regulation of I-kappaB kinase/NF-kappaB signaling | 2 | 190 | 14 | 16961 | 0.0103971 | 0.1445861 | 12.752632 | 1.9830884 |
| GO:2000118 | regulation of sodium-dependent phosphate transport | 1 | 190 | 1 | 16961 | 0.0112022 | 0.1445861 | 89.268421 | 1.9506979 |
| GO:0072362 | regulation of glycolytic process by negative regulation of transcription from RNA polymerase II promoter | 1 | 190 | 1 | 16961 | 0.0112022 | 0.1445861 | 89.268421 | 1.9506979 |
| GO:2001229 | negative regulation of response to gamma radiation | 1 | 190 | 1 | 16961 | 0.0112022 | 0.1445861 | 89.268421 | 1.9506979 |
| GO:0036049 | peptidyl-lysine desuccinylation | 1 | 190 | 1 | 16961 | 0.0112022 | 0.1445861 | 89.268421 | 1.9506979 |
| GO:0036046 | protein demalonylation | 1 | 190 | 1 | 16961 | 0.0112022 | 0.1445861 | 89.268421 | 1.9506979 |
| GO:0036047 | peptidyl-lysine demalonylation | 1 | 190 | 1 | 16961 | 0.0112022 | 0.1445861 | 89.268421 | 1.9506979 |
| GO:0036048 | protein desuccinylation | 1 | 190 | 1 | 16961 | 0.0112022 | 0.1445861 | 89.268421 | 1.9506979 |
| GO:0010933 | positive regulation of macrophage tolerance induction | 1 | 190 | 1 | 16961 | 0.0112022 | 0.1445861 | 89.268421 | 1.9506979 |
| GO:0006214 | thymidine catabolic process | 1 | 190 | 1 | 16961 | 0.0112022 | 0.1445861 | 89.268421 | 1.9506979 |
| GO:0031343 | positive regulation of cell killing | 1 | 190 | 1 | 16961 | 0.0112022 | 0.1445861 | 89.268421 | 1.9506979 |
| GO:0006145 | purine nucleobase catabolic process | 1 | 190 | 1 | 16961 | 0.0112022 | 0.1445861 | 89.268421 | 1.9506979 |
| GO:0035508 | positive regulation of myosin-light-chain-phosphatase activity | 1 | 190 | 1 | 16961 | 0.0112022 | 0.1445861 | 89.268421 | 1.9506979 |
| GO:1901879 | regulation of protein depolymerization | 1 | 190 | 1 | 16961 | 0.0112022 | 0.1445861 | 89.268421 | 1.9506979 |
| GO:0050983 | deoxyhypusine biosynthetic process from spermidine | 1 | 190 | 1 | 16961 | 0.0112022 | 0.1445861 | 89.268421 | 1.9506979 |
| GO:0070571 | negative regulation of neuron projection regeneration | 1 | 190 | 1 | 16961 | 0.0112022 | 0.1445861 | 89.268421 | 1.9506979 |
| GO:0044268 | multicellular organismal protein metabolic process | 1 | 190 | 1 | 16961 | 0.0112022 | 0.1445861 | 89.268421 | 1.9506979 |
| GO:0071622 | regulation of granulocyte chemotaxis | 1 | 190 | 1 | 16961 | 0.0112022 | 0.1445861 | 89.268421 | 1.9506979 |
| GO:1903533 | regulation of protein targeting | 1 | 190 | 1 | 16961 | 0.0112022 | 0.1445861 | 89.268421 | 1.9506979 |
| GO:0070265 | necrotic cell death | 1 | 190 | 1 | 16961 | 0.0112022 | 0.1445861 | 89.268421 | 1.9506979 |
| GO:0072431 | signal transduction involved in mitotic G1 DNA damage checkpoint | 1 | 190 | 1 | 16961 | 0.0112022 | 0.1445861 | 89.268421 | 1.9506979 |
| GO:0072434 | signal transduction involved in mitotic G2 DNA damage checkpoint | 1 | 190 | 1 | 16961 | 0.0112022 | 0.1445861 | 89.268421 | 1.9506979 |
| GO:0010616 | negative regulation of cardiac muscle adaptation | 1 | 190 | 1 | 16961 | 0.0112022 | 0.1445861 | 89.268421 | 1.9506979 |
| GO:1900062 | regulation of replicative cell aging | 1 | 190 | 1 | 16961 | 0.0112022 | 0.1445861 | 89.268421 | 1.9506979 |
| GO:0021990 | neural plate formation | 1 | 190 | 1 | 16961 | 0.0112022 | 0.1445861 | 89.268421 | 1.9506979 |
| GO:0051341 | regulation of oxidoreductase activity | 1 | 190 | 1 | 16961 | 0.0112022 | 0.1445861 | 89.268421 | 1.9506979 |
| GO:0060051 | negative regulation of protein glycosylation | 1 | 190 | 1 | 16961 | 0.0112022 | 0.1445861 | 89.268421 | 1.9506979 |
| GO:0061141 | lung ciliated cell differentiation | 1 | 190 | 1 | 16961 | 0.0112022 | 0.1445861 | 89.268421 | 1.9506979 |
| GO:0000719 | photoreactive repair | 1 | 190 | 1 | 16961 | 0.0112022 | 0.1445861 | 89.268421 | 1.9506979 |
| GO:0043244 | regulation of protein complex disassembly | 1 | 190 | 1 | 16961 | 0.0112022 | 0.1445861 | 89.268421 | 1.9506979 |
| GO:0031441 | negative regulation of mRNA 3'-end processing | 1 | 190 | 1 | 16961 | 0.0112022 | 0.1445861 | 89.268421 | 1.9506979 |
| GO:0035624 | receptor transactivation | 1 | 190 | 1 | 16961 | 0.0112022 | 0.1445861 | 89.268421 | 1.9506979 |
| GO:0045620 | negative regulation of lymphocyte differentiation | 1 | 190 | 1 | 16961 | 0.0112022 | 0.1445861 | 89.268421 | 1.9506979 |
| GO:0010566 | regulation of ketone biosynthetic process | 1 | 190 | 1 | 16961 | 0.0112022 | 0.1445861 | 89.268421 | 1.9506979 |
| GO:0046355 | mannan catabolic process | 1 | 190 | 1 | 16961 | 0.0112022 | 0.1445861 | 89.268421 | 1.9506979 |
| GO:0002316 | follicular B cell differentiation | 1 | 190 | 1 | 16961 | 0.0112022 | 0.1445861 | 89.268421 | 1.9506979 |
| GO:2000795 | negative regulation of epithelial cell proliferation involved in lung morphogenesis | 1 | 190 | 1 | 16961 | 0.0112022 | 0.1445861 | 89.268421 | 1.9506979 |
| GO:0070646 | protein modification by small protein removal | 1 | 190 | 1 | 16961 | 0.0112022 | 0.1445861 | 89.268421 | 1.9506979 |
| GO:0009790 | embryo development | 3 | 190 | 42 | 16961 | 0.0115287 | 0.1470898 | 6.3763158 | 1.9382212 |
| GO:0051893 | regulation of focal adhesion assembly | 2 | 190 | 15 | 16961 | 0.0119089 | 0.1485271 | 11.902456 | 1.9241273 |
| GO:0010524 | positive regulation of calcium ion transport into cytosol | 2 | 190 | 15 | 16961 | 0.0119089 | 0.1485271 | 11.902456 | 1.9241273 |
| GO:0007010 | cytoskeleton organization | 5 | 190 | 122 | 16961 | 0.0120923 | 0.1491388 | 3.6585418 | 1.9174899 |
| GO:0006397 | mRNA processing | 9 | 190 | 335 | 16961 | 0.0134013 | 0.1634664 | 2.3982561 | 1.8728532 |
| GO:0048010 | vascular endothelial growth factor receptor signaling pathway | 8 | 190 | 281 | 16961 | 0.0140541 | 0.1693853 | 2.5414497 | 1.8521959 |
| GO:0006470 | protein dephosphorylation | 6 | 190 | 176 | 16961 | 0.0144462 | 0.1693853 | 3.0432416 | 1.8402454 |
| GO:0006606 | protein import into nucleus | 3 | 190 | 46 | 16961 | 0.0147569 | 0.1693853 | 5.8218535 | 1.8310063 |
| GO:0006893 | Golgi to plasma membrane transport | 2 | 190 | 17 | 16961 | 0.0152005 | 0.1693853 | 10.502167 | 1.8181424 |
| GO:0042445 | hormone metabolic process | 2 | 190 | 17 | 16961 | 0.0152005 | 0.1693853 | 10.502167 | 1.8181424 |
| GO:0035176 | social behavior | 3 | 190 | 47 | 16961 | 0.0156356 | 0.1693853 | 5.6979843 | 1.8058848 |
| GO:0006895 | Golgi to endosome transport | 2 | 190 | 18 | 16961 | 0.0169759 | 0.1693853 | 9.9187135 | 1.7701679 |
| GO:0042752 | regulation of circadian rhythm | 3 | 190 | 49 | 16961 | 0.01748 | 0.1693853 | 5.4654135 | 1.7574592 |
| GO:0008360 | regulation of cell shape | 5 | 190 | 134 | 16961 | 0.0175124 | 0.1693853 | 3.3309112 | 1.7566534 |
| GO:0043409 | negative regulation of MAPK cascade | 2 | 190 | 19 | 16961 | 0.0188348 | 0.1693853 | 9.3966759 | 1.7250383 |
| GO:0006376 | mRNA splice site selection | 2 | 190 | 19 | 16961 | 0.0188348 | 0.1693853 | 9.3966759 | 1.7250383 |
| GO:0048013 | ephrin receptor signaling pathway | 4 | 190 | 91 | 16961 | 0.019161 | 0.1693853 | 3.9238866 | 1.7175825 |
| GO:0043066 | negative regulation of apoptotic process | 12 | 190 | 542 | 16961 | 0.0192583 | 0.1693853 | 1.9764226 | 1.715381 |
| GO:0001756 | somitogenesis | 3 | 190 | 51 | 16961 | 0.0194406 | 0.1693853 | 5.2510836 | 1.7112913 |
| GO:0043524 | negative regulation of neuron apoptotic process | 5 | 190 | 139 | 16961 | 0.0201793 | 0.1693853 | 3.2110943 | 1.6950931 |
| GO:0007626 | locomotory behavior | 4 | 190 | 93 | 16961 | 0.0205778 | 0.1693853 | 3.839502 | 1.6866011 |
| GO:0000288 | nuclear-transcribed mRNA catabolic process, deadenylation-dependent decay | 3 | 190 | 53 | 16961 | 0.0215177 | 0.1693853 | 5.0529295 | 1.6672032 |
| GO:0016567 | protein ubiquitination | 11 | 190 | 487 | 16961 | 0.0215473 | 0.1693853 | 2.0163298 | 1.666608 |
| GO:0060123 | regulation of growth hormone secretion | 1 | 190 | 2 | 16961 | 0.0222795 | 0.1693853 | 44.634211 | 1.6520945 |
| GO:0072368 | regulation of lipid transport by negative regulation of transcription from RNA polymerase II promoter | 1 | 190 | 2 | 16961 | 0.0222795 | 0.1693853 | 44.634211 | 1.6520945 |
| GO:0045175 | basal protein localization | 1 | 190 | 2 | 16961 | 0.0222795 | 0.1693853 | 44.634211 | 1.6520945 |
| GO:2000191 | regulation of fatty acid transport | 1 | 190 | 2 | 16961 | 0.0222795 | 0.1693853 | 44.634211 | 1.6520945 |
| GO:2001295 | malonyl-CoA biosynthetic process | 1 | 190 | 2 | 16961 | 0.0222795 | 0.1693853 | 44.634211 | 1.6520945 |
| GO:2001045 | negative regulation of integrin-mediated signaling pathway | 1 | 190 | 2 | 16961 | 0.0222795 | 0.1693853 | 44.634211 | 1.6520945 |
| GO:0006212 | uracil catabolic process | 1 | 190 | 2 | 16961 | 0.0222795 | 0.1693853 | 44.634211 | 1.6520945 |
| GO:0051036 | regulation of endosome size | 1 | 190 | 2 | 16961 | 0.0222795 | 0.1693853 | 44.634211 | 1.6520945 |
| GO:1901264 | carbohydrate derivative transport | 1 | 190 | 2 | 16961 | 0.0222795 | 0.1693853 | 44.634211 | 1.6520945 |
| GO:1902117 | positive regulation of organelle assembly | 1 | 190 | 2 | 16961 | 0.0222795 | 0.1693853 | 44.634211 | 1.6520945 |
| GO:0030576 | Cajal body organization | 1 | 190 | 2 | 16961 | 0.0222795 | 0.1693853 | 44.634211 | 1.6520945 |
| GO:0006478 | peptidyl-tyrosine sulfation | 1 | 190 | 2 | 16961 | 0.0222795 | 0.1693853 | 44.634211 | 1.6520945 |
| GO:0019483 | beta-alanine biosynthetic process | 1 | 190 | 2 | 16961 | 0.0222795 | 0.1693853 | 44.634211 | 1.6520945 |
| GO:0019244 | lactate biosynthetic process from pyruvate | 1 | 190 | 2 | 16961 | 0.0222795 | 0.1693853 | 44.634211 | 1.6520945 |
| GO:0042264 | peptidyl-aspartic acid hydroxylation | 1 | 190 | 2 | 16961 | 0.0222795 | 0.1693853 | 44.634211 | 1.6520945 |
| GO:0031585 | regulation of inositol 1,4,5-trisphosphate-sensitive calcium-release channel activity | 1 | 190 | 2 | 16961 | 0.0222795 | 0.1693853 | 44.634211 | 1.6520945 |
| GO:0035507 | regulation of myosin-light-chain-phosphatase activity | 1 | 190 | 2 | 16961 | 0.0222795 | 0.1693853 | 44.634211 | 1.6520945 |
| GO:2000850 | negative regulation of glucocorticoid secretion | 1 | 190 | 2 | 16961 | 0.0222795 | 0.1693853 | 44.634211 | 1.6520945 |
| GO:0002638 | negative regulation of immunoglobulin production | 1 | 190 | 2 | 16961 | 0.0222795 | 0.1693853 | 44.634211 | 1.6520945 |
| GO:0010256 | endomembrane system organization | 1 | 190 | 2 | 16961 | 0.0222795 | 0.1693853 | 44.634211 | 1.6520945 |
| GO:2000401 | regulation of lymphocyte migration | 1 | 190 | 2 | 16961 | 0.0222795 | 0.1693853 | 44.634211 | 1.6520945 |
| GO:0090169 | regulation of spindle assembly | 1 | 190 | 2 | 16961 | 0.0222795 | 0.1693853 | 44.634211 | 1.6520945 |
| GO:0002684 | positive regulation of immune system process | 1 | 190 | 2 | 16961 | 0.0222795 | 0.1693853 | 44.634211 | 1.6520945 |
| GO:0060689 | cell differentiation involved in salivary gland development | 1 | 190 | 2 | 16961 | 0.0222795 | 0.1693853 | 44.634211 | 1.6520945 |
| GO:0071593 | lymphocyte aggregation | 1 | 190 | 2 | 16961 | 0.0222795 | 0.1693853 | 44.634211 | 1.6520945 |
| GO:1902031 | regulation of NADP metabolic process | 1 | 190 | 2 | 16961 | 0.0222795 | 0.1693853 | 44.634211 | 1.6520945 |
| GO:0038155 | interleukin-23-mediated signaling pathway | 1 | 190 | 2 | 16961 | 0.0222795 | 0.1693853 | 44.634211 | 1.6520945 |
| GO:0043519 | regulation of myosin II filament organization | 1 | 190 | 2 | 16961 | 0.0222795 | 0.1693853 | 44.634211 | 1.6520945 |
| GO:0039692 | single stranded viral RNA replication via double stranded DNA intermediate | 1 | 190 | 2 | 16961 | 0.0222795 | 0.1693853 | 44.634211 | 1.6520945 |
| GO:0021740 | principal sensory nucleus of trigeminal nerve development | 1 | 190 | 2 | 16961 | 0.0222795 | 0.1693853 | 44.634211 | 1.6520945 |
| GO:0032635 | interleukin-6 production | 1 | 190 | 2 | 16961 | 0.0222795 | 0.1693853 | 44.634211 | 1.6520945 |
| GO:0032785 | negative regulation of DNA-templated transcription, elongation | 1 | 190 | 2 | 16961 | 0.0222795 | 0.1693853 | 44.634211 | 1.6520945 |
| GO:0009785 | blue light signaling pathway | 1 | 190 | 2 | 16961 | 0.0222795 | 0.1693853 | 44.634211 | 1.6520945 |
| GO:0033206 | meiotic cytokinesis | 1 | 190 | 2 | 16961 | 0.0222795 | 0.1693853 | 44.634211 | 1.6520945 |
| GO:0002327 | immature B cell differentiation | 1 | 190 | 2 | 16961 | 0.0222795 | 0.1693853 | 44.634211 | 1.6520945 |
| GO:0090234 | regulation of kinetochore assembly | 1 | 190 | 2 | 16961 | 0.0222795 | 0.1693853 | 44.634211 | 1.6520945 |
| GO:2000584 | negative regulation of platelet-derived growth factor receptor-alpha signaling pathway | 1 | 190 | 2 | 16961 | 0.0222795 | 0.1693853 | 44.634211 | 1.6520945 |
| GO:0001894 | tissue homeostasis | 2 | 190 | 21 | 16961 | 0.0227952 | 0.1721271 | 8.5017544 | 1.6421563 |
| GO:0006338 | chromatin remodeling | 4 | 190 | 98 | 16961 | 0.0243962 | 0.1829712 | 3.643609 | 1.6126785 |
| GO:0031647 | regulation of protein stability | 3 | 190 | 56 | 16961 | 0.0248525 | 0.1829853 | 4.7822368 | 1.6046303 |
| GO:0034047 | regulation of protein phosphatase type 2A activity | 2 | 190 | 22 | 16961 | 0.0248926 | 0.1829853 | 8.115311 | 1.6039299 |
| GO:0048870 | cell motility | 2 | 190 | 22 | 16961 | 0.0248926 | 0.1829853 | 8.115311 | 1.6039299 |
| GO:0008361 | regulation of cell size | 2 | 190 | 23 | 16961 | 0.0270655 | 0.1974891 | 7.7624714 | 1.5675846 |
| GO:0030433 | ER-associated ubiquitin-dependent protein catabolic process | 3 | 190 | 58 | 16961 | 0.0272215 | 0.1974891 | 4.6173321 | 1.5650884 |
| GO:0030010 | establishment of cell polarity | 2 | 190 | 24 | 16961 | 0.0293119 | 0.2009631 | 7.4390351 | 1.5329563 |
| GO:0030833 | regulation of actin filament polymerization | 2 | 190 | 24 | 16961 | 0.0293119 | 0.2009631 | 7.4390351 | 1.5329563 |
| GO:0032922 | circadian regulation of gene expression | 3 | 190 | 60 | 16961 | 0.0297068 | 0.2009631 | 4.4634211 | 1.5271442 |
| GO:0046060 | dATP metabolic process | 1 | 190 | 3 | 16961 | 0.0332334 | 0.2009631 | 29.75614 | 1.4784254 |
| GO:1903244 | positive regulation of cardiac muscle hypertrophy in response to stress | 1 | 190 | 3 | 16961 | 0.0332334 | 0.2009631 | 29.75614 | 1.4784254 |
| GO:0006210 | thymine catabolic process | 1 | 190 | 3 | 16961 | 0.0332334 | 0.2009631 | 29.75614 | 1.4784254 |
| GO:0032959 | inositol trisphosphate biosynthetic process | 1 | 190 | 3 | 16961 | 0.0332334 | 0.2009631 | 29.75614 | 1.4784254 |
| GO:0043648 | dicarboxylic acid metabolic process | 1 | 190 | 3 | 16961 | 0.0332334 | 0.2009631 | 29.75614 | 1.4784254 |
| GO:0031664 | regulation of lipopolysaccharide-mediated signaling pathway | 1 | 190 | 3 | 16961 | 0.0332334 | 0.2009631 | 29.75614 | 1.4784254 |
| GO:0097676 | histone H3-K36 dimethylation | 1 | 190 | 3 | 16961 | 0.0332334 | 0.2009631 | 29.75614 | 1.4784254 |
| GO:0021553 | olfactory nerve development | 1 | 190 | 3 | 16961 | 0.0332334 | 0.2009631 | 29.75614 | 1.4784254 |
| GO:0031587 | positive regulation of inositol 1,4,5-trisphosphate-sensitive calcium-release channel activity | 1 | 190 | 3 | 16961 | 0.0332334 | 0.2009631 | 29.75614 | 1.4784254 |
| GO:0018193 | peptidyl-amino acid modification | 1 | 190 | 3 | 16961 | 0.0332334 | 0.2009631 | 29.75614 | 1.4784254 |
| GO:0035722 | interleukin-12-mediated signaling pathway | 1 | 190 | 3 | 16961 | 0.0332334 | 0.2009631 | 29.75614 | 1.4784254 |
| GO:0019065 | receptor-mediated endocytosis of virus by host cell | 1 | 190 | 3 | 16961 | 0.0332334 | 0.2009631 | 29.75614 | 1.4784254 |
| GO:2000681 | negative regulation of rubidium ion transport | 1 | 190 | 3 | 16961 | 0.0332334 | 0.2009631 | 29.75614 | 1.4784254 |
| GO:2000687 | negative regulation of rubidium ion transmembrane transporter activity | 1 | 190 | 3 | 16961 | 0.0332334 | 0.2009631 | 29.75614 | 1.4784254 |
| GO:1900024 | regulation of substrate adhesion-dependent cell spreading | 1 | 190 | 3 | 16961 | 0.0332334 | 0.2009631 | 29.75614 | 1.4784254 |
| GO:2000017 | positive regulation of determination of dorsal identity | 1 | 190 | 3 | 16961 | 0.0332334 | 0.2009631 | 29.75614 | 1.4784254 |
| GO:0016344 | meiotic chromosome movement towards spindle pole | 1 | 190 | 3 | 16961 | 0.0332334 | 0.2009631 | 29.75614 | 1.4784254 |
| GO:1990166 | protein localization to site of double-strand break | 1 | 190 | 3 | 16961 | 0.0332334 | 0.2009631 | 29.75614 | 1.4784254 |
| GO:0021988 | olfactory lobe development | 1 | 190 | 3 | 16961 | 0.0332334 | 0.2009631 | 29.75614 | 1.4784254 |
| GO:0006354 | DNA-templated transcription, elongation | 1 | 190 | 3 | 16961 | 0.0332334 | 0.2009631 | 29.75614 | 1.4784254 |
| GO:0043243 | positive regulation of protein complex disassembly | 1 | 190 | 3 | 16961 | 0.0332334 | 0.2009631 | 29.75614 | 1.4784254 |
| GO:0031281 | positive regulation of cyclase activity | 1 | 190 | 3 | 16961 | 0.0332334 | 0.2009631 | 29.75614 | 1.4784254 |
| GO:0033260 | nuclear DNA replication | 1 | 190 | 3 | 16961 | 0.0332334 | 0.2009631 | 29.75614 | 1.4784254 |
| GO:0070433 | negative regulation of nucleotide-binding oligomerization domain containing 2 signaling pathway | 1 | 190 | 3 | 16961 | 0.0332334 | 0.2009631 | 29.75614 | 1.4784254 |
| GO:0090045 | positive regulation of deacetylase activity | 1 | 190 | 3 | 16961 | 0.0332334 | 0.2009631 | 29.75614 | 1.4784254 |
| GO:0002331 | pre-B cell allelic exclusion | 1 | 190 | 3 | 16961 | 0.0332334 | 0.2009631 | 29.75614 | 1.4784254 |
| GO:0090481 | pyrimidine nucleotide-sugar transmembrane transport | 1 | 190 | 3 | 16961 | 0.0332334 | 0.2009631 | 29.75614 | 1.4784254 |
| GO:0045893 | positive regulation of transcription, DNA-templated | 12 | 190 | 588 | 16961 | 0.0333128 | 0.2009631 | 1.8218045 | 1.4773887 |
| GO:0007029 | endoplasmic reticulum organization | 2 | 190 | 26 | 16961 | 0.0340178 | 0.2030095 | 6.8668016 | 1.4682936 |
| GO:1900740 | positive regulation of protein insertion into mitochondrial membrane involved in apoptotic signaling pathway | 2 | 190 | 26 | 16961 | 0.0340178 | 0.2030095 | 6.8668016 | 1.4682936 |
| GO:0030168 | platelet activation | 6 | 190 | 216 | 16961 | 0.0349761 | 0.2067923 | 2.4796784 | 1.4562292 |
| GO:0001570 | vasculogenesis | 3 | 190 | 64 | 16961 | 0.0350243 | 0.2067923 | 4.1844572 | 1.4556307 |
| GO:0038095 | Fc-epsilon receptor signaling pathway | 8 | 190 | 337 | 16961 | 0.0363757 | 0.2090272 | 2.1191317 | 1.4391883 |
| GO:0048741 | skeletal muscle fiber development | 2 | 190 | 27 | 16961 | 0.0364736 | 0.2090272 | 6.6124756 | 1.4380212 |
| GO:0016050 | vesicle organization | 2 | 190 | 28 | 16961 | 0.0389956 | 0.2090272 | 6.3763158 | 1.4089849 |
| GO:0032321 | positive regulation of Rho GTPase activity | 4 | 190 | 115 | 16961 | 0.040417 | 0.2090272 | 3.1049886 | 1.3934361 |
| GO:0012501 | programmed cell death | 5 | 190 | 168 | 16961 | 0.0408519 | 0.2090272 | 2.6567982 | 1.3887879 |
| GO:0007163 | establishment or maintenance of cell polarity | 2 | 190 | 29 | 16961 | 0.0415819 | 0.2090272 | 6.1564428 | 1.381096 |
| GO:2000117 | negative regulation of cysteine-type endopeptidase activity | 1 | 190 | 4 | 16961 | 0.0440652 | 0.2090272 | 22.317105 | 1.3559044 |
| GO:0051653 | spindle localization | 1 | 190 | 4 | 16961 | 0.0440652 | 0.2090272 | 22.317105 | 1.3559044 |
| GO:0000052 | citrulline metabolic process | 1 | 190 | 4 | 16961 | 0.0440652 | 0.2090272 | 22.317105 | 1.3559044 |
| GO:0003383 | apical constriction | 1 | 190 | 4 | 16961 | 0.0440652 | 0.2090272 | 22.317105 | 1.3559044 |
| GO:0060318 | definitive erythrocyte differentiation | 1 | 190 | 4 | 16961 | 0.0440652 | 0.2090272 | 22.317105 | 1.3559044 |
| GO:0030540 | female genitalia development | 1 | 190 | 4 | 16961 | 0.0440652 | 0.2090272 | 22.317105 | 1.3559044 |
| GO:0051252 | regulation of RNA metabolic process | 1 | 190 | 4 | 16961 | 0.0440652 | 0.2090272 | 22.317105 | 1.3559044 |
| GO:0097466 | misfolded or incompletely synthesized glycoprotein catabolic process | 1 | 190 | 4 | 16961 | 0.0440652 | 0.2090272 | 22.317105 | 1.3559044 |
| GO:0006499 | N-terminal protein myristoylation | 1 | 190 | 4 | 16961 | 0.0440652 | 0.2090272 | 22.317105 | 1.3559044 |
| GO:0010715 | regulation of extracellular matrix disassembly | 1 | 190 | 4 | 16961 | 0.0440652 | 0.2090272 | 22.317105 | 1.3559044 |
| GO:0032970 | regulation of actin filament-based process | 1 | 190 | 4 | 16961 | 0.0440652 | 0.2090272 | 22.317105 | 1.3559044 |
| GO:0010961 | cellular magnesium ion homeostasis | 1 | 190 | 4 | 16961 | 0.0440652 | 0.2090272 | 22.317105 | 1.3559044 |
| GO:0007258 | JUN phosphorylation | 1 | 190 | 4 | 16961 | 0.0440652 | 0.2090272 | 22.317105 | 1.3559044 |
| GO:0008356 | asymmetric cell division | 1 | 190 | 4 | 16961 | 0.0440652 | 0.2090272 | 22.317105 | 1.3559044 |
| GO:0021545 | cranial nerve development | 1 | 190 | 4 | 16961 | 0.0440652 | 0.2090272 | 22.317105 | 1.3559044 |
| GO:0001920 | negative regulation of receptor recycling | 1 | 190 | 4 | 16961 | 0.0440652 | 0.2090272 | 22.317105 | 1.3559044 |
| GO:2000623 | negative regulation of nuclear-transcribed mRNA catabolic process, nonsense-mediated decay | 1 | 190 | 4 | 16961 | 0.0440652 | 0.2090272 | 22.317105 | 1.3559044 |
| GO:0060713 | labyrinthine layer morphogenesis | 1 | 190 | 4 | 16961 | 0.0440652 | 0.2090272 | 22.317105 | 1.3559044 |
| GO:0090116 | C-5 methylation of cytosine | 1 | 190 | 4 | 16961 | 0.0440652 | 0.2090272 | 22.317105 | 1.3559044 |
| GO:0071638 | negative regulation of monocyte chemotactic protein-1 production | 1 | 190 | 4 | 16961 | 0.0440652 | 0.2090272 | 22.317105 | 1.3559044 |
| GO:0072718 | response to cisplatin | 1 | 190 | 4 | 16961 | 0.0440652 | 0.2090272 | 22.317105 | 1.3559044 |
| GO:0060486 | Clara cell differentiation | 1 | 190 | 4 | 16961 | 0.0440652 | 0.2090272 | 22.317105 | 1.3559044 |
| GO:0002361 | CD4-positive, CD25-positive, alpha-beta regulatory T cell differentiation | 1 | 190 | 4 | 16961 | 0.0440652 | 0.2090272 | 22.317105 | 1.3559044 |
| GO:0034184 | positive regulation of maintenance of mitotic sister chromatid cohesion | 1 | 190 | 4 | 16961 | 0.0440652 | 0.2090272 | 22.317105 | 1.3559044 |
| GO:2000021 | regulation of ion homeostasis | 1 | 190 | 4 | 16961 | 0.0440652 | 0.2090272 | 22.317105 | 1.3559044 |
| GO:0072201 | negative regulation of mesenchymal cell proliferation | 1 | 190 | 4 | 16961 | 0.0440652 | 0.2090272 | 22.317105 | 1.3559044 |
| GO:0031936 | negative regulation of chromatin silencing | 1 | 190 | 4 | 16961 | 0.0440652 | 0.2090272 | 22.317105 | 1.3559044 |
| GO:0019805 | quinolinate biosynthetic process | 1 | 190 | 4 | 16961 | 0.0440652 | 0.2090272 | 22.317105 | 1.3559044 |
| GO:0097368 | establishment of Sertoli cell barrier | 1 | 190 | 4 | 16961 | 0.0440652 | 0.2090272 | 22.317105 | 1.3559044 |
| GO:0045716 | positive regulation of low-density lipoprotein particle receptor biosynthetic process | 1 | 190 | 4 | 16961 | 0.0440652 | 0.2090272 | 22.317105 | 1.3559044 |
| GO:0001915 | negative regulation of T cell mediated cytotoxicity | 1 | 190 | 4 | 16961 | 0.0440652 | 0.2090272 | 22.317105 | 1.3559044 |
| GO:1903347 | negative regulation of tight junction assembly | 1 | 190 | 4 | 16961 | 0.0440652 | 0.2090272 | 22.317105 | 1.3559044 |
| GO:0043242 | negative regulation of protein complex disassembly | 1 | 190 | 4 | 16961 | 0.0440652 | 0.2090272 | 22.317105 | 1.3559044 |
| GO:0042159 | lipoprotein catabolic process | 1 | 190 | 4 | 16961 | 0.0440652 | 0.2090272 | 22.317105 | 1.3559044 |
| GO:0002904 | positive regulation of B cell apoptotic process | 1 | 190 | 4 | 16961 | 0.0440652 | 0.2090272 | 22.317105 | 1.3559044 |
| GO:0006084 | acetyl-CoA metabolic process | 1 | 190 | 4 | 16961 | 0.0440652 | 0.2090272 | 22.317105 | 1.3559044 |
| GO:0010587 | miRNA catabolic process | 1 | 190 | 4 | 16961 | 0.0440652 | 0.2090272 | 22.317105 | 1.3559044 |
| GO:2000973 | regulation of pro-B cell differentiation | 1 | 190 | 4 | 16961 | 0.0440652 | 0.2090272 | 22.317105 | 1.3559044 |
| GO:0008298 | intracellular mRNA localization | 1 | 190 | 4 | 16961 | 0.0440652 | 0.2090272 | 22.317105 | 1.3559044 |
| GO:2000330 | positive regulation of T-helper 17 cell lineage commitment | 1 | 190 | 4 | 16961 | 0.0440652 | 0.2090272 | 22.317105 | 1.3559044 |
| GO:0006888 | ER to Golgi vesicle-mediated transport | 3 | 190 | 71 | 16961 | 0.0454256 | 0.2145633 | 3.7719051 | 1.3426997 |
| GO:0046777 | protein autophosphorylation | 5 | 190 | 175 | 16961 | 0.0472443 | 0.217927 | 2.5505263 | 1.3256508 |
| GO:0007032 | endosome organization | 2 | 190 | 32 | 16961 | 0.0497097 | 0.217927 | 5.5792763 | 1.3035585 |
| GO:0043392 | negative regulation of DNA binding | 2 | 190 | 32 | 16961 | 0.0497097 | 0.217927 | 5.5792763 | 1.3035585 |
