# Supplementary material for: Expression Profiles and Functional Analysis of Plasma Exosomal Circular RNAs in Acute Myocardial Infarction
Source: Biomed Res Int. 2022 Oct 1;2022:3458227. doi: 10.1155/2022/3458227 (PMC9547997; doi:10.1155/2022/3458227)
Supplement: Supplementary 8 — Supplementary Table S8: Pathway enrichment analyses of the differentially expressed exosomal circRNAs in comparison of AMI and control. [file 3458227.f8.docx]

Supplementary Table S8 Pathway enrichment analyses of the differentially expressed exosomal circRNAs in comparison of AMI and control.

| PathwayID | PathwayTerm | DifGene | AllDifGene | GeneInPathway | AllGene | P-Value | FDR | Enrichment | (-log10P) |
| --- | --- | --- | --- | --- | --- | --- | --- | --- | --- |
| PATH:04520 | Adherens junction | 6 | 74 | 73 | 6782 | 0.0001312 | 0.016063 | 7.5327656 | 3.8819284 |
| PATH:00310 | Lysine degradation | 5 | 74 | 51 | 6782 | 0.0002144 | 0.016063 | 8.9851616 | 3.6687488 |
| PATH:04919 | Thyroid hormone signaling pathway | 7 | 74 | 119 | 6782 | 0.0002921 | 0.016063 | 5.391097 | 3.5345365 |
| PATH:04010 | MAPK signaling pathway | 10 | 74 | 275 | 6782 | 0.0007365 | 0.0303813 | 3.3326781 | 3.1328178 |
| PATH:04918 | Thyroid hormone synthesis | 5 | 74 | 72 | 6782 | 0.0010693 | 0.0352867 | 6.3644895 | 2.9709024 |
| PATH:04728 | Dopaminergic synapse | 6 | 74 | 131 | 6782 | 0.0029294 | 0.080559 | 4.197648 | 2.5332185 |
| PATH:04915 | Estrogen signaling pathway | 5 | 74 | 100 | 6782 | 0.0045449 | 0.1071301 | 4.5824324 | 2.3424746 |
| PATH:04720 | Long-term potentiation | 4 | 74 | 70 | 6782 | 0.0069582 | 0.1411633 | 5.2370656 | 2.1575047 |
| PATH:05166 | HTLV-I infection | 8 | 74 | 264 | 6782 | 0.0077239 | 0.1411633 | 2.7772318 | 2.1121658 |
| PATH:04370 | VEGF signaling pathway | 4 | 74 | 79 | 6782 | 0.010596 | 0.1411633 | 4.6404379 | 1.9748594 |
| PATH:05205 | Proteoglycans in cancer | 7 | 74 | 225 | 6782 | 0.0109954 | 0.1411633 | 2.8512913 | 1.9587877 |
| PATH:04973 | Carbohydrate digestion and absorption | 3 | 74 | 45 | 6782 | 0.0127469 | 0.1411633 | 6.1099099 | 1.894596 |
| PATH:05202 | Transcriptional misregulation in cancer | 6 | 74 | 180 | 6782 | 0.0134006 | 0.1411633 | 3.054955 | 1.872876 |
| PATH:05132 | Salmonella infection | 4 | 74 | 86 | 6782 | 0.0141474 | 0.1411633 | 4.2627278 | 1.8493246 |
| PATH:04012 | ErbB signaling pathway | 4 | 74 | 87 | 6782 | 0.0147092 | 0.1411633 | 4.213731 | 1.8324098 |
| PATH:04068 | FoxO signaling pathway | 5 | 74 | 133 | 6782 | 0.014716 | 0.1411633 | 3.4454379 | 1.8322106 |
| PATH:04210 | Apoptosis | 4 | 74 | 90 | 6782 | 0.0164792 | 0.1411633 | 4.0732733 | 1.7830638 |
| PATH:05160 | Hepatitis C | 5 | 74 | 137 | 6782 | 0.0165428 | 0.1411633 | 3.3448412 | 1.7813913 |
| PATH:04062 | Chemokine signaling pathway | 6 | 74 | 191 | 6782 | 0.0175072 | 0.1411633 | 2.8790151 | 1.7567824 |
| PATH:04713 | Circadian entrainment | 4 | 74 | 97 | 6782 | 0.0211137 | 0.1411633 | 3.7793257 | 1.6754353 |
| PATH:05161 | Hepatitis B | 5 | 74 | 146 | 6782 | 0.0211926 | 0.1411633 | 3.1386524 | 1.6738156 |
| PATH:05169 | Epstein-Barr virus infection | 6 | 74 | 201 | 6782 | 0.0219316 | 0.1411633 | 2.7357806 | 1.6589297 |
| PATH:05223 | Non-small cell lung cancer | 3 | 74 | 56 | 6782 | 0.0228728 | 0.1411633 | 4.909749 | 1.6406799 |
| PATH:04261 | Adrenergic signaling in cardiomyocytes | 5 | 74 | 149 | 6782 | 0.0229148 | 0.1411633 | 3.075458 | 1.6398842 |
| PATH:05221 | Acute myeloid leukemia | 3 | 74 | 57 | 6782 | 0.0239583 | 0.1411633 | 4.8236131 | 1.6205432 |
| PATH:04916 | Melanogenesis | 4 | 74 | 101 | 6782 | 0.0240881 | 0.1411633 | 3.6296495 | 1.6181981 |
| PATH:05203 | Viral carcinogenesis | 6 | 74 | 207 | 6782 | 0.0249222 | 0.1411633 | 2.6564826 | 1.6034132 |
| PATH:04510 | Focal adhesion | 6 | 74 | 207 | 6782 | 0.0249222 | 0.1411633 | 2.6564826 | 1.6034132 |
| PATH:04310 | Wnt signaling pathway | 5 | 74 | 153 | 6782 | 0.0253494 | 0.1411633 | 2.9950539 | 1.5960327 |
| PATH:04723 | Retrograde endocannabinoid signaling | 4 | 74 | 103 | 6782 | 0.0256661 | 0.1411633 | 3.5591708 | 1.5906407 |
| PATH:04066 | HIF-1 signaling pathway | 4 | 74 | 106 | 6782 | 0.0281479 | 0.1498196 | 3.4584396 | 1.5505536 |
| PATH:05131 | Shigellosis | 3 | 74 | 62 | 6782 | 0.0297993 | 0.1526607 | 4.434612 | 1.5257933 |
| PATH:04810 | Regulation of actin cytoskeleton | 6 | 74 | 218 | 6782 | 0.0310943 | 0.1526607 | 2.5224399 | 1.5073189 |
| PATH:04150 | mTOR signaling pathway | 3 | 74 | 64 | 6782 | 0.032328 | 0.1526607 | 4.2960304 | 1.4904212 |
| PATH:00300 | Lysine biosynthesis | 1 | 74 | 3 | 6782 | 0.0323826 | 0.1526607 | 30.54955 | 1.4896888 |
| PATH:05214 | Glioma | 3 | 74 | 65 | 6782 | 0.0336333 | 0.1537689 | 4.2299376 | 1.4732307 |
| PATH:04725 | Cholinergic synapse | 4 | 74 | 113 | 6782 | 0.0344815 | 0.1537689 | 3.2442 | 1.4624136 |
| PATH:04141 | Protein processing in endoplasmic reticulum | 5 | 74 | 168 | 6782 | 0.0359384 | 0.1558429 | 2.7276384 | 1.4444416 |
| PATH:04014 | Ras signaling pathway | 6 | 74 | 227 | 6782 | 0.0368356 | 0.1558429 | 2.4224312 | 1.4337323 |
| PATH:05031 | Amphetamine addiction | 3 | 74 | 70 | 6782 | 0.0405659 | 0.1632529 | 3.9277992 | 1.3918393 |
| PATH:05211 | Renal cell carcinoma | 3 | 74 | 70 | 6782 | 0.0405659 | 0.1632529 | 3.9277992 | 1.3918393 |
| PATH:05164 | Influenza A | 5 | 74 | 175 | 6782 | 0.0416946 | 0.1636995 | 2.6185328 | 1.37992 |
| PATH:04270 | Vascular smooth muscle contraction | 4 | 74 | 121 | 6782 | 0.0426611 | 0.1636995 | 3.0297074 | 1.3699681 |
| PATH:04110 | Cell cycle | 4 | 74 | 124 | 6782 | 0.0459887 | 0.1724576 | 2.956408 | 1.3373489 |
| PATH:04662 | B cell receptor signaling pathway | 3 | 74 | 76 | 6782 | 0.0497634 | 0.1789094 | 3.6177098 | 1.3030899 |
| PATH:05168 | Herpes simplex infection | 5 | 74 | 184 | 6782 | 0.0498778 | 0.1789094 | 2.4904524 | 1.302093 |
