# Supplementary material for: Expression Profiles and Functional Analysis of Plasma Exosomal Circular RNAs in Acute Myocardial Infarction
Source: Biomed Res Int. 2022 Oct 1;2022:3458227. doi: 10.1155/2022/3458227 (PMC9547997; doi:10.1155/2022/3458227)
Supplement: Supplementary 9 — Supplementary Table S9: Pathway enrichment analyses of the differentially expressed exosomal circRNAs in comparison of CAD and control. [file 3458227.f9.docx]

Supplementary Table S9 Pathway enrichment analyses of the differentially expressed exosomal circRNAs in comparison of CAD and control.

| PathwayID | PathwayTerm | DifGene | AllDifGene | GeneInPathway | AllGene | P-Value | FDR | Enrichment | (-log10P) |
| --- | --- | --- | --- | --- | --- | --- | --- | --- | --- |
| PATH:00310 | Lysine degradation | 5 | 67 | 51 | 6782 | 0.0001337 | 0.0131236 | 9.9239099 | 3.8737454 |
| PATH:05223 | Non-small cell lung cancer | 5 | 67 | 56 | 6782 | 0.0002093 | 0.0131236 | 9.0378465 | 3.6791321 |
| PATH:04064 | NF-kappa B signaling pathway | 6 | 67 | 91 | 6782 | 0.0002557 | 0.0131236 | 6.674102 | 3.5923456 |
| PATH:04066 | HIF-1 signaling pathway | 6 | 67 | 106 | 6782 | 0.0005828 | 0.0183664 | 5.7296536 | 3.2344703 |
| PATH:04720 | Long-term potentiation | 5 | 67 | 70 | 6782 | 0.0005963 | 0.0183664 | 7.2302772 | 3.2245264 |
| PATH:04662 | B cell receptor signaling pathway | 5 | 67 | 76 | 6782 | 0.0008697 | 0.0223221 | 6.6594658 | 3.0606342 |
| PATH:04070 | Phosphatidylinositol signaling system | 5 | 67 | 81 | 6782 | 0.0011612 | 0.0246415 | 6.2483877 | 2.9350929 |
| PATH:04664 | Fc epsilon RI signaling pathway | 5 | 67 | 83 | 6782 | 0.0012961 | 0.0246415 | 6.0978241 | 2.8873769 |
| PATH:05205 | Proteoglycans in cancer | 8 | 67 | 225 | 6782 | 0.0015628 | 0.0246415 | 3.5990713 | 2.8061076 |
| PATH:04012 | ErbB signaling pathway | 5 | 67 | 87 | 6782 | 0.0016001 | 0.0246415 | 5.8174644 | 2.795853 |
| PATH:04611 | Platelet activation | 6 | 67 | 131 | 6782 | 0.0017612 | 0.0246573 | 4.6362083 | 2.7541827 |
| PATH:04666 | Fc gamma R-mediated phagocytosis | 5 | 67 | 95 | 6782 | 0.0023615 | 0.0303058 | 5.3275727 | 2.6268142 |
| PATH:04150 | mTOR signaling pathway | 4 | 67 | 64 | 6782 | 0.0035431 | 0.0412244 | 6.3264925 | 2.4506214 |
| PATH:05214 | Glioma | 4 | 67 | 65 | 6782 | 0.0037477 | 0.0412244 | 6.2291619 | 2.4262383 |
| PATH:05203 | Viral carcinogenesis | 7 | 67 | 207 | 6782 | 0.0041055 | 0.0415693 | 3.4230298 | 2.3866353 |
| PATH:04668 | TNF signaling pathway | 5 | 67 | 110 | 6782 | 0.0044531 | 0.0415693 | 4.6010855 | 2.3513359 |
| PATH:05211 | Renal cell carcinoma | 4 | 67 | 70 | 6782 | 0.0048919 | 0.0415693 | 5.7842217 | 2.310524 |
| PATH:04918 | Thyroid hormone synthesis | 4 | 67 | 72 | 6782 | 0.0054087 | 0.0415693 | 5.6235489 | 2.2669079 |
| PATH:04730 | Long-term depression | 4 | 67 | 73 | 6782 | 0.0056803 | 0.0415693 | 5.546514 | 2.2456285 |
| PATH:04520 | Adherens junction | 4 | 67 | 73 | 6782 | 0.0056803 | 0.0415693 | 5.546514 | 2.2456285 |
| PATH:04022 | cGMP-PKG signaling pathway | 6 | 67 | 167 | 6782 | 0.0058799 | 0.0415693 | 3.6367861 | 2.2306286 |
| PATH:04141 | Protein processing in endoplasmic reticulum | 6 | 67 | 168 | 6782 | 0.0060511 | 0.0415693 | 3.6151386 | 2.2181653 |
| PATH:04919 | Thyroid hormone signaling pathway | 5 | 67 | 119 | 6782 | 0.0062084 | 0.0415693 | 4.2531042 | 2.2070203 |
| PATH:04270 | Vascular smooth muscle contraction | 5 | 67 | 121 | 6782 | 0.0066563 | 0.0416874 | 4.182805 | 2.1767647 |
| PATH:04014 | Ras signaling pathway | 7 | 67 | 227 | 6782 | 0.0067674 | 0.0416874 | 3.1214413 | 2.1695756 |
| PATH:04370 | VEGF signaling pathway | 4 | 67 | 79 | 6782 | 0.007503 | 0.0444409 | 5.1252598 | 2.1247642 |
| PATH:05206 | MicroRNAs in cancer | 8 | 67 | 296 | 6782 | 0.0083112 | 0.0474048 | 2.7357806 | 2.0803349 |
| PATH:04728 | Dopaminergic synapse | 5 | 67 | 131 | 6782 | 0.0092412 | 0.0508264 | 3.8635069 | 2.0342731 |
| PATH:05215 | Prostate cancer | 4 | 67 | 89 | 6782 | 0.0113304 | 0.0596026 | 4.5493879 | 1.9457533 |
| PATH:04330 | Notch signaling pathway | 3 | 67 | 48 | 6782 | 0.0116109 | 0.0596026 | 6.3264925 | 1.9351343 |
| PATH:05161 | Hepatitis B | 5 | 67 | 146 | 6782 | 0.0143086 | 0.0694318 | 3.4665713 | 1.8444023 |
| PATH:05213 | Endometrial cancer | 3 | 67 | 52 | 6782 | 0.0144274 | 0.0694318 | 5.8398393 | 1.8408126 |
| PATH:04510 | Focal adhesion | 6 | 67 | 207 | 6782 | 0.015981 | 0.0735411 | 2.9340255 | 1.7963968 |
| PATH:04750 | Inflammatory mediator regulation of TRP channels | 4 | 67 | 99 | 6782 | 0.0162363 | 0.0735411 | 4.0898538 | 1.7895117 |
| PATH:04915 | Estrogen signaling pathway | 4 | 67 | 100 | 6782 | 0.0167898 | 0.0738749 | 4.0489552 | 1.7749556 |
| PATH:04723 | Retrograde endocannabinoid signaling | 4 | 67 | 103 | 6782 | 0.0185207 | 0.0792274 | 3.9310245 | 1.7323429 |
| PATH:04921 | Oxytocin signaling pathway | 5 | 67 | 158 | 6782 | 0.0195118 | 0.0812113 | 3.2032874 | 1.7097023 |
| PATH:04320 | Dorso-ventral axis formation | 2 | 67 | 24 | 6782 | 0.0230711 | 0.0934986 | 8.4353234 | 1.6369319 |
| PATH:04725 | Cholinergic synapse | 4 | 67 | 113 | 6782 | 0.0250759 | 0.0990175 | 3.5831462 | 1.6007441 |
| PATH:05164 | Influenza A | 5 | 67 | 175 | 6782 | 0.0288161 | 0.1102405 | 2.8921109 | 1.5403642 |
| PATH:00300 | Lysine biosynthesis | 1 | 67 | 3 | 6782 | 0.0293497 | 0.1102405 | 33.741294 | 1.5323958 |
| PATH:05212 | Pancreatic cancer | 3 | 67 | 70 | 6782 | 0.0315348 | 0.1156277 | 4.3381663 | 1.5012094 |
| PATH:04020 | Calcium signaling pathway | 5 | 67 | 185 | 6782 | 0.0354184 | 0.1268472 | 2.7357806 | 1.4507713 |
| PATH:04722 | Neurotrophin signaling pathway | 4 | 67 | 130 | 6782 | 0.0391002 | 0.1368506 | 3.1145809 | 1.4078212 |
| PATH:04068 | FoxO signaling pathway | 4 | 67 | 133 | 6782 | 0.0419603 | 0.1415302 | 3.0443272 | 1.3771615 |
| PATH:04530 | Tight junction | 4 | 67 | 134 | 6782 | 0.0429395 | 0.1415302 | 3.0216084 | 1.3671429 |
| PATH:05200 | Pathways in cancer | 7 | 67 | 330 | 6782 | 0.0431943 | 0.1415302 | 2.1471732 | 1.3645737 |
| PATH:05166 | HTLV-I infection | 6 | 67 | 264 | 6782 | 0.0452752 | 0.1452581 | 2.3005427 | 1.3441392 |
| PATH:05169 | Epstein-Barr virus infection | 5 | 67 | 201 | 6782 | 0.0478054 | 0.1502455 | 2.518007 | 1.3205231 |
| PATH:04130 | SNARE interactions in vesicular transport | 2 | 67 | 36 | 6782 | 0.0488361 | 0.1504152 | 5.6235489 | 1.311259 |
