# Supplementary material for: Expression Profiles and Functional Analysis of Plasma Exosomal Circular RNAs in Acute Myocardial Infarction
Source: Biomed Res Int. 2022 Oct 1;2022:3458227. doi: 10.1155/2022/3458227 (PMC9547997; doi:10.1155/2022/3458227)
Supplement: Supplementary 10 — Supplementary Table S10: Pathway enrichment analyses of the differentially expressed exosomal circRNAs in comparison of AMI and CAD. [file 3458227.f10.docx]

Supplementary Table S10 Pathway enrichment analyses of the differentially expressed exosomal circRNAs in comparison of AMI and CAD.

| PathwayID | PathwayTerm | DifGene | AllDifGene | GeneInPathway | AllGene | P-Value | FDR | Enrichment | (-log10P) |
| --- | --- | --- | --- | --- | --- | --- | --- | --- | --- |
| PATH:00310 | Lysine degradation | 5 | 70 | 51 | 6782 | 0.0001648 | 0.0220806 | 9.4985994 | 3.783093 |
| PATH:04360 | Axon guidance | 6 | 70 | 131 | 6782 | 0.0022076 | 0.1479067 | 4.4375136 | 2.656087 |
| PATH:04921 | Oxytocin signaling pathway | 6 | 70 | 158 | 6782 | 0.0055785 | 0.1518655 | 3.6792043 | 2.2534834 |
| PATH:04720 | Long-term potentiation | 4 | 70 | 70 | 6782 | 0.0057181 | 0.1518655 | 5.5363265 | 2.242745 |
| PATH:04670 | Leukocyte transendothelial migration | 5 | 70 | 118 | 6782 | 0.0072074 | 0.1518655 | 4.1053269 | 2.1422208 |
| PATH:03018 | RNA degradation | 4 | 70 | 77 | 6782 | 0.007997 | 0.1518655 | 5.0330241 | 2.0970743 |
| PATH:04370 | VEGF signaling pathway | 4 | 70 | 79 | 6782 | 0.0087434 | 0.1518655 | 4.9056058 | 2.0583221 |
| PATH:00620 | Pyruvate metabolism | 3 | 70 | 42 | 6782 | 0.0090666 | 0.1518655 | 6.9204082 | 2.0425556 |
| PATH:04728 | Dopaminergic synapse | 5 | 70 | 131 | 6782 | 0.0110635 | 0.1647239 | 3.697928 | 1.9561057 |
| PATH:04210 | Apoptosis | 4 | 70 | 90 | 6782 | 0.0136656 | 0.1727973 | 4.3060317 | 1.8643704 |
| PATH:03015 | mRNA surveillance pathway | 4 | 70 | 91 | 6782 | 0.0141849 | 0.1727973 | 4.2587127 | 1.8481752 |
| PATH:05014 | Amyotrophic lateral sclerosis (ALS) | 3 | 70 | 54 | 6782 | 0.0179452 | 0.1798735 | 5.3825397 | 1.7460506 |
| PATH:05166 | HTLV-I infection | 7 | 70 | 264 | 6782 | 0.0185265 | 0.1798735 | 2.5689394 | 1.732206 |
| PATH:04750 | Inflammatory mediator regulation of TRP channels | 4 | 70 | 99 | 6782 | 0.0187927 | 0.1798735 | 3.9145743 | 1.7260097 |
| PATH:04010 | MAPK signaling pathway | 7 | 70 | 275 | 6782 | 0.0226286 | 0.2021486 | 2.4661818 | 1.6453429 |
| PATH:04114 | Oocyte meiosis | 4 | 70 | 112 | 6782 | 0.0280789 | 0.2173111 | 3.4602041 | 1.5516197 |
| PATH:04022 | cGMP-PKG signaling pathway | 5 | 70 | 167 | 6782 | 0.0285449 | 0.2173111 | 2.9007699 | 1.5444707 |
| PATH:04141 | Protein processing in endoplasmic reticulum | 5 | 70 | 168 | 6782 | 0.029191 | 0.2173111 | 2.8835034 | 1.5347504 |
| PATH:04919 | Thyroid hormone signaling pathway | 4 | 70 | 119 | 6782 | 0.0340386 | 0.2400619 | 3.2566627 | 1.4680279 |
| PATH:04270 | Vascular smooth muscle contraction | 4 | 70 | 121 | 6782 | 0.0358672 | 0.2403105 | 3.2028335 | 1.4453021 |
| PATH:05202 | Transcriptional misregulation in cancer | 5 | 70 | 180 | 6782 | 0.0376754 | 0.2404051 | 2.6912698 | 1.4239418 |
| PATH:04662 | B cell receptor signaling pathway | 3 | 70 | 76 | 6782 | 0.0433623 | 0.2616346 | 3.8244361 | 1.3628879 |
| PATH:04611 | Platelet activation | 4 | 70 | 131 | 6782 | 0.0458557 | 0.2616346 | 2.9583424 | 1.3386071 |
| PATH:04380 | Osteoclast differentiation | 4 | 70 | 133 | 6782 | 0.0480227 | 0.2616346 | 2.9138561 | 1.3185535 |
| PATH:04530 | Tight junction | 4 | 70 | 134 | 6782 | 0.0491273 | 0.2616346 | 2.8921109 | 1.3086767 |
