# Supplementary material for: Expression Profiles and Functional Analysis of Plasma Exosomal Circular RNAs in Acute Myocardial Infarction
Source: Biomed Res Int. 2022 Oct 1;2022:3458227. doi: 10.1155/2022/3458227 (PMC9547997; doi:10.1155/2022/3458227)
Supplement: Supplementary 11 — Supplementary Table S11: Cardiovascular disease-associated microRNAs which interacted with differentially expressed exosomal circRNAs and their potential targets in comparison of AMI and control. [file 3458227.f11.docx]

Supplementary Table S11 Cardiovascular disease-associated microRNAs which interacted with differentially expressed exosomal circRNAs and their potential targets in comparison of AMI and control.

| miRNAs | Target Gene symbols |
| --- | --- |
| hsa-miR-205-5p | ZNF800 |
| hsa-miR-145-5p | ZNF423 |
| hsa-miR-145-5p | ZFYVE9 |
| hsa-miR-145-5p | ZDHHC9 |
| hsa-miR-217 | ZCCHC2 |
| hsa-miR-145-5p | ZBTB10 |
| hsa-miR-217 | YWHAG |
| hsa-miR-145-5p | YTHDF2 |
| hsa-miR-217 | YTHDC1 |
| hsa-miR-145-5p | YTHDC1 |
| hsa-miR-145-5p | XRN1 |
| hsa-miR-205-5p | WDR48 |
| hsa-miR-217 | WAPAL |
| hsa-miR-217 | VSNL1 |
| hsa-miR-205-5p | VASN |
| hsa-miR-145-5p | VASN |
| hsa-miR-145-5p | UXS1 |
| hsa-miR-145-5p | USP46 |
| hsa-miR-217 | UBL3 |
| hsa-miR-145-5p | UBA6 |
| hsa-miR-205-5p | TSC22D1 |
| hsa-miR-145-5p | TRIM2 |
| hsa-miR-205-5p | TRAK2 |
| hsa-miR-217 | TMTC4 |
| hsa-miR-145-5p | TM9SF4 |
| hsa-miR-145-5p | TLN2 |
| hsa-miR-145-5p | TIRAP |
| hsa-miR-145-5p | TBPL1 |
| hsa-miR-217 | TBC1D15 |
| hsa-miR-217 | TACC2 |
| hsa-miR-217 | TACC1 |
| hsa-miR-217 | STX1A |
| hsa-miR-217 | STT3A |
| hsa-miR-217 | STAG2 |
| hsa-miR-217 | ST18 |
| hsa-miR-145-5p | SRGAP1 |
| hsa-miR-205-5p | SQLE |
| hsa-miR-145-5p | SPSB4 |
| hsa-miR-217 | SPOPL |
| hsa-miR-145-5p | SPATS2 |
| hsa-miR-205-5p | SORBS1 |
| hsa-miR-205-5p | SNX27 |
| hsa-miR-145-5p | SNX27 |
| hsa-miR-205-5p | SMG7 |
| hsa-miR-145-5p | SMAD3 |
| hsa-miR-145-5p | SLITRK6 |
| hsa-miR-145-5p | SLITRK4 |
| hsa-miR-217 | SLC38A2 |
| hsa-miR-217 | SLC31A1 |
| hsa-miR-145-5p | SLC25A25 |
| hsa-miR-217 | SIRT1 |
| hsa-miR-205-5p | SIAH1 |
| hsa-miR-205-5p | SGMS1 |
| hsa-miR-217 | SENP7 |
| hsa-miR-145-5p | SEMA6A |
| hsa-miR-145-5p | SEMA3A |
| hsa-miR-205-5p | SEL1L |
| hsa-miR-205-5p | SCMH1 |
| hsa-miR-145-5p | SCAMP3 |
| hsa-miR-205-5p | SBF2 |
| hsa-miR-145-5p | SBF2 |
| hsa-miR-205-5p | SATB2 |
| hsa-miR-145-5p | SACM1L |
| hsa-miR-205-5p | RTN3 |
| hsa-miR-145-5p | RTKN |
| hsa-miR-217 | RTF1 |
| hsa-miR-145-5p | RSPO1 |
| hsa-miR-145-5p | RNF216 |
| hsa-miR-217 | RIN2 |
| hsa-miR-145-5p | RIN2 |
| hsa-miR-145-5p | RGS7 |
| hsa-miR-145-5p | REV3L |
| hsa-miR-145-5p | REEP1 |
| hsa-miR-205-5p | RBM47 |
| hsa-miR-217 | RBM39 |
| hsa-miR-145-5p | RASA1 |
| hsa-miR-145-5p | RAPH1 |
| hsa-miR-217 | RAP2C |
| hsa-miR-145-5p | RAB14 |
| hsa-miR-205-5p | RAB11FIP1 |
| hsa-miR-205-5p | QKI |
| hsa-miR-145-5p | PXN |
| hsa-miR-145-5p | PTGFR |
| hsa-miR-217 | PSMF1 |
| hsa-miR-145-5p | PSD3 |
| hsa-miR-205-5p | PRKCE |
| hsa-miR-145-5p | PPP3CA |
| hsa-miR-205-5p | PPP1R15B |
| hsa-miR-217 | PPM1D |
| hsa-miR-145-5p | PLCL2 |
| hsa-miR-145-5p | PLCE1 |
| hsa-miR-205-5p | PLCB1 |
| hsa-miR-205-5p | PJA2 |
| hsa-miR-205-5p | PHC2 |
| hsa-miR-217 | PDS5B |
| hsa-miR-205-5p | PDE3B |
| hsa-miR-145-5p | PDCD4 |
| hsa-miR-217 | PCNA |
| hsa-miR-145-5p | PAN2 |
| hsa-miR-145-5p | NUFIP2 |
| hsa-miR-145-5p | NUAK1 |
| hsa-miR-217 | NR4A2 |
| hsa-miR-205-5p | NR3C2 |
| hsa-miR-217 | NOVA1 |
| hsa-miR-217 | NIPBL |
| hsa-miR-205-5p | NFAT5 |
| hsa-miR-145-5p | NET1 |
| hsa-miR-145-5p | NEDD9 |
| hsa-miR-205-5p | NECAP1 |
| hsa-miR-205-5p | NDUFA4 |
| hsa-miR-145-5p | NAA50 |
| hsa-miR-145-5p | MYO5A |
| hsa-miR-217 | MYEF2 |
| hsa-miR-205-5p | MSL2 |
| hsa-miR-145-5p | MPZL2 |
| hsa-miR-145-5p | MPP5 |
| hsa-miR-205-5p | MMD |
| hsa-miR-145-5p | MKL2 |
| hsa-miR-217 | MIER3 |
| hsa-miR-205-5p | MGRN1 |
| hsa-miR-205-5p | MED1 |
| hsa-miR-145-5p | MDFIC |
| hsa-miR-205-5p | MARCKS |
| hsa-miR-217 | MAPK8IP1 |
| hsa-miR-145-5p | MAP4K4 |
| hsa-miR-145-5p | MAP3K3 |
| hsa-miR-145-5p | LRRC16A |
| hsa-miR-205-5p | LRP1 |
| hsa-miR-205-5p | LPAR1 |
| hsa-miR-145-5p | LOX |
| hsa-miR-205-5p | LIN9 |
| hsa-miR-217 | LIN9 |
| hsa-miR-145-5p | LENG8 |
| hsa-miR-205-5p | LCOR |
| hsa-miR-217 | LCOR |
| hsa-miR-205-5p | LAMC1 |
| hsa-miR-217 | KRAS |
| hsa-miR-205-5p | KLF12 |
| hsa-miR-145-5p | KIF21A |
| hsa-miR-145-5p | KIAA0355 |
| hsa-miR-145-5p | KDM2B |
| hsa-miR-217 | KCNH5 |
| hsa-miR-205-5p | KAT2B |
| hsa-miR-145-5p | IVNS1ABP |
| hsa-miR-145-5p | IRS1 |
| hsa-miR-145-5p | INO80 |
| hsa-miR-205-5p | HSD17B11 |
| hsa-miR-205-5p | HS3ST1 |
| hsa-miR-217 | HNF1B |
| hsa-miR-217 | HIVEP3 |
| hsa-miR-145-5p | HIC2 |
| hsa-miR-205-5p | HIATL1 |
| hsa-miR-217 | GRIK2 |
| hsa-miR-205-5p | GPM6A |
| hsa-miR-217 | GPM6A |
| hsa-miR-145-5p | GLIS1 |
| hsa-miR-145-5p | GGT7 |
| hsa-miR-145-5p | GABARAPL2 |
| hsa-miR-145-5p | FZD7 |
| hsa-miR-217 | FRMD5 |
| hsa-miR-205-5p | FRK |
| hsa-miR-145-5p | FOXO1 |
| hsa-miR-217 | FNDC3B |
| hsa-miR-145-5p | FNDC3A |
| hsa-miR-217 | FN1 |
| hsa-miR-145-5p | FLNB |
| hsa-miR-145-5p | FLI1 |
| hsa-miR-217 | FEM1C |
| hsa-miR-145-5p | FBXO28 |
| hsa-miR-205-5p | FBXO22 |
| hsa-miR-217 | FBXO11 |
| hsa-miR-217 | FBN2 |
| hsa-miR-217 | EZH2 |
| hsa-miR-145-5p | EYA3 |
| hsa-miR-205-5p | ESRRG |
| hsa-miR-217 | ESCO1 |
| hsa-miR-205-5p | ERRFI1 |
| hsa-miR-145-5p | ERLIN1 |
| hsa-miR-145-5p | ERG |
| hsa-miR-145-5p | EPB41L5 |
| hsa-miR-145-5p | ELMO1 |
| hsa-miR-145-5p | EIF4EBP2 |
| hsa-miR-217 | EIF4A2 |
| hsa-miR-217 | EHMT1 |
| hsa-miR-145-5p | DYRK1A |
| hsa-miR-145-5p | DUSP6 |
| hsa-miR-217 | DOCK3 |
| hsa-miR-205-5p | DMXL2 |
| hsa-miR-205-5p | DLG2 |
| hsa-miR-205-5p | DGCR8 |
| hsa-miR-205-5p | DDX5 |
| hsa-miR-217 | DACH1 |
| hsa-miR-145-5p | CTNNBIP1 |
| hsa-miR-145-5p | CSTF3 |
| hsa-miR-145-5p | CSMD3 |
| hsa-miR-205-5p | CPEB2 |
| hsa-miR-205-5p | CLTC |
| hsa-miR-145-5p | CLINT1 |
| hsa-miR-205-5p | CLDN11 |
| hsa-miR-145-5p | CITED2 |
| hsa-miR-217 | CHN2 |
| hsa-miR-205-5p | CHN1 |
| hsa-miR-145-5p | CDR2L |
| hsa-miR-205-5p | CDH11 |
| hsa-miR-145-5p | CDC37L1 |
| hsa-miR-145-5p | CCNL1 |
| hsa-miR-205-5p | CCNJ |
| hsa-miR-205-5p | CCDC93 |
| hsa-miR-145-5p | CCDC25 |
| hsa-miR-145-5p | CBFB |
| hsa-miR-205-5p | CASD1 |
| hsa-miR-205-5p | CANX |
| hsa-miR-205-5p | CALU |
| hsa-miR-205-5p | CALCRL |
| hsa-miR-145-5p | CACHD1 |
| hsa-miR-217 | C11ORF87 |
| hsa-miR-145-5p | C11ORF58 |
| hsa-miR-205-5p | BTBD3 |
| hsa-miR-217 | BAI3 |
| hsa-miR-145-5p | BACH2 |
| hsa-miR-145-5p | ATXN2 |
| hsa-miR-217 | ATP1B1 |
| hsa-miR-217 | ATP11C |
| hsa-miR-145-5p | ARPC5 |
| hsa-miR-145-5p | ARHGAP24 |
| hsa-miR-217 | APPBP2 |
| hsa-miR-145-5p | AP3S1 |
| hsa-miR-145-5p | AP1G1 |
| hsa-miR-217 | ANLN |
| hsa-miR-145-5p | ANGPT2 |
| hsa-miR-205-5p | AMOT |
| hsa-miR-145-5p | AKAP9 |
| hsa-miR-145-5p | AKAP12 |
| hsa-miR-145-5p | ADPGK |
| hsa-miR-145-5p | ADD3 |
| hsa-miR-205-5p | ADAMTS9 |
| hsa-miR-145-5p | ADAM19 |
| hsa-miR-145-5p | ACVR2A |
| hsa-miR-145-5p | ACVR1B |
| hsa-miR-145-5p | ACTG1 |
| hsa-miR-205-5p | ACTB |
| hsa-miR-145-5p | ACTB |
| hsa-miR-145-5p | ACSL4 |
| hsa-miR-205-5p | ACSL1 |
| hsa-miR-145-5p | ACBD3 |
| hsa-miR-145-5p | ABR |
| hsa-miR-205-5p | Sep4 |
