# Supplementary material for: Expression Profiles and Functional Analysis of Plasma Exosomal Circular RNAs in Acute Myocardial Infarction
Source: Biomed Res Int. 2022 Oct 1;2022:3458227. doi: 10.1155/2022/3458227 (PMC9547997; doi:10.1155/2022/3458227)
Supplement: Supplementary 12 — Supplementary Table S12: Cardiovascular disease-associated microRNAs which interacted with differentially expressed exosomal circRNAs and their potential targets in comparison of CAD and control. [file 3458227.f12.docx]

Supplementary Table S12 Cardiovascular disease-associated microRNAs which interacted with differentially expressed exosomal circRNAs and their potential targets in comparison of CAD and control.

| miRNAs | Target Gene symbols |
| --- | --- |
| hsa-miR-205-5p | BTBD3 |
| hsa-miR-205-5p | SBF2 |
| hsa-miR-205-5p | LAMC1 |
| hsa-miR-205-5p | MMD |
| hsa-miR-205-5p | KAT2B |
| hsa-miR-205-5p | SQLE |
| hsa-miR-205-5p | CCNJ |
| hsa-miR-205-5p | CLTC |
| hsa-miR-205-5p | GPM6A |
| hsa-miR-205-5p | PDE3B |
| hsa-miR-205-5p | RAB11FIP1 |
| hsa-miR-205-5p | ADAMTS9 |
| hsa-miR-205-5p | CASD1 |
| hsa-miR-205-5p | MED1 |
| hsa-miR-205-5p | WDR48 |
| hsa-miR-205-5p | VASN |
| hsa-miR-205-5p | AMOT |
| hsa-miR-205-5p | PRKCE |
| hsa-miR-205-5p | FBXO22 |
| hsa-miR-205-5p | MSL2 |
| hsa-miR-205-5p | RBM47 |
| hsa-miR-205-5p | LIN9 |
| hsa-miR-205-5p | ACTB |
| hsa-miR-205-5p | TRAK2 |
| hsa-miR-205-5p | SEL1L |
| hsa-miR-205-5p | NDUFA4 |
| hsa-miR-205-5p | NECAP1 |
| hsa-miR-205-5p | RTN3 |
| hsa-miR-205-5p | NR3C2 |
| hsa-miR-205-5p | NFAT5 |
| hsa-miR-205-5p | HSD17B11 |
| hsa-miR-205-5p | PJA2 |
| hsa-miR-205-5p | ESRRG |
| hsa-miR-205-5p | SCMH1 |
| hsa-miR-205-5p | SGMS1 |
| hsa-miR-205-5p | PPP1R15B |
| hsa-miR-205-5p | SMG7 |
| hsa-miR-205-5p | MARCKS |
| hsa-miR-205-5p | SNX27 |
| hsa-miR-205-5p | LCOR |
| hsa-miR-205-5p | SORBS1 |
| hsa-miR-205-5p | LPAR1 |
| hsa-miR-205-5p | HIATL1 |
| hsa-miR-205-5p | CCDC93 |
| hsa-miR-205-5p | ERRFI1 |
| hsa-miR-205-5p | KLF12 |
| hsa-miR-205-5p | PLCB1 |
| hsa-miR-205-5p | SIAH1 |
| hsa-miR-205-5p | DGCR8 |
| hsa-miR-205-5p | QKI |
| hsa-miR-205-5p | CALCRL |
| hsa-miR-205-5p | ZNF800 |
| hsa-miR-205-5p | CDH11 |
| hsa-miR-205-5p | DLG2 |
| hsa-miR-205-5p | CHN1 |
| hsa-miR-205-5p | MGRN1 |
| hsa-miR-205-5p | SATB2 |
| hsa-miR-205-5p | 4-Sep |
| hsa-miR-205-5p | PHC2 |
| hsa-miR-205-5p | ACSL1 |
| hsa-miR-205-5p | TSC22D1 |
| hsa-miR-205-5p | CALU |
| hsa-miR-205-5p | CPEB2 |
| hsa-miR-205-5p | FRK |
| hsa-miR-217 | APPBP2 |
| hsa-miR-217 | SIRT1 |
| hsa-miR-217 | CHN2 |
| hsa-miR-217 | HNF1B |
| hsa-miR-217 | MAPK8IP1 |
| hsa-miR-217 | RBM39 |
| hsa-miR-217 | RIN2 |
| hsa-miR-217 | KRAS |
| hsa-miR-217 | SLC38A2 |
| hsa-miR-217 | FBN2 |
| hsa-miR-217 | ANLN |
| hsa-miR-217 | DOCK3 |
| hsa-miR-217 | ESCO1 |
| hsa-miR-217 | ZCCHC2 |
| hsa-miR-217 | FEM1C |
| hsa-miR-217 | ST18 |
| hsa-miR-217 | SPOPL |
| hsa-miR-217 | GPM6A |
| hsa-miR-217 | WAPAL |
| hsa-miR-217 | DACH1 |
| hsa-miR-217 | PPM1D |
| hsa-miR-217 | YWHAG |
| hsa-miR-217 | PDS5B |
| hsa-miR-217 | KCNH5 |
| hsa-miR-217 | EIF4A2 |
| hsa-miR-217 | MYEF2 |
| hsa-miR-217 | C11ORF87 |
| hsa-miR-217 | ATP11C |
| hsa-miR-217 | LIN9 |
| hsa-miR-217 | TACC1 |
| hsa-miR-217 | PSMF1 |
| hsa-miR-217 | FNDC3B |
| hsa-miR-217 | NR4A2 |
| hsa-miR-217 | RAP2C |
| hsa-miR-217 | YTHDC1 |
| hsa-miR-217 | FN1 |
| hsa-miR-217 | ATP1B1 |
| hsa-miR-217 | BAI3 |
| hsa-miR-217 | LCOR |
| hsa-miR-217 | STAG2 |
| hsa-miR-217 | HIVEP3 |
| hsa-miR-217 | SLC31A1 |
| hsa-miR-217 | TMTC4 |
| hsa-miR-217 | PCNA |
| hsa-miR-217 | UBL3 |
| hsa-miR-217 | MIER3 |
| hsa-miR-217 | RTF1 |
| hsa-miR-217 | STT3A |
| hsa-miR-217 | SENP7 |
| hsa-miR-217 | STX1A |
| hsa-miR-217 | FBXO11 |
| hsa-miR-217 | VSNL1 |
| hsa-miR-217 | GRIK2 |
| hsa-miR-217 | NIPBL |
| hsa-miR-217 | EHMT1 |
| hsa-miR-217 | NOVA1 |
| hsa-miR-217 | EZH2 |
| hsa-miR-217 | FRMD5 |
| hsa-miR-217 | TACC2 |
| hsa-miR-217 | TBC1D15 |
| hsa-miR-205-5p | HS3ST1 |
| hsa-miR-205-5p | CLDN11 |
| hsa-miR-30a-3p | RALA |
| hsa-miR-30a-3p | LCP2 |
| hsa-miR-30a-3p | OSBPL6 |
| hsa-miR-30a-3p | ELK3 |
| hsa-miR-30a-3p | EGR1 |
| hsa-miR-30a-3p | CANX |
| hsa-miR-30a-3p | MEOX2 |
| hsa-miR-30a-3p | CREBBP |
| hsa-miR-30a-3p | EP300 |
| hsa-miR-30a-3p | EPAS1 |
| hsa-miR-30a-3p | GALNT7 |
| hsa-miR-30a-3p | SEMA3C |
| hsa-miR-30a-3p | CNPY2 |
| hsa-miR-30a-3p | TMEM45B |
| hsa-miR-30a-3p | RBM45 |
| hsa-miR-30a-3p | LRRTM1 |
| hsa-miR-30a-3p | C7ORF60 |
| hsa-miR-30a-3p | ZNF22 |
| hsa-miR-30a-3p | AP1G1 |
| hsa-miR-30a-3p | SLC25A33 |
| hsa-miR-30a-3p | MECP2 |
| hsa-miR-30a-3p | PITX2 |
| hsa-miR-30a-3p | SP3 |
| hsa-miR-30a-3p | C5ORF30 |
| hsa-miR-30a-3p | DBX2 |
| hsa-miR-30a-3p | DLST |
| hsa-miR-30a-3p | PPP2R5E |
| hsa-miR-30a-3p | ISCU |
| hsa-miR-30a-3p | USP1 |
| hsa-miR-30a-3p | HIRA |
| hsa-miR-30a-3p | MEF2C |
| hsa-miR-30a-3p | CAPRIN1 |
| hsa-miR-30a-3p | ERBB4 |
| hsa-miR-30a-3p | RGS7 |
| hsa-miR-30a-3p | AKAP9 |
| hsa-miR-30a-3p | SH3GLB1 |
| hsa-miR-30a-3p | FOXJ3 |
| hsa-miR-30a-3p | ZDHHC15 |
| hsa-miR-30a-3p | POU4F1 |
| hsa-miR-30a-3p | NRCAM |
| hsa-miR-30a-3p | YPEL5 |
| hsa-miR-30a-3p | SIAH1 |
| hsa-miR-30a-3p | NHS |
| hsa-miR-30a-3p | PSIP1 |
| hsa-miR-30a-3p | FAM49A |
| hsa-miR-30a-3p | CDC37L1 |
| hsa-miR-30a-3p | RYR3 |
| hsa-miR-30a-3p | TNPO3 |
| hsa-miR-30a-3p | ACYP2 |
| hsa-miR-30a-3p | PAIP2 |
| hsa-miR-30a-3p | UBE2G1 |
| hsa-miR-30a-3p | HMGA2 |
| hsa-miR-30a-3p | CAV1 |
| hsa-miR-30a-3p | SS18 |
| hsa-miR-30a-3p | SH3GL3 |
| hsa-miR-30a-3p | ARID4A |
| hsa-miR-30a-3p | UBE2J1 |
| hsa-miR-30a-3p | RARB |
| hsa-miR-30a-3p | KIAA1324L |
| hsa-miR-30a-3p | MEF2D |
| hsa-miR-30a-3p | EIF1 |
| hsa-miR-30a-3p | ZEB2 |
| hsa-miR-30a-3p | MED12L |
| hsa-miR-30a-3p | SLITRK3 |
| hsa-miR-30a-3p | FNDC5 |
| hsa-miR-30a-3p | NPY2R |
| hsa-miR-30a-3p | RUNX1T1 |
| hsa-miR-30a-3p | FAM104A |
| hsa-miR-221-3p | CD4 |
| hsa-miR-221-3p | DMRT3 |
| hsa-miR-221-3p | NRK |
| hsa-miR-221-3p | EIF3J |
| hsa-miR-221-3p | CTCF |
| hsa-miR-221-3p | TIMP3 |
| hsa-miR-221-3p | TCF12 |
| hsa-miR-221-3p | PAK1 |
| hsa-miR-221-3p | FOS |
| hsa-miR-221-3p | MAT2A |
| hsa-miR-221-3p | PCDHA10 |
| hsa-miR-221-3p | AGFG1 |
| hsa-miR-221-3p | SNCB |
| hsa-miR-221-3p | FOXN2 |
| hsa-miR-221-3p | MYLIP |
| hsa-miR-221-3p | KIF16B |
| hsa-miR-221-3p | TOX |
| hsa-miR-221-3p | HIPK1 |
| hsa-miR-221-3p | INA |
| hsa-miR-221-3p | GNAI3 |
| hsa-miR-221-3p | HECTD2 |
| hsa-miR-221-3p | PPP6C |
| hsa-miR-221-3p | LPPR1 |
| hsa-miR-221-3p | RBM24 |
| hsa-miR-221-3p | MIER3 |
| hsa-miR-221-3p | RFX3 |
| hsa-miR-221-3p | IGF2BP2 |
| hsa-miR-221-3p | POGZ |
| hsa-miR-221-3p | IRF2 |
| hsa-miR-221-3p | MAPK10 |
| hsa-miR-221-3p | FERMT2 |
| hsa-miR-221-3p | TRPS1 |
| hsa-miR-221-3p | PCDHA12 |
| hsa-miR-221-3p | PCDHA11 |
| hsa-miR-221-3p | NLK |
| hsa-miR-221-3p | ZFPM2 |
| hsa-miR-221-3p | TMCC1 |
| hsa-miR-221-3p | ZEB2 |
| hsa-miR-221-3p | VAPB |
| hsa-miR-221-3p | FNDC3A |
| hsa-miR-221-3p | PCDHA1 |
| hsa-miR-221-3p | MYO10 |
| hsa-miR-221-3p | PCDHA3 |
| hsa-miR-221-3p | PCDHA7 |
| hsa-miR-221-3p | PCDHA2 |
| hsa-miR-221-3p | PCDHA6 |
| hsa-miR-221-3p | PCDHA5 |
| hsa-miR-221-3p | PCDHA9 |
| hsa-miR-221-3p | ZNF385A |
| hsa-miR-145-5p | REEP1 |
| hsa-miR-145-5p | NUFIP2 |
| hsa-miR-145-5p | C11ORF58 |
| hsa-miR-145-5p | LOX |
| hsa-miR-145-5p | TBPL1 |
| hsa-miR-145-5p | NAA50 |
| hsa-miR-145-5p | ACVR2A |
| hsa-miR-145-5p | RIN2 |
| hsa-miR-145-5p | SBF2 |
| hsa-miR-145-5p | ADAM19 |
| hsa-miR-145-5p | BACH2 |
| hsa-miR-145-5p | ACVR1B |
| hsa-miR-145-5p | NUAK1 |
| hsa-miR-145-5p | MPP5 |
| hsa-miR-145-5p | XRN1 |
| hsa-miR-145-5p | SEMA3A |
| hsa-miR-145-5p | ADD3 |
| hsa-miR-145-5p | MPZL2 |
| hsa-miR-145-5p | DUSP6 |
| hsa-miR-145-5p | PDCD4 |
| hsa-miR-145-5p | UXS1 |
| hsa-miR-145-5p | FZD7 |
| hsa-miR-145-5p | CBFB |
| hsa-miR-145-5p | CCNL1 |
| hsa-miR-145-5p | KIAA0355 |
| hsa-miR-145-5p | AP1G1 |
| hsa-miR-145-5p | ABR |
| hsa-miR-145-5p | SCAMP3 |
| hsa-miR-145-5p | VASN |
| hsa-miR-145-5p | IRS1 |
| hsa-miR-145-5p | RTKN |
| hsa-miR-145-5p | SPSB4 |
| hsa-miR-145-5p | ADPGK |
| hsa-miR-145-5p | GLIS1 |
| hsa-miR-145-5p | AP3S1 |
| hsa-miR-145-5p | MKL2 |
| hsa-miR-145-5p | RAPH1 |
| hsa-miR-145-5p | UBA6 |
| hsa-miR-145-5p | CSTF3 |
| hsa-miR-145-5p | ANGPT2 |
| hsa-miR-145-5p | LENG8 |
| hsa-miR-145-5p | PSD3 |
| hsa-miR-145-5p | SMAD3 |
| hsa-miR-145-5p | LRRC16A |
| hsa-miR-145-5p | ACTB |
| hsa-miR-145-5p | ACTG1 |
| hsa-miR-145-5p | GGT7 |
| hsa-miR-145-5p | CDR2L |
| hsa-miR-145-5p | TRIM2 |
| hsa-miR-145-5p | DYRK1A |
| hsa-miR-145-5p | ELMO1 |
| hsa-miR-145-5p | SEMA6A |
| hsa-miR-145-5p | CSMD3 |
| hsa-miR-145-5p | YTHDC1 |
| hsa-miR-145-5p | RGS7 |
| hsa-miR-145-5p | NET1 |
| hsa-miR-145-5p | SRGAP1 |
| hsa-miR-145-5p | AKAP9 |
| hsa-miR-145-5p | CCDC25 |
| hsa-miR-145-5p | ZDHHC9 |
| hsa-miR-145-5p | FLNB |
| hsa-miR-145-5p | ARPC5 |
| hsa-miR-145-5p | MAP3K3 |
| hsa-miR-145-5p | INO80 |
| hsa-miR-145-5p | KIF21A |
| hsa-miR-145-5p | ACBD3 |
| hsa-miR-145-5p | IVNS1ABP |
| hsa-miR-145-5p | CITED2 |
| hsa-miR-145-5p | REV3L |
| hsa-miR-145-5p | SNX27 |
| hsa-miR-145-5p | PTGFR |
| hsa-miR-145-5p | CACHD1 |
| hsa-miR-145-5p | PLCE1 |
| hsa-miR-145-5p | ZFYVE9 |
| hsa-miR-145-5p | RSPO1 |
| hsa-miR-145-5p | SLC25A25 |
| hsa-miR-145-5p | EIF4EBP2 |
| hsa-miR-145-5p | YTHDF2 |
| hsa-miR-145-5p | RAB14 |
| hsa-miR-145-5p | EYA3 |
| hsa-miR-145-5p | CTNNBIP1 |
| hsa-miR-145-5p | NEDD9 |
| hsa-miR-145-5p | FOXO1 |
| hsa-miR-145-5p | SLITRK4 |
| hsa-miR-145-5p | CDC37L1 |
| hsa-miR-145-5p | SACM1L |
| hsa-miR-145-5p | TIRAP |
| hsa-miR-145-5p | MDFIC |
| hsa-miR-145-5p | ARHGAP24 |
| hsa-miR-145-5p | TM9SF4 |
| hsa-miR-145-5p | ERG |
| hsa-miR-145-5p | MYO5A |
| hsa-miR-145-5p | SLITRK6 |
| hsa-miR-145-5p | AKAP12 |
| hsa-miR-145-5p | HIC2 |
| hsa-miR-145-5p | MAP4K4 |
| hsa-miR-145-5p | PLCL2 |
| hsa-miR-145-5p | ERLIN1 |
| hsa-miR-145-5p | FBXO28 |
| hsa-miR-145-5p | RNF216 |
| hsa-miR-145-5p | PAN2 |
| hsa-miR-145-5p | ZBTB10 |
| hsa-miR-145-5p | USP46 |
| hsa-miR-145-5p | EPB41L5 |
| hsa-miR-145-5p | RASA1 |
| hsa-miR-145-5p | PXN |
| hsa-miR-145-5p | ACSL4 |
| hsa-miR-145-5p | FNDC3A |
| hsa-miR-145-5p | PPP3CA |
| hsa-miR-145-5p | CLINT1 |
| hsa-miR-145-5p | FLI1 |
| hsa-miR-145-5p | KDM2B |
| hsa-miR-145-5p | ATXN2 |
| hsa-miR-145-5p | SPATS2 |
| hsa-miR-145-5p | TLN2 |
| hsa-miR-145-5p | ZNF423 |
| hsa-miR-145-5p | GABARAPL2 |
| hsa-miR-23b-3p | NUFIP2 |
| hsa-miR-23b-3p | WBP2 |
| hsa-miR-23b-3p | ATP6V1B2 |
| hsa-miR-23b-3p | CFDP1 |
| hsa-miR-23b-3p | ARF6 |
| hsa-miR-23b-3p | UBE2O |
| hsa-miR-23b-3p | TGIF1 |
| hsa-miR-23b-3p | RFX6 |
| hsa-miR-23b-3p | PUM2 |
| hsa-miR-23b-3p | IL6R |
| hsa-miR-23b-3p | TOX |
| hsa-miR-23b-3p | COL4A5 |
| hsa-miR-23b-3p | NEK6 |
| hsa-miR-23b-3p | UBE2D1 |
| hsa-miR-23b-3p | AUH |
| hsa-miR-23b-3p | KDM6A |
| hsa-miR-23b-3p | ANO4 |
| hsa-miR-23b-3p | ZNF423 |
| hsa-miR-34a-5p | FOXJ2 |
| hsa-miR-34a-5p | TMEM109 |
| hsa-miR-34a-5p | SEC61A1 |
| hsa-miR-34a-5p | JAG1 |
| hsa-miR-34a-5p | DAGLA |
| hsa-miR-34a-5p | CNTNAP1 |
| hsa-miR-34a-5p | LMAN2L |
| hsa-miR-34a-5p | EVI5L |
| hsa-miR-34a-5p | UHRF2 |
| hsa-miR-34a-5p | STC1 |
| hsa-miR-34a-5p | CELF3 |
| hsa-miR-34a-5p | ZER1 |
| hsa-miR-34a-5p | SYVN1 |
| hsa-miR-34a-5p | SVOP |
| hsa-miR-34a-5p | CTNND2 |
| hsa-miR-34a-5p | VAMP2 |
| hsa-miR-34a-5p | FOXP1 |
| hsa-miR-34a-5p | AHCYL2 |
| hsa-miR-34a-5p | PTPRM |
| hsa-miR-34a-5p | SLC44A2 |
| hsa-miR-34a-5p | DPYSL4 |
| hsa-miR-34a-5p | CRTC1 |
| hsa-miR-34a-5p | ZCCHC17 |
| hsa-miR-34a-5p | ARID4B |
| hsa-miR-34a-5p | HK1 |
| hsa-miR-34a-5p | C1ORF116 |
| hsa-miR-34a-5p | TMEM184B |
| hsa-miR-34a-5p | VCL |
| hsa-miR-34a-5p | MPP2 |
| hsa-miR-34a-5p | WASF1 |
| hsa-miR-34a-5p | GRK6 |
| hsa-miR-34a-5p | DAAM1 |
| hsa-miR-34a-5p | PKIA |
| hsa-miR-34a-5p | 3-Sep |
| hsa-miR-34a-5p | MET |
| hsa-miR-34a-5p | B4GALT2 |
| hsa-miR-34a-5p | ACSL1 |
| hsa-miR-34a-5p | ACTR1A |
| hsa-miR-34a-5p | LRRC55 |
| hsa-miR-34a-5p | RPS6KA4 |
| hsa-miR-34a-5p | GLCE |
| hsa-miR-34a-5p | MEX3C |
| hsa-miR-27a-3p | APPBP2 |
| hsa-miR-27a-3p | C17ORF85 |
| hsa-miR-27a-3p | BCL3 |
| hsa-miR-27a-3p | NGFR |
| hsa-miR-27a-3p | KIAA1199 |
| hsa-miR-27a-3p | SFRP1 |
| hsa-miR-27a-3p | HOXA5 |
| hsa-miR-27a-3p | PPIF |
| hsa-miR-27a-3p | MAPK14 |
| hsa-miR-27a-3p | ISL1 |
| hsa-miR-27a-3p | HBEGF |
| hsa-miR-27a-3p | ID2 |
| hsa-miR-27a-3p | ELL2 |
| hsa-miR-27a-3p | HOXB8 |
| hsa-miR-27a-3p | HOXC6 |
| hsa-miR-27a-3p | NRK |
| hsa-miR-27a-3p | LYPD3 |
| hsa-miR-27a-3p | SGPP1 |
| hsa-miR-27a-3p | DLL4 |
| hsa-miR-27a-3p | ATXN10 |
| hsa-miR-27a-3p | GFPT2 |
| hsa-miR-27a-3p | SNAP25 |
| hsa-miR-27a-3p | SLC39A11 |
| hsa-miR-27a-3p | PDGFRA |
| hsa-miR-27a-3p | ADAM19 |
| hsa-miR-27a-3p | CAB39 |
| hsa-miR-27a-3p | SLCO5A1 |
| hsa-miR-27a-3p | DNAJC13 |
| hsa-miR-27a-3p | KPNA3 |
| hsa-miR-27a-3p | NEO1 |
| hsa-miR-27a-3p | MMD |
| hsa-miR-27a-3p | ARFGEF1 |
| hsa-miR-27a-3p | WSB1 |
| hsa-miR-27a-3p | ACVR1 |
| hsa-miR-27a-3p | DNAJC27 |
| hsa-miR-27a-3p | GALNT7 |
| hsa-miR-27a-3p | NPTX2 |
| hsa-miR-27a-3p | CCNJ |
| hsa-miR-27a-3p | MESDC1 |
| hsa-miR-27a-3p | UBE2F |
| hsa-miR-27a-3p | PLK2 |
| hsa-miR-27a-3p | HAPLN1 |
| hsa-miR-27a-3p | CYP39A1 |
| hsa-miR-27a-3p | EGFR |
| hsa-miR-27a-3p | NRBF2 |
| hsa-miR-27a-3p | SLC7A11 |
| hsa-miR-27a-3p | ANK3 |
| hsa-miR-27a-3p | FBXW7 |
| hsa-miR-27a-3p | DTNA |
| hsa-miR-27a-3p | HOXA10 |
| hsa-miR-27a-3p | FAM105B |
| hsa-miR-27a-3p | SH3RF1 |
| hsa-miR-27a-3p | DKK2 |
| hsa-miR-27a-3p | USP25 |
| hsa-miR-27a-3p | FZD7 |
| hsa-miR-27a-3p | RAB11FIP1 |
| hsa-miR-27a-3p | UNC5D |
| hsa-miR-27a-3p | SLC6A1 |
| hsa-miR-27a-3p | CBFB |
| hsa-miR-27a-3p | NR2F6 |
| hsa-miR-27a-3p | ITGA5 |
| hsa-miR-27a-3p | KBTBD8 |
| hsa-miR-27a-3p | CDS1 |
| hsa-miR-27a-3p | PRICKLE2 |
| hsa-miR-27a-3p | NDUFS4 |
| hsa-miR-27a-3p | NEUROD6 |
| hsa-miR-27a-3p | EN2 |
| hsa-miR-27a-3p | ORC5 |
| hsa-miR-27a-3p | TMUB1 |
| hsa-miR-27a-3p | FBXO33 |
| hsa-miR-27a-3p | WEE1 |
| hsa-miR-27a-3p | PTGER4 |
| hsa-miR-27a-3p | RNF139 |
| hsa-miR-27a-3p | ADORA2B |
| hsa-miR-27a-3p | IRS1 |
| hsa-miR-27a-3p | INSM2 |
| hsa-miR-27a-3p | PDE7B |
| hsa-miR-27a-3p | TMEM9B |
| hsa-miR-27a-3p | FAM134C |
| hsa-miR-27a-3p | MEPCE |
| hsa-miR-27a-3p | ARHGAP12 |
| hsa-miR-27a-3p | NR1D2 |
| hsa-miR-27a-3p | ING5 |
| hsa-miR-27a-3p | AQP11 |
| hsa-miR-27a-3p | PDS5B |
| hsa-miR-27a-3p | NEURL4 |
| hsa-miR-27a-3p | C8ORF4 |
| hsa-miR-27a-3p | PDIA5 |
| hsa-miR-27a-3p | PLAG1 |
| hsa-miR-27a-3p | EDEM3 |
| hsa-miR-27a-3p | GLTP |
| hsa-miR-27a-3p | B4GALT3 |
| hsa-miR-27a-3p | RNF144A |
| hsa-miR-27a-3p | TMTC2 |
| hsa-miR-27a-3p | CCDC71 |
| hsa-miR-27a-3p | EHD3 |
| hsa-miR-27a-3p | PANK1 |
| hsa-miR-27a-3p | E2F7 |
| hsa-miR-27a-3p | MED14 |
| hsa-miR-27a-3p | NETO1 |
| hsa-miR-27a-3p | CBFA2T3 |
| hsa-miR-27a-3p | ATP11C |
| hsa-miR-27a-3p | PAPPA |
| hsa-miR-27a-3p | POU3F2 |
| hsa-miR-27a-3p | MYT1 |
| hsa-miR-27a-3p | CSF1 |
| hsa-miR-27a-3p | RCAN2 |
| hsa-miR-27a-3p | INPP5J |
| hsa-miR-27a-3p | SS18L1 |
| hsa-miR-27a-3p | KIAA1033 |
| hsa-miR-27a-3p | APAF1 |
| hsa-miR-27a-3p | DCUN1D4 |
| hsa-miR-27a-3p | PPP1CC |
| hsa-miR-27a-3p | HORMAD2 |
| hsa-miR-27a-3p | C10ORF137 |
| hsa-miR-27a-3p | FAM184A |
| hsa-miR-27a-3p | NEDD4 |
| hsa-miR-27a-3p | OTX2 |
| hsa-miR-27a-3p | EDNRA |
| hsa-miR-27a-3p | PAQR9 |
| hsa-miR-27a-3p | GATA2 |
| hsa-miR-27a-3p | CDH5 |
| hsa-miR-27a-3p | NRXN1 |
| hsa-miR-27a-3p | SEMA6A |
| hsa-miR-27a-3p | MBNL2 |
| hsa-miR-27a-3p | GPAM |
| hsa-miR-27a-3p | YWHAB |
| hsa-miR-27a-3p | TMEM110 |
| hsa-miR-27a-3p | STX16 |
| hsa-miR-27a-3p | SEMA6D |
| hsa-miR-27a-3p | STK39 |
| hsa-miR-27a-3p | DCX |
| hsa-miR-27a-3p | HNRNPF |
| hsa-miR-27a-3p | ANK2 |
| hsa-miR-27a-3p | ADCY6 |
| hsa-miR-27a-3p | CDK18 |
| hsa-miR-27a-3p | CDC42BPB |
| hsa-miR-27a-3p | CNOT7 |
| hsa-miR-27a-3p | NGFRAP1 |
| hsa-miR-27a-3p | SGMS1 |
| hsa-miR-27a-3p | ITSN2 |
| hsa-miR-27a-3p | E2F6 |
| hsa-miR-27a-3p | VIP |
| hsa-miR-27a-3p | NR5A2 |
| hsa-miR-27a-3p | RGS1 |
| hsa-miR-27a-3p | EYA4 |
| hsa-miR-27a-3p | MARCKS |
| hsa-miR-27a-3p | EBF3 |
| hsa-miR-27a-3p | RNGTT |
| hsa-miR-27a-3p | BAG2 |
| hsa-miR-27a-3p | ZZZ3 |
| hsa-miR-27a-3p | GNG12 |
| hsa-miR-27a-3p | ROR1 |
| hsa-miR-27a-3p | LCOR |
| hsa-miR-27a-3p | NXT2 |
| hsa-miR-27a-3p | FAM78A |
| hsa-miR-27a-3p | ZCCHC24 |
| hsa-miR-27a-3p | FRS3 |
| hsa-miR-27a-3p | FOXP4 |
| hsa-miR-27a-3p | NEK6 |
| hsa-miR-27a-3p | NKAIN1 |
| hsa-miR-27a-3p | ABCA1 |
| hsa-miR-27a-3p | RND3 |
| hsa-miR-27a-3p | SLITRK1 |
| hsa-miR-27a-3p | SPRY2 |
| hsa-miR-27a-3p | FLRT3 |
| hsa-miR-27a-3p | LPAR6 |
| hsa-miR-27a-3p | FOXO1 |
| hsa-miR-27a-3p | IRF4 |
| hsa-miR-27a-3p | MIER3 |
| hsa-miR-205-5p | DDX5 |
| hsa-miR-27a-3p | STAG1 |
| hsa-miR-27a-3p | GOLM1 |
| hsa-miR-27a-3p | EYA1 |
| hsa-miR-27a-3p | UNKL |
| hsa-miR-27a-3p | CCNK |
| hsa-miR-27a-3p | OPA1 |
| hsa-miR-27a-3p | NCOA7 |
| hsa-miR-27a-3p | GRB2 |
| hsa-miR-27a-3p | GALNT3 |
| hsa-miR-27a-3p | PLXND1 |
| hsa-miR-27a-3p | ZNF800 |
| hsa-miR-27a-3p | CDH11 |
| hsa-miR-27a-3p | PHF15 |
| hsa-miR-27a-3p | LBH |
| hsa-miR-27a-3p | ZHX1 |
| hsa-miR-27a-3p | PSMA1 |
| hsa-miR-27a-3p | PKIA |
| hsa-miR-27a-3p | CACNB2 |
| hsa-miR-27a-3p | NFE2L2 |
| hsa-miR-27a-3p | MEIS2 |
| hsa-miR-27a-3p | SOCS6 |
| hsa-miR-27a-3p | DOT1L |
| hsa-miR-27a-3p | JMJD1C |
| hsa-miR-27a-3p | NRIP1 |
| hsa-miR-27a-3p | TAPT1 |
| hsa-miR-27a-3p | MATN3 |
| hsa-miR-27a-3p | FOXP2 |
| hsa-miR-27a-3p | NABP1 |
| hsa-miR-27a-3p | CPEB3 |
| hsa-miR-27a-3p | LITAF |
| hsa-miR-27a-3p | SATB2 |
| hsa-miR-27a-3p | PLCL2 |
| hsa-miR-27a-3p | LIMK1 |
| hsa-miR-27a-3p | GNS |
| hsa-miR-27a-3p | ZDHHC17 |
| hsa-miR-27a-3p | RELN |
| hsa-miR-27a-3p | TMCC1 |
| hsa-miR-27a-3p | TROVE2 |
| hsa-miR-27a-3p | CREB1 |
| hsa-miR-27a-3p | AKIRIN1 |
| hsa-miR-27a-3p | KCNA6 |
| hsa-miR-27a-3p | GSPT1 |
| hsa-miR-27a-3p | USP46 |
| hsa-miR-27a-3p | PDHX |
| hsa-miR-27a-3p | REPS1 |
| hsa-miR-27a-3p | CADM1 |
| hsa-miR-27a-3p | NOVA1 |
| hsa-miR-27a-3p | ARMC8 |
| hsa-miR-27a-3p | C1ORF52 |
| hsa-miR-27a-3p | CACNA2D3 |
| hsa-miR-27a-3p | RYBP |
| hsa-miR-27a-3p | TMED5 |
| hsa-miR-27a-3p | RGL2 |
| hsa-miR-27a-3p | CLCN3 |
| hsa-miR-27a-3p | FAM193B |
| hsa-miR-27a-3p | UBE2W |
| hsa-miR-27a-3p | ZFHX4 |
| hsa-miR-27a-3p | NXF1 |
| hsa-miR-27a-3p | C11ORF57 |
| hsa-miR-27a-3p | BCORL1 |
| hsa-miR-27a-3p | CCM2 |
| hsa-miR-27a-3p | TMEM189 |
| hsa-miR-27a-3p | CA10 |
| hsa-miR-27a-3p | GOSR2 |
| hsa-miR-27a-3p | TLK2 |
| hsa-miR-27a-3p | LSM12 |
| hsa-miR-27a-3p | FOSB |
| hsa-miR-27a-3p | BRSK1 |
| hsa-miR-27a-3p | H3F3B |
| hsa-miR-210-3p | GPD1L |
| hsa-miR-210-3p | MDGA1 |
| hsa-miR-210-3p | KCMF1 |
| hsa-miR-205-5p | LRP1 |
| hsa-miR-205-5p | CANX |
| hsa-miR-205-5p | DMXL2 |
