# Supplementary material for: Expression Profiles and Functional Analysis of Plasma Exosomal Circular RNAs in Acute Myocardial Infarction
Source: Biomed Res Int. 2022 Oct 1;2022:3458227. doi: 10.1155/2022/3458227 (PMC9547997; doi:10.1155/2022/3458227)
Supplement: Supplementary 13 — Supplementary Table S13: Cardiovascular disease-associated microRNAs which interacted with differentially expressed exosomal circRNAs and their potential targets in comparison of AMI and CAD. [file 3458227.f13.docx]

Supplementary Table S13 Cardiovascular disease-associated microRNAs which interacted with differentially expressed exosomal circRNAs and their potential targets in comparison of AMI and CAD.

| miRNAs | Target Gene symbols | |
| --- | --- | --- |
| hsa-miR-221-3p | CD4 |  |
| hsa-miR-221-3p | DMRT3 |  |
| hsa-miR-221-3p | NRK |  |
| hsa-miR-221-3p | EIF3J |  |
| hsa-miR-221-3p | CTCF |  |
| hsa-miR-221-3p | TIMP3 |  |
| hsa-miR-221-3p | TCF12 |  |
| hsa-miR-221-3p | PAK1 |  |
| hsa-miR-221-3p | FOS |  |
| hsa-miR-221-3p | MAT2A |  |
| hsa-miR-221-3p | PCDHA10 |  |
| hsa-miR-221-3p | AGFG1 |  |
| hsa-miR-221-3p | SNCB |  |
| hsa-miR-221-3p | FOXN2 |  |
| hsa-miR-221-3p | MYLIP |  |
| hsa-miR-221-3p | KIF16B |  |
| hsa-miR-221-3p | TOX |  |
| hsa-miR-221-3p | HIPK1 |  |
| hsa-miR-221-3p | INA |  |
| hsa-miR-221-3p | GNAI3 |  |
| hsa-miR-221-3p | HECTD2 |  |
| hsa-miR-221-3p | PPP6C |  |
| hsa-miR-221-3p | LPPR1 |  |
| hsa-miR-221-3p | RBM24 |  |
| hsa-miR-221-3p | MIER3 |  |
| hsa-miR-221-3p | RFX3 |  |
| hsa-miR-221-3p | IGF2BP2 |  |
| hsa-miR-221-3p | POGZ |  |
| hsa-miR-221-3p | IRF2 |  |
| hsa-miR-221-3p | MAPK10 |  |
| hsa-miR-221-3p | FERMT2 |  |
| hsa-miR-221-3p | TRPS1 |  |
| hsa-miR-221-3p | PCDHA12 |  |
| hsa-miR-221-3p | PCDHA11 |  |
| hsa-miR-221-3p | NLK |  |
| hsa-miR-221-3p | ZFPM2 |  |
| hsa-miR-221-3p | TMCC1 |  |
| hsa-miR-221-3p | ZEB2 |  |
| hsa-miR-221-3p | VAPB |  |
| hsa-miR-221-3p | FNDC3A |  |
| hsa-miR-221-3p | PCDHA1 |  |
| hsa-miR-221-3p | MYO10 |  |
| hsa-miR-221-3p | PCDHA3 |  |
| hsa-miR-221-3p | PCDHA7 |  |
| hsa-miR-221-3p | PCDHA2 |  |
| hsa-miR-221-3p | PCDHA6 |  |
| hsa-miR-221-3p | PCDHA5 |  |
| hsa-miR-221-3p | PCDHA9 |  |
| hsa-miR-221-3p | ZNF385A |  |
| hsa-miR-30a-3p | RALA |  |
| hsa-miR-30a-3p | LCP2 |  |
| hsa-miR-30a-3p | OSBPL6 |  |
| hsa-miR-30a-3p | ELK3 |  |
| hsa-miR-30a-3p | EGR1 |  |
| hsa-miR-30a-3p | CANX |  |
| hsa-miR-30a-3p | MEOX2 |  |
| hsa-miR-30a-3p | CREBBP |  |
| hsa-miR-30a-3p | EP300 |  |
| hsa-miR-30a-3p | EPAS1 |  |
| hsa-miR-30a-3p | GALNT7 |  |
| hsa-miR-30a-3p | SEMA3C |  |
| hsa-miR-30a-3p | CNPY2 |  |
| hsa-miR-30a-3p | TMEM45B |  |
| hsa-miR-30a-3p | RBM45 |  |
| hsa-miR-30a-3p | LRRTM1 |  |
| hsa-miR-30a-3p | C7ORF60 |  |
| hsa-miR-30a-3p | ZNF22 |  |
| hsa-miR-30a-3p | AP1G1 |  |
| hsa-miR-30a-3p | SLC25A33 |  |
| hsa-miR-30a-3p | MECP2 |  |
| hsa-miR-30a-3p | PITX2 |  |
| hsa-miR-30a-3p | SP3 |  |
| hsa-miR-30a-3p | C5ORF30 |  |
| hsa-miR-30a-3p | DBX2 |  |
| hsa-miR-30a-3p | DLST |  |
| hsa-miR-30a-3p | PPP2R5E |  |
| hsa-miR-30a-3p | ISCU |  |
| hsa-miR-30a-3p | USP1 |  |
| hsa-miR-30a-3p | HIRA |  |
| hsa-miR-30a-3p | MEF2C |  |
| hsa-miR-30a-3p | CAPRIN1 |  |
| hsa-miR-30a-3p | ERBB4 |  |
| hsa-miR-30a-3p | RGS7 |  |
| hsa-miR-30a-3p | AKAP9 |  |
| hsa-miR-30a-3p | SH3GLB1 |  |
| hsa-miR-30a-3p | FOXJ3 |  |
| hsa-miR-30a-3p | ZDHHC15 |  |
| hsa-miR-30a-3p | POU4F1 |  |
| hsa-miR-30a-3p | NRCAM |  |
| hsa-miR-30a-3p | YPEL5 |  |
| hsa-miR-30a-3p | SIAH1 |  |
| hsa-miR-30a-3p | NHS |  |
| hsa-miR-30a-3p | PSIP1 |  |
| hsa-miR-30a-3p | FAM49A |  |
| hsa-miR-30a-3p | CDC37L1 |  |
| hsa-miR-30a-3p | RYR3 |  |
| hsa-miR-30a-3p | TNPO3 |  |
| hsa-miR-30a-3p | ACYP2 |  |
| hsa-miR-30a-3p | PAIP2 |  |
| hsa-miR-30a-3p | UBE2G1 |  |
| hsa-miR-30a-3p | HMGA2 |  |
| hsa-miR-30a-3p | CAV1 |  |
| hsa-miR-30a-3p | SS18 |  |
| hsa-miR-30a-3p | SH3GL3 |  |
| hsa-miR-30a-3p | ARID4A |  |
| hsa-miR-30a-3p | UBE2J1 |  |
| hsa-miR-30a-3p | RARB |  |
| hsa-miR-30a-3p | KIAA1324L |  |
| hsa-miR-30a-3p | MEF2D |  |
| hsa-miR-30a-3p | EIF1 |  |
| hsa-miR-30a-3p | ZEB2 |  |
| hsa-miR-30a-3p | MED12L |  |
| hsa-miR-30a-3p | SLITRK3 |  |
| hsa-miR-30a-3p | FNDC5 |  |
| hsa-miR-30a-3p | NPY2R |  |
| hsa-miR-30a-3p | RUNX1T1 |  |
| hsa-miR-30a-3p | FAM104A |  |
| hsa-miR-34a-5p | FOXJ2 |  |
| hsa-miR-34a-5p | TMEM109 |  |
| hsa-miR-34a-5p | SEC61A1 |  |
| hsa-miR-34a-5p | JAG1 |  |
| hsa-miR-34a-5p | DAGLA |  |
| hsa-miR-34a-5p | CNTNAP1 |  |
| hsa-miR-34a-5p | LMAN2L |  |
| hsa-miR-34a-5p | EVI5L |  |
| hsa-miR-34a-5p | UHRF2 |  |
| hsa-miR-34a-5p | STC1 |  |
| hsa-miR-34a-5p | CELF3 |  |
| hsa-miR-34a-5p | ZER1 |  |
| hsa-miR-34a-5p | SYVN1 |  |
| hsa-miR-34a-5p | SVOP |  |
| hsa-miR-34a-5p | CTNND2 |  |
| hsa-miR-34a-5p | VAMP2 |  |
| hsa-miR-34a-5p | FOXP1 |  |
| hsa-miR-34a-5p | AHCYL2 |  |
| hsa-miR-34a-5p | PTPRM |  |
| hsa-miR-34a-5p | SLC44A2 |  |
| hsa-miR-34a-5p | DPYSL4 |  |
| hsa-miR-34a-5p | CRTC1 |  |
| hsa-miR-34a-5p | ZCCHC17 |  |
| hsa-miR-34a-5p | ARID4B |  |
| hsa-miR-34a-5p | HK1 |  |
| hsa-miR-34a-5p | C1ORF116 |  |
| hsa-miR-34a-5p | TMEM184B |  |
| hsa-miR-34a-5p | VCL |  |
| hsa-miR-34a-5p | MPP2 |  |
| hsa-miR-34a-5p | WASF1 |  |
| hsa-miR-34a-5p | GRK6 |  |
| hsa-miR-34a-5p | DAAM1 |  |
| hsa-miR-34a-5p | PKIA |  |
| hsa-miR-34a-5p | sep3 |  |
| hsa-miR-34a-5p | MET |  |
| hsa-miR-34a-5p | B4GALT2 |  |
| hsa-miR-34a-5p | ACSL1 |  |
| hsa-miR-34a-5p | ACTR1A |  |
| hsa-miR-34a-5p | LRRC55 |  |
| hsa-miR-34a-5p | RPS6KA4 |  |
| hsa-miR-34a-5p | GLCE |  |
| hsa-miR-34a-5p | MEX3C |  |
| hsa-miR-320a | CDK13 |  |
| hsa-miR-320a | ELL2 |  |
| hsa-miR-320a | MAPK8IP3 |  |
| hsa-miR-320a | CAB39 |  |
| hsa-miR-320a | PLXNC1 |  |
| hsa-miR-320a | IGF2BP3 |  |
| hsa-miR-320a | ANKRD13A |  |
| hsa-miR-320a | N4BP1 |  |
| hsa-miR-320a | CDH20 |  |
| hsa-miR-320a | CDH2 |  |
| hsa-miR-320a | SPOPL |  |
| hsa-miR-320a | PAN3 |  |
| hsa-miR-320a | DTNA |  |
| hsa-miR-320a | MSI2 |  |
| hsa-miR-320a | TOMM70A |  |
| hsa-miR-320a | USP25 |  |
| hsa-miR-320a | RBM45 |  |
| hsa-miR-320a | MMP16 |  |
| hsa-miR-320a | ZIC3 |  |
| hsa-miR-320a | FOXQ1 |  |
| hsa-miR-320a | TSC1 |  |
| hsa-miR-320a | KCNS3 |  |
| hsa-miR-320a | SUV420H1 |  |
| hsa-miR-320a | FAM84B |  |
| hsa-miR-320a | MUC13 |  |
| hsa-miR-320a | ING5 |  |
| hsa-miR-320a | ZMYM4 |  |
| hsa-miR-320a | TRIM41 |  |
| hsa-miR-320a | TSPYL5 |  |
| hsa-miR-320a | NAP1L5 |  |
| hsa-miR-320a | CALN1 |  |
| hsa-miR-320a | ARMCX2 |  |
| hsa-miR-320a | PPP2R2C |  |
| hsa-miR-320a | NPAS2 |  |
| hsa-miR-320a | DAZAP1 |  |
| hsa-miR-320a | AZIN1 |  |
| hsa-miR-320a | RHOBTB1 |  |
| hsa-miR-320a | DNER |  |
| hsa-miR-320a | DPY30 |  |
| hsa-miR-320a | PBX3 |  |
| hsa-miR-320a | NR3C2 |  |
| hsa-miR-320a | RHOG |  |
| hsa-miR-320a | ZC3H7B |  |
| hsa-miR-320a | CRKL |  |
| hsa-miR-320a | LMO3 |  |
| hsa-miR-320a | AP3M1 |  |
| hsa-miR-320a | SEMA6D |  |
| hsa-miR-320a | RAB18 |  |
| hsa-miR-320a | HNRNPF |  |
| hsa-miR-320a | CREB5 |  |
| hsa-miR-320a | ARPC5 |  |
| hsa-miR-320a | DHDDS |  |
| hsa-miR-320a | ESRRG |  |
| hsa-miR-320a | AKT3 |  |
| hsa-miR-320a | ENAH |  |
| hsa-miR-320a | SASH1 |  |
| hsa-miR-320a | SMG7 |  |
| hsa-miR-320a | HIVEP2 |  |
| hsa-miR-320a | HIPK1 |  |
| hsa-miR-320a | SLC16A1 |  |
| hsa-miR-320a | SLC10A3 |  |
| hsa-miR-320a | RAP1A |  |
| hsa-miR-320a | TAF5 |  |
| hsa-miR-320a | INA |  |
| hsa-miR-320a | STAG2 |  |
| hsa-miR-320a | HECTD2 |  |
| hsa-miR-320a | BMPR1A |  |
| hsa-miR-320a | ATRX |  |
| hsa-miR-320a | NRP1 |  |
| hsa-miR-320a | SHISA7 |  |
| hsa-miR-320a | FLRT3 |  |
| hsa-miR-320a | IPO7 |  |
| hsa-miR-320a | MLLT3 |  |
| hsa-miR-320a | WRNIP1 |  |
| hsa-miR-320a | MIER3 |  |
| hsa-miR-320a | YWHAQ |  |
| hsa-miR-320a | COPS2 |  |
| hsa-miR-320a | GRB2 |  |
| hsa-miR-320a | POGZ |  |
| hsa-miR-320a | BANP |  |
| hsa-miR-320a | NR3C1 |  |
| hsa-miR-320a | PPM1A |  |
| hsa-miR-320a | YWHAZ |  |
| hsa-miR-320a | TMEM106B |  |
| hsa-miR-320a | FKBP1A |  |
| hsa-miR-320a | sep15 |  |
| hsa-miR-320a | FBXO11 |  |
| hsa-miR-320a | CLASP1 |  |
| hsa-miR-320a | PPM1B |  |
| hsa-miR-320a | TROVE2 |  |
| hsa-miR-320a | CPEB1 |  |
| hsa-miR-320a | RASA1 |  |
| hsa-miR-320a | TUSC3 |  |
| hsa-miR-320a | DBN1 |  |
| hsa-miR-320a | DAG1 |  |
| hsa-miR-320a | FAM49B |  |
| hsa-miR-320a | LHFPL3 |  |
| hsa-miR-320a | TXNDC5 |  |
| hsa-miR-320a | YTHDF3 |  |
| hsa-miR-320a | KLHL36 |  |
| hsa-miR-320a | TLK2 |  |
